# Multi-dimensional charge transport in supramolecular helical foldamer assemblies†
†Electronic supplementary information (ESI) available: Details of the synthesis of compounds **Q5–Q33** and **AQ5–AQ9**, protocols for surface modification and characterization, *I*/*V* curves for vertical and horizontal charge transport mechanisms and theoretical calculations. See DOI: 10.1039/c7sc03341a



**DOI:** 10.1039/c7sc03341a

**Published:** 2017-09-13

**Authors:** Alejandro Méndez-Ardoy, Nagula Markandeya, Xuesong Li, Yu-Tang Tsai, Gilles Pecastaings, Thierry Buffeteau, Victor Maurizot, Luca Muccioli, Frédéric Castet, Ivan Huc, Dario M. Bassani

**Affiliations:** a Univ. Bordeaux CNRS UMR 5255 ISM , 351, Cours de la Libération , 33405 Talence , France . Email: dario.bassani@u-bordeaux.fr; b Univ. Bordeaux CNRS UMR 5248 CBMN , 2 rue Escarpit , 33600 Pessac , France . Email: i.huc@iecb.u-bordeaux.fr; c Inst. Polytechnique de Bordeaux CNRS UMR 5629 LCPO , 16, Av. Pey-Berland , 33600 Pessac , France

## Abstract

Aromatic foldamers are bioinspired architectures whose potential use in materials remains largely unexplored. Here we report our investigation of vertical and horizontal charge transport over long distances in helical oligo-quinolinecarboxamide foldamers organized as single monolayers on Au or SiO_2_. Conductive atomic force microscopy showed that vertical conductivity is efficient and that it displays a low attenuation with foldamer length (0.06 Å^–1^). In contrast, horizontal charge transport is found to be negligible, demonstrating the strong anisotropy of foldamer monolayers. Kinetic Monte Carlo calculations were used to probe the mechanism of charge transport in these helical molecules and revealed the presence of intramolecular through-space charge transfer integrals approaching those found in pentacene and rubrene crystals, in line with experimental results. Kinetic Monte Carlo simulations of charge hopping along the foldamer chain evidence the strong contribution of multiple 1D and 3D pathways in these architectures and their dependence on conformational order. These findings show that helical foldamer architectures may provide a route for achieving charge transport over long distance by combining multiple charge transport pathways.

## Abstract 2

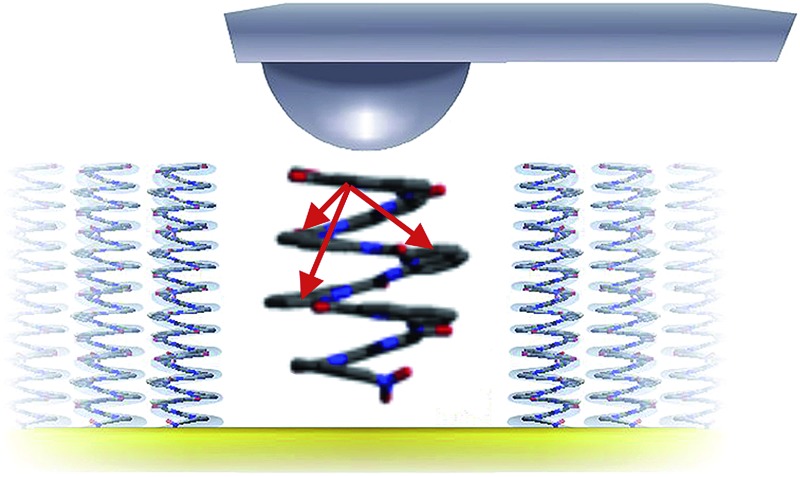
Helical aromatic foldamers are bioinspired architectures that combine through-bond and through-space charge transport in a single molecule.

## 1 Introduction

Understanding charge transport by organic molecules is crucial for numerous fundamental processes and technological applications. Molecular crystals and polymers, with charge carrier mobilities that can approach those of amorphous silicon,^1,2^ are the most commonly investigated systems even though structural order is crucial in determining the performance.^3–7^ In the case of conjugated molecules in Metal–Organic–Metal (MOM) junctions, the distance dependence of the conductivity obeys a semi-logarithmic equation associated with non-resonant tunneling in which the junction resistance scales to *R* = *R*_0_ exp(*βd*) where *R*_0_ is the contact resistance, *d* is the distance, and *β* a constant that depends on the molecular structure. Typical values for *β* (Å^–1^) range from 1.0–1.2 for alkanes,^8,9^ 0.04–0.17 for porphyrins,^10,11^ to 0.4 and 0.2 for oligophenylenes^9^ and carotenoids,^12^ respectively, and 0.1 for oligothiophenes.^13^ In non-conjugated (macro) molecules such as DNA, charge transport occurs *via* superexchange over short distances (*β* ≈ 0.9 Å^–1^)^14^ and through a delocalized hopping mechanism over longer distances.^15^ A similar case was found for oligophenyleneimines, where a transition between a tunneling regime (*β* = 0.3 Å^–1^) to a hopping mechanism (*β* ≈ 0.09 Å^–1^) was observed for *d* > 4 nm.^16^ Although *β* is not strictly applicable for a pure hopping mechanism which should not necessarily follow the well-known exponential decay of transmission rate with distance, Ratner *et al.* showed that the presence of competing loss mechanisms leads to the recovery of the usual exponential behavior but with a lower attenuation factor.^17^ This is the case for duplex DNA^18^ and the observation of low values of *β* is currently regarded as a fingerprint of an underlying hopping mechanism.^19^

Compared to bulk materials, where multiple charge transport pathways are present,^20^ molecular wires are limited to a single, generally through-bond, pathway. This renders them particularly sensitive to defects or conformational reorganization as these can significantly hamper charge transport by blocking the only pathway available.^21,22^ Nonetheless, supramolecular wires are interesting as they can accommodate the construction of heterojunctions or other functionality within a 1D framework.^23–27^ For this reason, the development of molecules capable of combining both through-space and through-bond charge transport is of great interest as this combination may improve the performance and the stability of molecular devices. Along these lines, Chen *et al.* showed that a through-space charge transport can be significantly more efficient in short π-stacked tetraphenylene bridges than through-bond transport over a similar distance,^28^ whereas charge transport along the backbone of an oxahelicene comprising 5 conjugated aromatic units was reported to occur efficiently *via* a through-bond mechanism.^29^ Nevertheless, there has been no demonstration of combining multiple conductance pathways in a linear non-conjugated molecule to achieve charge transport over long distances. We now show that this can be achieved using aromatic helical foldamer assemblies.

**Fig. 1:**
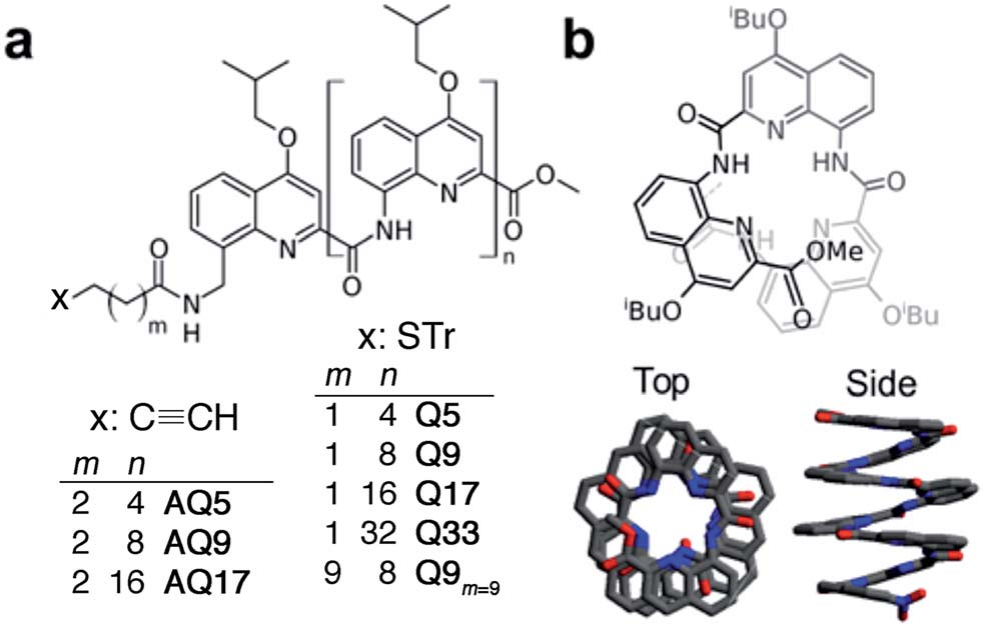
(a) Chemical formulae of oligoquinoline foldamers employed in this study (Tr = trityl). These foldamers differ in their molecular helix length (*n* = 4–32) and linker length (*m* = 1, 2 or 9), and in the anchoring group (thiol or alkyne); (b) schematic (top) and crystal (bottom) structures of helical oligo-quinoline carboxamide foldamers. In the crystal structure representation, isobutoxy side chains and included solvent molecules have been omitted for clarity.^51,52^

Synthetic foldamers are artificial folded architectures akin to the structures of biopolymers.^30,31^ Among these, aromatic foldamers – foldamers with aryl groups in their main chain – stand out because their structures are both predictable and stable.^32–34^ While early studies on aromatic foldamers focused on relatively small molecules, improvements in synthetic and self-assembly fabrication methods now allow for the delivery of engineered large (up to 25 kDa) and robust molecules or molecular assemblies defined with atomic precision across nanometric distances,^35,36^ aromatic foldamers have been shown to be useful for the purpose of molecular recognition or for biological applications but their potential use in materials remains largely unexplored.^31–34^ Interestingly, charge transport through a linearly-stacked oligoanthracene foldamer was investigated by Carini *et al.* at the single molecule level over distances spanning 0.3 to 1.2 nm to reveal a value of *β* = 0.02 Å^–1^, which could be promising for applications in molecular electronics.^37^ Several groups have used helical structures to control photoinduced charge separation and recombination in donor–acceptor couples through folded bridges in solution.^38–40^ Recent results based on photoinduced electron transfer rates suggest that helically-folded quinolinecarboxamide oligomers (Fig. 1) can mediate charge separation in the excited state over nanometric distances by providing intermediate hopping sites.^40,41^

Herein, we report a combined experimental and theoretical investigation of charge transport in self-assembled monolayers of helically-organized oligo-quinolinecarboxamide foldamers in the ground state that constitute a first example of a foldamer-based Metal–Organic–Metal (MOM) junction. Conductive-probe AFM (C-AFM) was used to investigate the effects of self-organization on both vertical and horizontal charge transport between a conductive tip and a fixed Au electrode. C-AFM is a versatile technique to determine the resistance across self-assembled monolayers,^4,42–44^ including complex systems incorporating proteins,^45^ DNA,^46–48^ organometallic complexes,^49^ or fullerenes.^50^ Whereas we found no evidence of lateral charge transport, vertical charge transport across the length of the foldamer chain was found to be particularly efficient. The results show a remarkably low value for the apparent attenuation factor (*β* = 0.06 Å^–1^) in agreement with a mechanism that, according to kinetic Monte Carlo simulations, greatly benefits from the combination of multiple hopping pathways between adjacent as well as remote subunits thanks to charge transfer integrals approaching those found in pentacene and rubrene crystals. These findings show that aromatic helical foldamers provide a new strategy for maximizing charge transport over long distances that is not contingent on extending molecular conjugation or intermolecular packing.

## 2 Results and discussion

### Vertical charge transport

To evaluate the efficiency of charge transport along the helical structure of the foldamers, we used conductive AFM to study length-dependent charge transport by formation of molecular junctions between the AFM tip and self-assembled monolayers of the helical architectures on gold substrates.

Compounds **Q5**, **Q9**, **Q17** and **Q33** (Fig. 1) constitute a set of well-defined helically-folded oligo-quinolinecarboxamides terminated with a trityl-protected thiol group at the N-terminus (see ESI† for details about the synthesis). Molecular length is determined by increasing the number of quinoline units from *n* = 5 to 33. The N-terminal benzyl amide group was introduced because of its propensity to form a 90° turn^53,54^ with respect to the last aryl ring, favoring the orientation of the thiol-bearing alkyl chain parallel to the helix axis so as to allow the helices to stand upright, with low tilt angles with respect to the Au surface. The robustness of these molecular helices both in solution and in the solid state is due to electrostatic repulsions between heteroatoms in adjacent quinoline units, as well as intramolecular hydrogen bonds and aromatic stacking between consecutive turns (Fig. 1b), and has been abundantly demonstrated by X-ray diffraction on single crystals and high-resolution NMR spectroscopy.^51,52^ The helices are characterized by a helical pitch (vertical rise) of 3.5 Å per turn and a curvature of 2.5 units per turn (as determined from the solid-state structures).^52^ Considering a linker distance of 0.4 nm, we calculated the overall lengths of **Q5**, **Q9**, **Q17**, and **Q33** in their folded conformations to be 1.2, 1.8, 3.1, and 5.7 nm, respectively. No evidence for excitonic interactions between the electronic transitions of the quinoline units, *e.g.* analogous to those seen in *e.g.* DNA,^55^ is observed in the absorption or emission spectra (Fig. S3†).

**Fig. 2:**
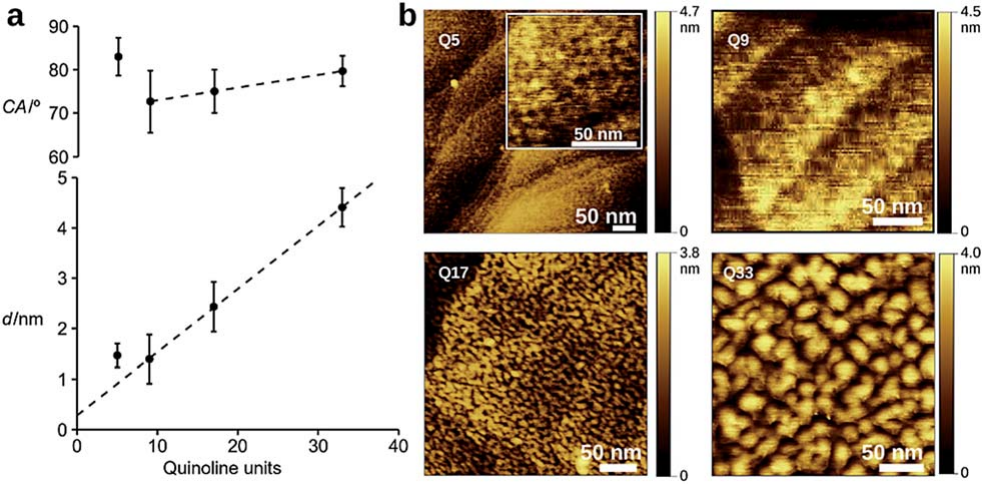
(a) Water contact angle (CA) and film thickness (*d*) determined by ellipsometry. Values represent the average of three independent monolayer preparations ± *σ*, dashed line is the linear regression for *n* ≥ 9. (b) Height images obtained by AFM for SAMs of **Q5–Q33** on Au.

Dense self-assembled monolayers of the thiols on flat Au(111) substrates were obtained after *in situ* deprotection of the trityl group in acid followed by incubation at 50 °C for 48 h (see ESI† for experimental details).^56^ Cyclic voltammetry shows complete passivation of the underlying Au electrode as expected for the formation of dense monolayers (Fig. S5†). Contact angle measurements, ellipsometry, and polarization modulation – IR reflection absorption spectroscopy (PMIRRAS, Fig. S6 and Table S5†) are all in agreement with the formation of monolayers in which the helices are oriented perpendicular to the surface of the substrate. Indeed, a clear linear dependence of the film thickness *vs. n* is observed for **Q9**, **Q17**, and **Q33** (Fig. 2), where the intercept gives a linker length (0.3 nm) in agreement with molecular modeling (0.4 nm). In contrast, a monolayer in which the foldamer assemblies lie horizontal to the surface would not provide a regular increase in surface height with increasing foldamer length. The tilt angle of the assembly is calculated to be 35°, as typically observed for alkane-thiols on Au(111) surfaces.^57^ Furthermore, as expected for the exposure of identical headgroups, the molecular packing generates a hydrophobic surface that is similar for all compounds (Fig. 2). A notable exception to this trend is the shortest foldamer, **Q5**, for which somewhat higher than expected values of the thickness and contact angle are obtained. Short foldamer sequences possess a low aspect ratio that resembles a disc-like structure compared to the rod-like longer foldamers. Therefore, **Q5** may exhibit different surface packing compared to **Q9–Q33**.

Investigation of the surface topology of **Q5–Q33** monolayers was carried out by AFM (Fig. 2b and S7†), which gave no evidence towards the formation of crystalline domains.^52^ We also observe increasing roughness upon increasing the foldamer length, which may originate from lateral organization of the foldamers due to van der Waals interactions between the alkyl side chains. A comparison between **Q9** and **Q9**_*m*=9_ showed that the length of the linker has a clear influence on the film thickness, but that measurements for water contact angle, capacitance, and passivation of the electrode towards an external redox probe are very similar (Table S4, Fig. S5†), suggesting similar surface coverage. Based on this, we chose to use the *m* = 1 linker for all other compounds because it provides good surface coverage while minimizing contact resistance.^58,59^

**Fig. 3:**
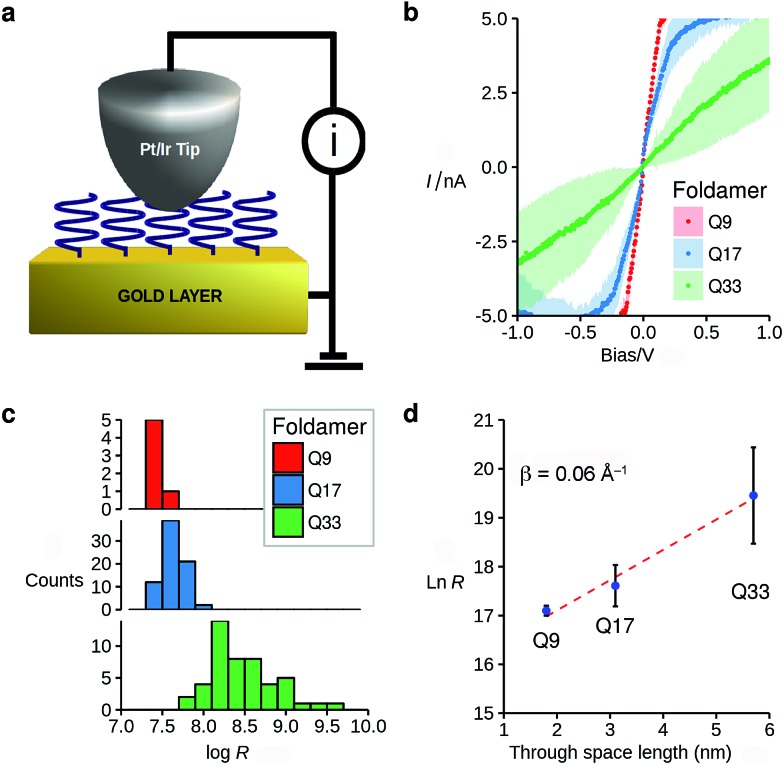
(a) Schematic representation of the MOM junction prepared using C-AFM and (b) typical *I*–*V* curves (bias ± 1 V) obtained from monolayers **Q9–Q33** (1 nN tip force); (c) histograms of resistance obtained from the slope of the *I*–*V* curves at low bias. (d) Dependence of the vertical resistance (*Ω*) *vs.* molecular length for **Q9–Q33** (median ± absolute deviation).

The vertically aligned foldamers on gold form part of a molecular junction designed to probe charge transport across the helical architectures. The circuit is completed using a conductive AFM equipped with a Pt–Ir tip that allows controlling the contact force and applied voltage while monitoring the current across the monolayer (Fig. 3a). Good electrical contact was obtained by applying a light force to the tip (<1.5 nN) in order to avoid molecular compression and monolayer invasion. Upon increasing the tip force to about 4 nN, short-circuit junctions became predominant which indicates deformation of the monolayer or direct contact between the tip and the surface. Current intensity *versus* applied bias (±1.5 V) curves were collected at different points at the surface, with each point measured in triplicate (see ESI†). To allow comparison between the different samples, all the measurements were conducted with the same tip at the same applied force. Averaged *I*–*V* curves for **Q9–Q33** are shown in Fig. 3b. The junction resistance is calculated in the region where *I* scales linearly with the applied bias (±0.3 V), which reduces the effect of fluctuations at higher values of *V*.

The resistance of the MOM junctions is found to increase with the number of quinoline units, as expected for charge transport through a molecular junction. The resistances obtained from measurements on each SAM are combined in histograms (Fig. 3c), which show a tight distribution for **Q9** and **Q17**, and a broader distribution for **Q33**.^60^ In the latter case, the increased roughness of the SAM observed by AFM may explain the broader distribution. Following the discussion by Whitesides and co-workers,^61^ we use the median of the distribution of the resistance as it is less sensitive to outlying values.^62^ It is immediately apparent that there is only a very small dependence of the junction resistance on molecular length. A plot of ln *R vs.* molecular length for the series **Q9–Q33** is linear with a slope of 0.06 ± 0.015 Å^–1^ (Fig. 3d). As discussed in the introduction, such a low value is typical of charge transport by hopping, which must be efficient despite the absence of significant orbital overlap between adjacent quinoline units.

### Horizontal charge transport

**Fig. 4:**
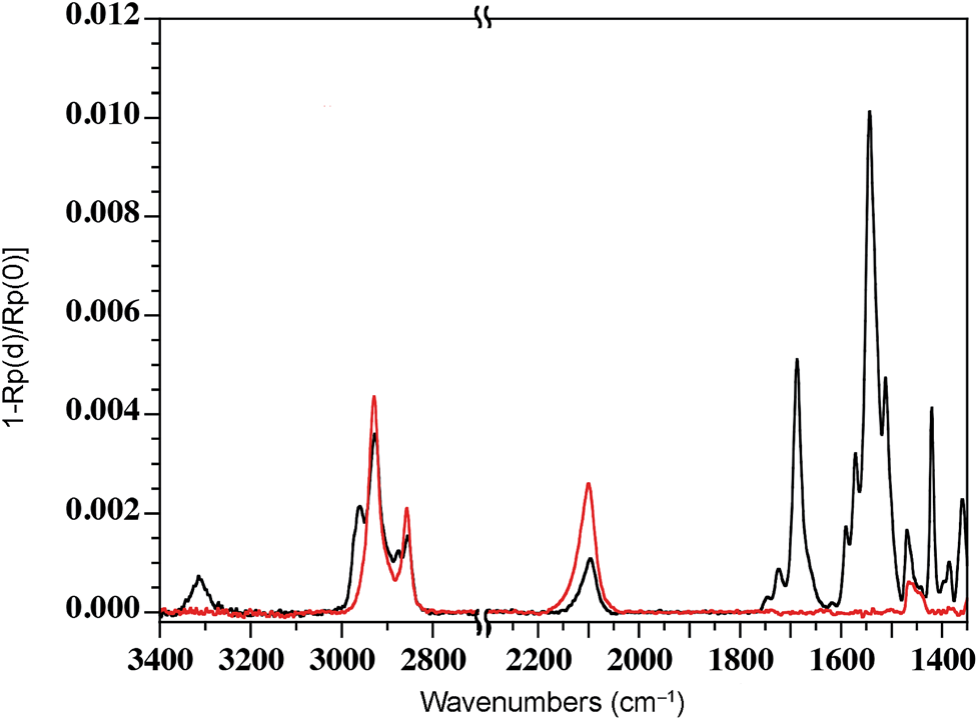
PMIRRAS spectrum of a SiO_2_ substrate grafted with C_11_H_22_N_3_ before (red curve) and after (black curve) click reaction with **AQ5**. Covalent grafting is evidenced by the decrease of the azide stretching band at 2100 cm^–1^.

We have previously shown that dense self-assembled monolayers of conjugated organic molecules can support lateral charge transport over distances attaining several hundred nanometers.^63^ To test whether this is the case for the foldamer monolayers, we used a similar set-up in which a foldamer monolayer is formed through a CuAAC reaction between an alkyne-terminated foldamer and an azide-terminated undecyl monolayer on SiO_2_. Foldamers composed of 5, 9, or 17 quinoline units (**AQ5**, **AQ9**, **AQ17**) terminated with an alkyne chain were prepared analogously to the thiol-appended foldamers **Q5–Q17** (see ESI†). These were then immobilized onto azide-terminated Si/SiO_2_ (1.9 nm) substrates prepared by grafting 11-bromoundecyltrichlorosilane followed by reaction with sodium azide in DMF. Covalent grafting was achieved using a solution of the foldamer (0.5 mM) dissolved in DMF containing CuSO_4_, ascorbic acid, and TBTA. After incubation, the substrates were rinsed with DMF, DCM, EtOH, H_2_O and EtOH, then blow-dried with a stream of Ar. Evidence for covalent grafting of the foldamer through triazole formation is provided by PMIRRAS (Fig. 4), which shows a decrease of the azide stretching band at 2100 cm^–1^.^64^ From this, it is estimated that *ca.* 65% of the azide groups reacted giving a surface packing similar to that previously obtained using a polyaromatic receptor (of similar lateral dimensions as the foldamers) shown to be oriented orthogonal to the surface.^65^ In agreement with this and with the results obtained from grafting onto gold substrates discussed above, analysis of the ellipsometric constants (Fig. S8†) gives an optical thickness that increases with increasing number of quinoline units. In the case of **AQ17**, a total thickness of 2.6 nm is obtained, which is in good agreement with the value obtained for **Q17** on Au (2.2 nm) when one takes into account the longer linker used when binding onto SiO_2_.

**Fig. 5:**
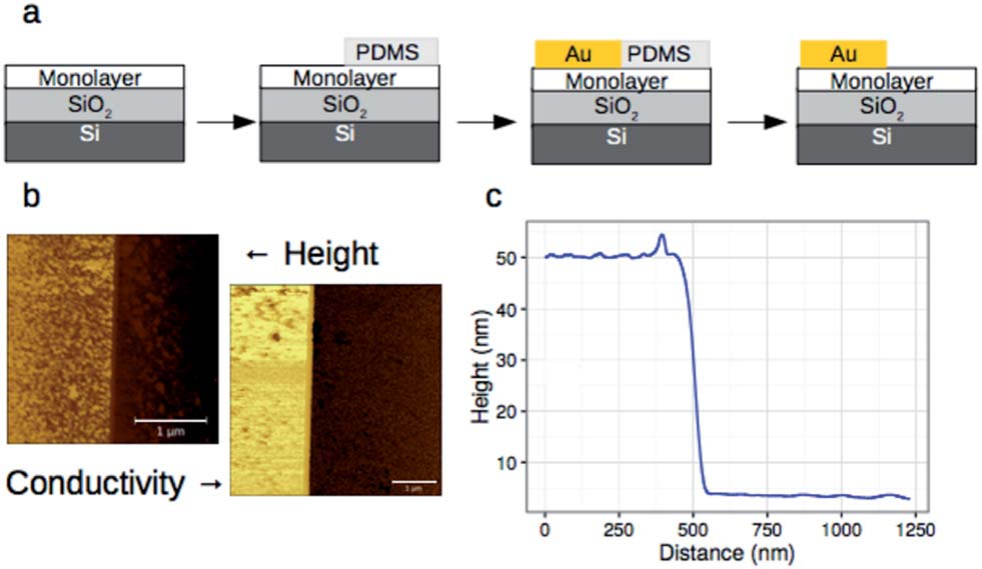
(a) Procedure for the deposition of a fixed 50 nm thick gold electrode using a soft PDMS contact shadow mask and (b) height and conductivity images obtained using a C-AFM setup and a monolayer of 11-azidoundecane (white scale bar is 1 μm). (c) Height profile of the electrode–monolayer interface.

To investigate lateral charge transport, substrates grafted with **AQ17** were selected in view of the latter's synthetic accessibility and sufficient foldamer length to allow for electronic interactions between molecules. A 50 nm gold electrode was evaporated onto the modified substrates using a soft PDMS contact shadow mask to protect the monolayer and obtain a sharp edge as described previously (Fig. 5).^63^ Substrates with a thick (200 nm) layer of SiO_2_ were used to ensure that no leakage current could flow through the substrate. The samples were mounted on the C-AFM and a conductive tip was used to complete the circuit and allow measurement of *I*/*V* curves at varying distances from the fixed Au electrode (Fig. 5c).

The results obtained for the **AQ17** monolayer indicated a complete absence, under the experimental conditions, of lateral conductivity in contrast to our previous results using anthracene- or fullerene-containing monolayers. The behavior of the **AQ17** monolayer was identical to that of an insulating alkane monolayer on SiO_2_.^63^ As a test of the instrumental set-up, control experiments with the 11-azidoundecane monolayer evidenced a semi-logarithmic decrease of the conductivity over a 100 nm region separating the tip from the fixed electrode (Fig. S12†), attributable to a small degree of charge transport by the monolayer or by residual adsorbed water. We therefore attribute the absence of lateral conductivity by the foldamer monolayer to originate from insufficient electronic communication between the adjacent foldamer architectures, either due to low surface density of the foldamers or to intrinsically low values of the lateral charge carrier mobility. The latter explanation seems more plausible in view of the isobutyl side-chains, which prevent close contact of the aromatic regions. Indeed, Mayor, Calame, and co-workers have previously shown that close contact is important for intermolecular charge transport through π-stacking interactions in single molecule-based break-junctions.^66^ Typical packing of isobutoxy-substituted oligo-quinolinecarboxamide foldamers observed in the solid state shows interdigitation of the isobutoxy chains that prevents lateral contacts, while longitudinal contacts remain possible through end-to-end stacking of the helices.^67^

### Molecular modeling

**Fig. 6:**
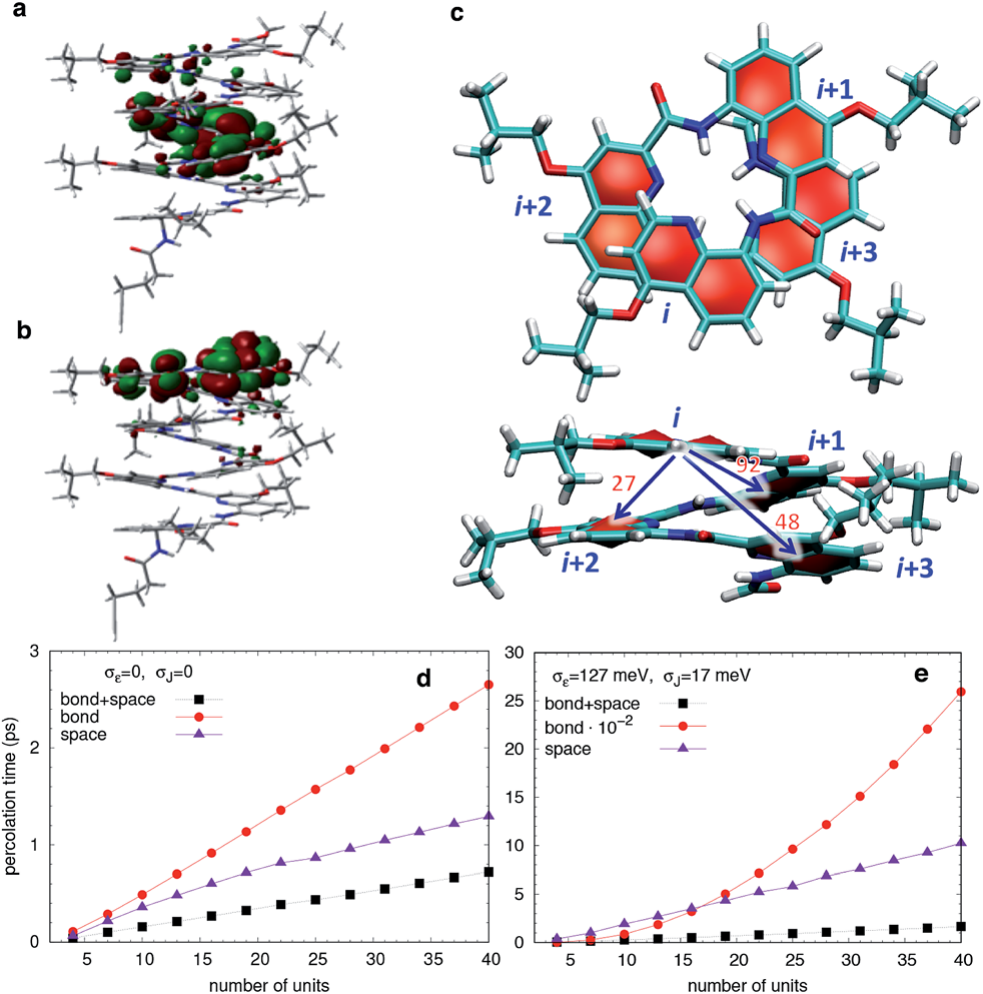
(a) HOMO and (b) LUMO calculated at the PBE0-D3BJ/6-31G(d) level for **Q9**. Isovalues = 0.015 au. (c) Top and side view of a tetramer showing the spatial distribution of the quinoline units along the helix and the average values of the electronic couplings (meV) between first (*i*, *i* + 1), second (*i*, *i* + 2) and third (*i*, *i* + 3) neighbors. The coupling between fourth neighbors is instead negligible. Calculated percolation times for a hole upon application of an electric field of 1 V nm^–1^, as a function of helix length *n*, in absence (d) or presence (e) of Gaussian disorder in energetic levels and transfer integrals. The labels “bond” and “space” refer to simulations where only (*i*, *i* + 1), or (*i*, *i* + 2) and (*i*, *i* + 3) couplings are active, respectively.

To understand how vertical charge transport can be so efficient in a helically-folded molecular strand, we performed DFT calculations at the PBE0-D3BJ/6-31G(d) level^68,69^ (see Fig. S1 and S2† for calculated electronic absorption spectra). The ionization potentials (IP) and electron affinities (EA) are collected in Table S1† along with the HOMO and LUMO energies, the reorganization energies for electrons (*λ*_e_) and holes (*λ*_h_), and the maximal absorption wavelengths (*λ*_max_). The rapid convergence of the electronic and optical properties with the number of repeating units is consistent with the weak delocalization (over 2–3 units) of the Frontier MOs along the foldamers as expected for oligo(arylamide)s (Fig. 6 and S4†). The reorganization energies are significantly larger for electrons than for holes while EA values are modest, indicating that these systems should favour hole transport over electron transport.

In principle, a helical structure allows for both superexchange and hopping mechanisms to take place simultaneously.^70,71^ To investigate these possibilities, we evaluated the transfer integrals between neighboring and distal units. Not unexpectedly, there is moderately strong through-bond coupling between the *i*, *i* + 1 units (92 meV) which allows charges to move along the helix by following the oligoquinoline chain. However, due to the specific spatial arrangement of quinoline units in the helix, there is also strong through-space coupling between non-directly bonded units situated above and below the quinoline plane, *i.e.* between *i*, *i* + 2 (27 meV) and *i*, *i* + 3 (48 meV) units (Fig. 6c). This electronic coupling, reported in Table S2,† contributes at least in principle to increasing superexchange-mediated transfer rates, and also gives rise to thermally activated hopping. Indeed, the *i*, *i* + 3 coupling of 48 meV is roughly half of the coupling reported at the same level of calculation for one of the best organic semiconductors, rubrene,^72^ and very similar to couplings present in π-stacked pentacene molecular crystals.^73^ The coupling between *i*, *i* + 4 is instead negligible.

The calculated energetic disorder in the HOMO levels (*σ*_ε_ = 127 meV) is larger than the through-bond coupling (*J*_*i*,*i*+1_ = 92 meV for the *n* = 48 helix), hinting at the predominance of hopping for long architectures. Accordingly, we studied hole transport using Kinetic Monte Carlo (KMC, see ESI† for details of calculations) simulations based on the Marcus hopping model, with the objective of disentangling the contributions of the different electronic couplings. We compared three different situations: one where only the nearest-neighbor coupling is switched on (“bond”), one where only the two through-space couplings are active (“space”), and finally one where all the couplings are present (“bond” + “space”), both in presence and in absence of energetic disorder. The calculated percolation times, directly proportional to the electric resistance, are plotted in Fig. 6 for helices of increasing length. As expected from the hopping model, the times increase with length, confirming the consistency of the KMC results. Surprisingly, the through-bond percolation times are always larger than the through-space ones (even in absence of disorder) indicating that the larger number of through-space pathways is more important to charge transport than the larger through-bond coupling. This effect is of course magnified when disorder is present (Fig. 6e). In addition, percolation rates when both mechanisms are present are not simply the sum of the rates of the individual contributions. Instead, they show a strongly synergistic effect arising from the combination of the two mechanisms, resulting in calculated hole mobilities of 0.1–1 cm^2^ V^–1^ s^–1^, depending on the amount of disorder.

## 3 Conclusions

From the experimental and theoretical results, it emerges that the combination of through-space and through-bond charge transport channels is an essential component in designing efficient 1D semiconducting materials. Therefore, charge transport in helical molecules should not be viewed as occurring solely through-bond or through-space, but as a dynamic combination of the two, whose relative contribution may be modulated by the conformational dynamics of the structure. The helical nature of the foldamers used in this study offers an easy route to this highly desired feature, allying directional transport of 1D materials with the higher dimensionality of 2D and 3D materials.^71^ Furthermore, the synthetic flexibility in these architectures allows for the realization of 3D supramolecular aggregates *via* the implementation of molecular recognition sites both on the periphery and within the helix, as well as the possibility of fabricating double helix and three-way junctions. The absence of lateral charge transport in foldamer monolayers suggest that they may behave as insulated molecular wires, promoting unidirectional charge transport along their helical axis.

## Conflicts of interest

There are no conflicts to declare.
