# Supplementary material for: Multi-dimensional charge transport in supramolecular helical foldamer assemblies
Source: Chem Sci. 2017 Sep 13;8(10):7251–7. doi: 10.1039/c7sc03341a (PMC5633016; doi:10.1039/c7sc03341a)
Supplement: Supplementary file 1 [file SC-008-C7SC03341A-s001.pdf]

Electronic Supplementary Material (ESI) for Chemical Science.
This journal is © The Royal Society of Chemistry 2017

Supplementary Information for:

Multi-dimensional charge transport in supramolecular helical foldamer assemblies

Alejandro Mendez-Ardoy,[a] Markandeya Nagula,[b] Xuesong Li,[b] Yu-Tang Tsai,[a] Gilles Pecastaings,[c] Thierry Buffeteau,[a] Victor Maurizot,[b] Luca Muccioli,[a,c] Frédéric Castet,[a] Ivan Huc,*[b] Dario M. Bassani.*[a]

[a]Univ. Bordeaux CNRS UMR 5255 ISM. 351, Cours de la Libération, 33405 Talence, France
E-mail: dario.bassani@u-bordeaux.fr
[b]Univ. Bordeaux CNRS UMR 5248 CBMN, 2 rue Escarpit, 33600 Pessac, France
E-mail: i.huc@iecb.u-bordeaux.fr
[c]Inst. Polytechnique de Bordeaux CNRS UMR 5629 LCPO, 16, Av. Pey-Berland, 33600 Pessac, France

Table of contents

Experimental and computational details 2
Characterization of the monolayers 16
Supplementary analysis of the resistance distributions. 19
NMR spectra 22
I-V curves 35
References 58

Experimental and computational details

Materials and methods. All reactions that required anhydrous conditions were carried out under a dry nitrogen atmosphere. Commercial reagents were purchased from Sigma-Aldrich or Alfa-Aesar and were used without further purification unless otherwise specified. Tetrahydrofuran (THF) was dried over alumina columns; chloroform, triethylamine and *N,N*-diisopropylethylamine (DIPEA) were distilled over calcium hydride (CaH_2) prior to use. Reactions were monitored by thin layer chromatography (TLC) on Merck silica gel 60-F254 plates and observed under UV light. Column chromatography purifications were carried out on Merck GEDURAN Si60 (40-63 μm). Preparative recycling GPC (gel permeation chromatography) was performed on a set of 1H, 1.5H, 2.5H and 3H columns (JAIGEL 20×600 mm) JAIGEL 20*600 mm columns (Japan Analytical Industry) in chloroform/0.5~1% ethanol, as mobile phase, with a flow rate of 3.5 mL/min. The monitoring UV detector was a UV-600 NEXT. High resolution mass spectra were obtained in the positive ion mode on a TOF spectrometer from the Mass Spectrometry Laboratory at the European Institute of Chemistry and Biology (UMS3033 & US001, IECB), Pessac, France. NMR spectra were recorded on an Avance II NMR spectrometer (Bruker Biospin) with a vertical 7.05T narrow-bore/ultrashield magnet operating at 300 MHz for ^{1}H observation, and 75 MHz for ^{13}C observation. Chemical shifts are reported in parts per million (ppm, δ) relative to the ^{1}H residual signal of the deuterated solvent used. ^{1}H NMR splitting patterns with observed first-order coupling are designated as singlet (s), broad singlet (bs), doublet (d), triplet (t), quartet (q), multiplet (m), doublet of doublet (dd), doublet of quartet (dq), doublet of triplet (dt) and triplet of doublet (td). Coupling constants (*J*) are reported in Hertz.

Synthesis of foldamers.

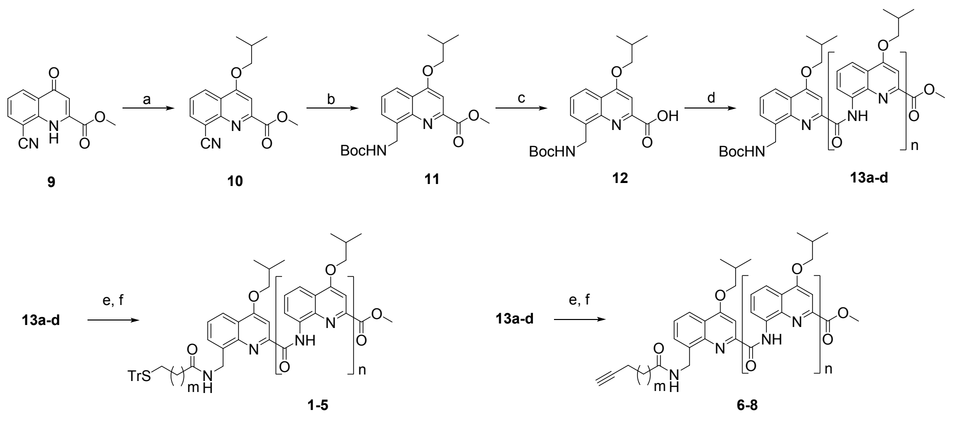

	Q5	Q9	Q17	Q33	$Q9_{m=9}$
n	4	8	16	32	8
m	1	1	1	1	9
compound #	**1**	**2**	**3**	**4**	**5**

	AQ5	AQ9	AQ17
n	4	8	16
m	2	2	2
compound #	**6**	**7**	**8**

Scheme S1. Synthesis of foldamers **Q5**, **Q9**, **Q17**, **Q33**, and $\mathbf{Q9_{m=9}}$ (compounds **1**-**5**) and AQ5, AQ9 AQ17(compounds **1**-**5**). Reagents and Conditions**:** a) isobutyl alcohol, triphenyl phosphine, di-isopropyl-azo-dicarboxylate (DIAD), dry THF, overnight; b) Raney Ni/H_2, Boc_2O, THF/MeOH, rt, overnight; c) NaOH, THF/MeOH (9:1 vol/vol), rt, 3h; d) 1-chloro-N,N,2-trimethyl-1-propenylamine, di-isopropyl-ethyl-amine (DIPEA),Oligomer amine $QnNH_2$, $CHCl_3$, 24h; e) TFA/$CHCl_3$ (1:1, vol/vol), rt, 3h; f) Anchoring carboxylic acid, PyBOP, DIPEA, $CHCl_3$, 40 °C overnight.

Compound Q5 (1). Compound **13a** (85 mg, 0.062 mmol) was stirred in TFA/$CHCl_3$ (2 mL, 50:50 vol/vol) at 25 °C for 3h. TFA was removed and azeotroped with toluene by rotary evaporation under reduced pressure. Then, the crude amine was re-dissolved in dry $CHCl_3$ (4 mL), and commercial 3-(tritylthio)-propionic acid (70 mg, 0.203 mmol), PyBOP (123 mg, 0.236 mmol) and DIPEA (0.049 mL, 0.338 mmol) were added and the reaction mixture was stirred at 40 °C overnight. The reaction mixture was washed with a saturated aqueous bicarbonate solution and an ammonium chloride solution, successively. The organic phase was dried on Na_2SO_4, filtered, and the filtrated was evaporated. The residue was subject to preparative GPC to yield pure **1** as a light yellow solid (85 mg, 85%). ^1H NMR ($CDCl_3$, 300 MHz) δ ppm = 11.92 (1H, s), 11.80 (2H, s), 11.55 (1H, s), 8.56 (2H, d, *J* = 7.47*)*, 7.97-8.23 (6H, m), 7.85 (1H, d, *J* = 8.15*)*, 7.68 (2H, t, *J* = 8.15*)*, 7.27-7.47 (5H, m), 6.99-7.23 (15H, m), 6.84 (1H, s), 6.74 (1H, s), 6.70 (1H, d, *J* =7.47*)*, 6.57 (1H, s), 4.30-4.47 (3H, m), 4.00-4.27 (3H, m), 3.90-4.00 (3H, m), 3.85 (1H, d, *J* = 7.49*)*, 3.80 (2H, d, *J* = 6.24*)*, 3.41-3.53 (1H, dd, *J* = 15.40, 6.66 Hz), 3.19 (3H, s), 2.43-2.60 (3H, m), 2.25-2.42 (3H, m), 2.04-2.19 (3H, m), 1.15-1.35 (30H, m). ^{13}C NMR ($CDCl_3$, 75 MHz): δ= 170.23, 163.93, 163.71, 163.31, 163.18, 163.09, 162.39, 161.82, 161.76, 161.07, 161.05, 150.70, 150.37, 149.83, 148.97, 145.53, 144.71, 144.69, 139.16, 138.31, 137.86, 137.34, 135.71, 134.18, 133.61, 133.58, 129.50, 127.95, 127.80, 127.72, 127.19, 127.11, 126.83, 126.50, 125.87, 122.68, 122.54, 121.97, 121.87, 121.56, 121.38, 117.14, 117.09, 117.02, 116.49, 116.27, 116.04, 115.84, 100.31, 99.64, 98.90, 97.95, 97.78, 75.63, 75.52, 75.25, 75.22, 75.09, 66.57, 52.15, 45.78, 38.80, 35.14, 28.41, 28.28, 28.20, 27.21, 19.58, 19.50, 19.45, 19.39, 19.34, 9.17. HRMS (ESI$^+$): *m/z* calcd for $C_{94}H_{95}N_{10}O_{12}S$ $[M+H]^+$ 1588.6885 found 1588.6872.

Compound Q9 (2). Compound **13b** (250 mg, 0.107 mmol) was stirred in TFA/$CHCl_3$ (3 mL, 50:50 vol/vol) at 25 °C for 3h. TFA was removed and azeotroped with toluene by rotary evaporation under reduced pressure. Then, the crude amine (77 mg) was re-dissolved in dry $CHCl_3$ (4 mL), and commercial 3-(tritylthio)propionic acid (30 mg, 0.086 mmol), PyBOP (44.7 mg, 0.086 mmol) and DIPEA (0.025 mL, 0.173 mmol) were added and the reaction mixture was stirred at 40 °C overnight. Solvents were evaporated and the residue was subjected to silica gel column chromatography eluting with ethyl acetate/cyclohexane (25:75 vol/vol) to yield pure **2** as a light yellow solid (76 mg, 28 % overall yield). ^1H NMR ($CDCl_3$, 300 MHz) δ ppm = 11.43 (1H, s), 11.29 (1H, s), 11.13 (1H, s), 10.96 (1H, s), 10.89 (1H, s), 10.82 (1H,s), 10.76 (1H, s), 10.68 (1H, s), 8.16 (2H, d, *J* = 7.63 Hz), 7.96-8.12 (3H, m), 7.61-7.96 (9H, m), 7.56 (1H, d, *J* = 7.63*)*, 7.27-7.42 (5, m), 7.21 (2H, s), 7.11-7.18 (7H, m), 6.94-7.10 (12H, m), 6.91 (1H, s), 6.84 (1H, s), 6.66 (1H, s), 6.53 (1H, s), 6.49 (1H, s), 6.44 (1H, d, *J* = 7.18*)*, 6.40 (1H, s), 6.35 (1H, s), 6.18 (1H, s), 6.01 (1H, s), 4.08-4.16 (2H, m), 3.96-4.07 (2H, m), 3.73-3.94 (12H, m), 3.66 (2H, d, *J* = 6.39*)*, 3.50-3.63 (2H, m), 3,05-3.15 (1H, dd, *J* = 15.79, 6.03*)*, 2.99 (3H, s), 2.14-2.55 (10H, m), 1.93-2.09 (3H, m), 1.07-1.39 (m). ^{13}C NMR ($CDCl_3$, 75 MHz): δ= 170.22, 163.75, 163.03, 162.81, 162.76, 162.64, 162.42, 162.32, 161.96, 161.34, 161.23, 161.17, 160.57, 159.39, 159.25, 159.20, 158.67, 150.07, 149.65, 149.09, 149.06, 148.90, 148.68, 148.44, 145.06, 144.71, 144.63, 144.23, 138.70, 137.99, 137.63, 137.43, 137.38, 137.34, 136.92, 135.05, 133.57, 133.28, 132.99, 132.69, 132.50, 129.66, 129.45, 128.03, 127.76, 127.50, 126.81, 126.70, 126.47, 126.04, 125.89, 125.74, 125.66, 125.51, 122.52, 122.33, 122.26, 122.21, 122.03, 121.96, 121.57, 121.44, 121.38, 121.09, 117.17, 117.06, 116.84, 116.80, 116.60, 116.35, 116.16, 116.14, 115.77, 115.67, 100.06, 99.42, 99.01, 98.72, 98.65, 98.59, 97.65, 97.50, 75.46, 75.36, 75.26, 75.07, 75.02, 74.77, 67.16, 66.98, 66.49, 52.01, 38.77, 34.99, 33.32, 29.78, 28.29, 28.25,

28.21, 28.17, 28.14, 28.10, 28.05, 27.09, 26.74, 19.69, 19.61, 19.53, 19.47, 19.40, 19.35, 19.26. HRMS (ESI^+): *m/z* calcd for $C_{150}H_{150}N_{18}O_{20}S$ $[M+H]^+$ 2557.1106 found 2557.1113.

Compound Q17 (3). Compound **13c** (65 mg, 0.010 mmol) was stirred in TFA/$CHCl_3$ (2 mL, 50:50 vol/vol) at 25°C for 3h. TFA was removed and azeotroped with toluene by rotary evaporation under reduced pressure. Then, the crude amine was re-dissolved in dry $CHCl_3$ (3 mL), and commercial 3-(tritylthio)propionic acid (15 mg, 0.043 mmol), PyBOP (26 mg, 0.050 mmol) and DIPEA (0.010 mL, 0.072 mmol) were added and the reaction mixture was stirred at 40 °C overnight. Precipitation from MeOH and filtration yielded pure **3** as a light yellow solid (58 mg, 85 %). ^{1}H NMR ($CDCl_3$, 300 MHz) δ ppm = 11.11 (1H, s), 11.05 (1H, s), 10.72 (1H, s), 10.67 (1H, s), 10.49 (1H, s), 9.87-10.33 (11H, m), 7.94 (1H, d, *J* = 7.38*)*, 7.87 (1H, d, *J* = 7.38*)*, 7.69-7.82 (7H, m), 7.52-7.68 (12H, m), 7.20 (2H, t, *J* = 7.38*)*, 6.88-7.16 (31H, m), 6.64-6.88 (13H, m), 6.39 (2H, d, *J* = 10.06*)*, 6.29 (1H, d, *J* = 7.58*)*, 6.21 (1H, s), 6.14 (2H, d, *J* = 6.99*)*, 5.88 (2H, d, *J* = 13.25*)*, 5.68-5.79 (8H, m), 3.84-3.98 (4H, m), 3.60-3.78 (20H, m), 3.39-3.58 (12H, m), 2.86 (1H, dd, *J* = 15.52, 5.82 Hz), 2.83 (3H, s), 1.79-2.43 (21H, m), 1.04-1.24 (102H, m). HRMS (ESI^+): *m/z* calcd for $C_{262}H_{264}N_{34}O_{36}S$ $[M+2H]^{2+}$ 2248.4847 found 2248.4835.

Compound Q33 (4). Compound **13d** (60 mg, 0.007 mmol) was stirred with 50% TFA in $CHCl_3$ (2 mL) at rt around 3h. After that TFA was removed by azeotrope with toluene on rotary evaporator. Then, the crude amine was re-dissolved in dry $CHCl_3$ (2 mL), and commercial 3-(tritylthio)propionic acid (7.5 mg, 0.022 mmol), PyBOP (13 mg, 0.025 mmol) and DIPEA (0.010 mL, 0.072 mmol) were added and the reaction mixture was stirred at 40 °C overnight. Precipitation from MeOH and filtration yielded pure **4** as a light yellow solid (50 mg, 80%). ^{1}H NMR ($CDCl_3$, 300 MHz) δ ppm = 11.03 (1H, s), 10.99 (1H, s), 10.64 (1H, s), 10.60 (1H, s), 10.40 (1H, s), 10.18 (1H, s), 10.12 (1H, s), 10.06 (1H, s), 10.01 (1H, s), 9.94 (1H, s), 9.89 (1H, s), 9.81 (2H, s), 9.34-9.76 (19H, m), 7.85 (1H, dd, *J* = 18.40, 7.36*)*, 7.28-7.77 (33H, m), 7.09-7.22 (5H, m), 6.39-7.09 (74H, m), 6.32 (3H, d, *J* = 6.76*)*, 6.32 (1H, d, *J* = 6.34*)*, 6.16 (1H, s), 6.05 (2H, d, *J* = 9.72*)*, 5.35-5.85 (27H, m), 3.10-3.98 (68H, m), 2.79-2.86 (1H, br), 2.78 (3H, s), 1.85-2.28 (37H, m), 0.92-1.15 (198H, m). HRMS (ESI^+): *m/z* calcd for $C_{486}H_{489}N_{66}O_{68}S$ $[M+3H]^{3+}$ 2791.2241 found 2791.2269.

Compound $Q9_{m=9}$ (5). Compound **13b** (160 mg, 0.068 mmol) was stirred in TFA/$CHCl_3$ (2 mL, 50:50 vol/vol) at 25 °C for 3h. TFA was removed and azeotroped with toluene by rotary evaporation under reduced pressure. Then, the crude amine (154 mg) was redissolved in dry $CHCl_3$ (4 mL), and commercial 11-(tritylthio)undecanoic acid (80 mg, 0.174 mmol), PyBOP (90 mg, 0.174 mmol) and DIPEA (0.050 mL, 0.345 mmol) were added and the reaction mixture was stirred at 40 °C overnight. Solvents were evaporated and the residue was subjected to silica gel column chromatography eluting with ethyl acetate/cyclohexane (25:75 vol/vol) to yield pure **5** as a light yellow solid (168 mg, 91%). ^{1}H NMR ($CDCl_3$, 300 MHz) δ ppm = 11.43 (1H, s), 11.30 (1H, s), 11.15 (1H, s), 10.96 (1H, s), 10. 90 (1H, s), 10.83 (1H,s), 10.78 (1H, s), 10.71 (1H, s), 8.07-8.21 (3H, m), 8.02 (2H, t, *J* = 8.37*)*, 7.58-7.96 (9H, m), 7.55 (1H, d, *J* = 7.17*)*, 7.27-7.43 (12H, m), 6.94-7.25 (14H, m), 6.92 (1H, s), 6.84 (1H, s), 6.66 (1H, s), 6.54 (1H, s), 6.49 (1H, s), 6.43 (1H, d, *J* = 6.58*)*, 6.40 (1H, s), 6.35 (1H, s), 6.17 (1H, s), 6.03 (1H, s), 3.55-4.17 (20H, m), 3.21 (1H, dd, *J* = 15.76, 6.34*)*, 2.99 (3H, s), 2.00-2.58 (13H, m), 0.78-1.51 (70H, m). ^{13}C NMR ($CDCl_3$, 75 MHz): δ= 172.35, 163.56, 162.82, 162.65, 162.60, 162.52, 162.47, 162.22, 162.18, 161.79, 161.28, 161.02, 160.40, 159.22, 159.08, 159.01, 158.51, 149.76, 149.49, 148.96, 148.74, 148.55, 148.28, 145.03, 144.89, 144.10, 138.54, 137.85, 137.49, 137.23, 137.20, 136.85, 135.38, 133.45, 133.31, 133.17, 132.86, 132.51, 132.38, 129.54, 127.71, 127.34, 126.64, 126.42, 125.87, 125.74, 125.52, 125.37,

122.37, 122.14, 122.07, 122.02, 121.87, 121.42, 121.28, 121.23, 120.88, 116.89, 116.67, 116.61, 116.44, 116.22, 116.04, 115.96, 115.60, 115.50, 99.91, 99.25, 98.88, 98.57, 98.50, 98.40, 98.30, 97.50, 97.35, 75.30, 75.20, 75.10, 74.86, 74.78, 74.61, 66.32, 51.85, 38.35, 36.11, 31.93, 29.63, 29.22, 29.12, 29.07, 29.00, 28.91, 28.50, 28.11, 28.01, 27.91, 25.28, 19.54, 19.46, 19.32, 19.24, 19.12. HRMS (ESI^+): *m/z* calcd for $C_{158}H_{167}N_{18}O_{20}S$ $[M+H]^+$ 2669.2358 found 2669.2362.

Compound AQ5 (6). Compound **13a** (50 mg, 0.036 mmol) was stirred in TFA/$CHCl_3$ (1 mL, 50:50 vol/vol) at 25°C for 3h. TFA was removed and azeotroped with toluene by rotary evaporation under reduced pressure. Then crude amine was re dissolved in dry CHCl3 (2 mL), 5-Hexynoic acid (0.013 mL, 0.119 mmol), PyBOP (185 mg, 0.357 mmol) and DIPEA (0.028 mL, 0.195 mmol) were added and the reaction mixture was stirred at 40 °C overnight. The reaction mixture was washed with a saturated aqueous bicarbonate solution and an ammonium chloride solution, successively. The organic phase was dried on Na2SO4, filtered, and the filtrated was evaporated. The residue was subject to preparative GPC to yield pure **6** as a light yellow solid (45 mg, 90%). 1H NMR (CDCl3, 300 MHz) δ ppm = 11.94 (1H, s), 11.83 (1H, s), 11.80 (1H, s), 11.57 (1H, s), 8.56 (2H, d, *J* = 7.68 Hz), 8.06-8.22 (4H, m), 8.01 (2H, d, *J* = 8.02 Hz), 7.86-7.92 (1H, dd, *J* = 8.45, 0.98 Hz), 7.68 (2H, d, *J* = 7.81 Hz), 7.28-7.44 (4H, m), 7.16-7.23 (1H, dd, *J* = 7.21, 1.33 Hz), 6.83 (1H, s), 6.72 (1H, s), 6.70 (1H, d, *J* = 7.04 Hz), 6.58 (1H, s), 4.62 (1H, d, *J* = 6.20 Hz), 4.31-4.45 (2H, m), 4.07-4.25 (3H, m), 3.91-4.00 (3H, m), 3.77-3.88 (3H, m), 3.53-3.65 (1H, dd, *J* = 8.70, 6.38 Hz), 3.20 (3H, s), 2.25-2.60 (5H, m), 1.86-1.94 (2H, m), 1.67-1.83 (3H, m), 1.44-1.53 (2H, m), 1.13-1.17 (30H, m). ^{13}C NMR ($CDCl_3$, 75 MHz): δ= 171.64, 163.98, 163.74, 163.37, 163.21, 163.14, 162.44, 161.92, 161.81, 161.10, 150.75, 150.39, 149.91, 149.04, 145.58, 144.77, 139.20, 138.36, 137.91, 137.40, 135.89, 134.22, 133.66, 127.75, 127.70, 127.27, 127.70, 127.10, 126.83, 125.90, 122.75, 122.58, 122.00, 121.91, 121.62, 121.42, 117.18, 117.13, 116.98, 116.52, 116.29, 116.01, 115.90, 100.34, 99.68, 98.91, 98.00, 97.82, 83.56, 75.66, 75.56, 75.30, 75.25, 75.12, 68.85, 52.19, 38.87, 34.68, 29.80, 28.43, 28.32, 28.24, 23.94, 19.60, 19.53, 19.48, 19.42, 19.35, 17.71. HRMS (ESI^+): *m/z* calcd for $C_{78}H_{83}N_{10}O_{12}$ $[M+H]^+$ 1351.6192 found 1351.6178

Compound AQ9 (7). Compound **13b** (54 mg, 0.023 mmol) was stirred in TFA/$CHCl_3$ (1 mL, 50:50 vol/vol) at 25°C for 3h. TFA was removed and azeotroped with toluene by rotary evaporation under reduced pressure. Then crude amine was re dissolved in dry $CHCl_3$ (2 mL), 5-Hexynoic acid (0.007 mL, 0.066 mmol), PyBOP (34 mg, 0.066 mmol) and DIPEA (0.016 mL, 0.112 mmol) were added and the reaction mixture was stirred at 40 °C overnight. The reaction mixture was washed with a saturated aqueous bicarbonate solution and an ammonium chloride solution, successively. The organic phase was dried on Na_2SO_4, filtered, and the filtrated was evaporated. The residue was subject to preparative GPC to yield pure **7** as a light yellow solid (29 mg, 85%). ^1H NMR (CDCl3, 300 MHz) δ ppm = 11.43 (1H, s), 11.30 (1H, s), 11.16 (1H, s), 10.98 (1H, s), 10.90 (1H, s), 10.83 (1H,s), 10.77 (1H, s), 10.71 (1H, s), 8.09-8.20 (3H, m), 8.02 (2H, t, *J* = 8.63 Hz), 7.86-7.95 (4H, m), 7.62-7.84 (5H, m), 7.56 (1H, d, *J* = 7.72 Hz), 7.29-7.42 (6, m), 7.22 (1H, s), 6.95-7.16 (4H, m), 6.92 (1H, s), 6.85 (1H, s), 6.66 (1H, s), 6.53 (1H, s), 6.49 (1H, s), 6.43 (1H, d, J = 6.54 Hz), 6.40 (1H, s), 6.35 (1H, s), 6.17 (1H, s), 6.03 (1H, s), 3.72-4.18 (17H, m), 3.56-3.69 (3H, m), 3.16-3.27 (1H, dd, *J* = 15.54, 6.66 Hz), 2.99 (3H, s), 2.17-2.56 (9H, m), 1.81 (2H, d, *J* = 6.68 Hz), 1.59-1.67 (3H, m), 1.09-1.38 (56H, m). 13C NMR ($CDCl_3$, 75 MHz): δ= 163.77, 163.04, 162.83, 162.66, 162.43, 162.35, 161.99, 161.45, 161.26, 161.19, 160.58, 159.41, 159.28, 159.21, 158.69, 150.12, 149.68, 149.16, 149.01, 148.95, 148.73, 148.49, 145.09, 144.29, 138.73, 138.02, 137.67, 137.41, 137.39, 136.98, 135.26, 133.62, 133.34, 133.04, 132.73, 132.56, 127.52, 126.82, 126.66, 126.55, 126.05, 125.92, 125.76, 125.51, 122.54, 122.37, 122.23, 122.22, 122.06, 121.60, 121.48, 121.40,

121.12, 117.17, 117.05, 116.84, 116.61, 116.40, 116.05, 115.75, 100.09, 99.44, 99.03, 98.65, 98.62, 97.66, 97.50, 83.42, 75.48, 75.40, 75.28, 75.08, 74.81, 68.76, 52.02, 38.78, 34.58, 29.82, 28.21, 28.16, 23.84, 19.71, 19.61, 19.50, 19.38, 19.29, 17.60. HRMS (ESI+): *m/z* calcd for $C_{134}H_{139}N_{18}O_{20}$ $[M+H]^+$ 2321.0447 found 2321.0426.

Compound AQ17 (8). Compound **13c** (55 mg, 0.012 mmol) was stirred in TFA/$CHCl_3$ (1 mL, 50:50 vol/vol) at 25°C for 3h. TFA was removed and azeotroped with toluene by rotary evaporation under reduced pressure. Then crude amine was re dissolved in dry $CHCl_3$ (2 mL), 5-Hexynoic acid (0.0033 mL, 0.030 mmol), PyBOP (16 mg, 0.035 mmol) and DIPEA (0.009 mL, 0.06 mmol) were added and the reaction mixture was stirred at 40 °C overnight. The reaction mixture was washed with a saturated aqueous bicarbonate solution and an ammonium chloride solution, successively. The organic phase was dried on Na2SO4, filtered, and the filtrated was evaporated. The residue was subject to purified by column with 30% ethyl acetate in cyclo hexane to give **8** as a light yellow colour solid (39 mg, 78%). ^{1}H NMR ($CDCl_3$, 300 MHz) δ ppm = 11.11 (1H, s), 11.05 (1H, s), 10.75 (1H, s), 10.69 (1H, s), 10.49 (1H, s), 9.87-10.30 (11H, m), 7.51-7.99 (21H, m), 7.29 (1H, s), 7.24 (1H, br), 7.20 (2H, t, *J* = 7.75 Hz), 7.08-7.16 (3H, m), 6.65-7.07 (24H, m), 6.39 (2H, d, *J* = 9.74 Hz), 6.28 (1H, d, *J* = 6.77 Hz), 6.21 (1H, s), 6.14 (2H, d, *J* = 7.04 Hz), 5.88 (2H, d, *J* = 10.28 Hz), 5.68-5.79 (8H, m), 3.40-3.98 (36H, m), 2.93-3.04 (1H, dd, J = 15.57, 6.35 Hz), 2.82 (3H, s), 2.05-2.39 (18H, m), 1.67-1.76 (3H, m), 1.42-1.53 (3H, m), 0.98-1.30 (103 H, m). HRMS (ESI+): *m/z* calcd for $C_{246}H_{252}N_{34}O_{36}$ $[M+2H]^{2+}$ 2129.9500 found 2129.9481.

Methyl 8-cyano-4-isobutoxyquinoline-2-carboxylate (10). Methyl 8-cyano-4-oxo-1,4-dihydro-2-quinolinecarboxylate[1] **9** (10 g, 43.85 mmol) was dissolved in dry THF (150 mL). Isobutyl alcohol (6.08 mL, 65.77 mmol) and TPP (17.25 G, 65.77mmol) were added and the mixture was stirred at room temperature for 30 minutes under nitrogen. DIAD (10.36 mL, 52.62 mmol) was added drop wise at 0 °C. Then the reaction mixture was stirred at room temperature overnight. Volatiles were removed by rotatory evaporation. The product was then precipitated from MeOH and DCM to yield a milky white crystalline solid **9** (11.33 g, 91%). m.p.: 179-180 °C. ^{1}H NMR ($CDCl_3$, 300 MHz) δ ppm = 8.49 (1H, dd, *J* = 8.45, 1.29), 8.17 (1H, dd, *J* = 8.15, 1.31), 7.60-7.70 (2H, m), 4.09 (2H, d, *J* = 5.63), 4.08 (3H, s), 2.23-2.37 (1H, m), 1.15 (6H, d, *J* = 6.87). ^{13}C NMR ($CDCl_3$, 75 MHz): δ= 165.69, 163.05, 151.08, 147.66, 136.60, 127.03, 126.49, 122.40, 116.83, 113.54, 102.20, 75.56, 53.29, 28.08, 19.15. HRMS (ESI$^+$): *m/z* calcd for $C_{16}H_{17}N_2O_3$ $[M+H]^+$ 285.1239 found 285.1238.

Methyl 8-((tert-butoxycarbonylamino)methyl)-4-isobutoxyquinoline-2-carboxylate (11). Cyano-quinoline monomer **10** (1 g, 3.5 mmol) was dissolved in mixture of THF/MeOH (50 mL, 30:70 vol/vol) and Raney Ni (around 400 mg) was added. The mixture was stirred at room temperature under H_2 (balloon). After 6h, the starting material had disappeared and Boc_2O (1.15 g, 5.28 mmol) was added and the mixture was stirred at room temperature overnight. The mixture was filtered through celite and solvents were removed by rotary evaporation. Purification by silica gel column chromatography eluting with 10% ethyl acetate/cyclohexane (10:90 vol/vol) yield product **8** as a white solid (0.820 g, 60%). m.p.: 97–98 °C. ^{1}H NMR ($CDCl_3$, 300 MHz) δ ppm = 8.18 (1H, dd, *J* = 8.47, 1.52), 7.74 (1H, d, *J* = 6.72), 7.49-7.56 (2H, m), 6.07 (1H, t, *J* = 5.17), 4.87 (2H, d, *J* = 5.75), 4.05 (3H, s), 4.03 (2H, d, *J* = 6.44 Hz), 2.22-2.35 (1H, m), 1.43 (9H, s), 1.13 (6H, d, *J* = 6.74). ^{13}C NMR ($CDCl_3$, 75 MHz): δ= 166.43, 163.12, 156.24, 148.16, 147.14, 137.77, 129.92, 127.40, 122.60, 121.38, 100.80, 79.18, 75.21, 53.11, 42.54, 28.60, 28.30, 19.36. HRMS (ESI$^+$): *m/z* calcd for $C_{21}H_{29}N_2O_5$ $[M+H]^+$ 389.2076 found 389.2075.

Synthesis of 8-((tert-butoxycarbonylamino)methyl)-4-isobutoxyquinoline-2-carboxylic acid (12). Compound **11** (586 mg, 1.51 mmol) was dissolved THF/MeOH (12 mL, 8:1 vol/vol). NaOH (151 mg, 3.775 mmol) was dissolved in 1 mL of water and then added to the reaction mixture at 0 °C, which was then stirred at room temperature for 3 hours. Complete conversion was confirmed by TLC and sodium carboxylate salt was neutralized by adding a 5% citric acid aqueous solution. Volatiles were removed under reduced pressure, and the residue dissolved in $CHCl_3$ and washed with water. The organic phase was dried over Na_2SO_4, filtered, and the filtrate was removed by rotary evaporation to yield **9** as a white solid (542 mg, 96%). m.p.: 176-177 °C. ^{1}H NMR ($CDCl_3$, 300 MHz) δ ppm = 8.24 (1H, dd, *J* = 8.60, 1.21*)*, 7.67 (1H, d, *J* = 6.58*)*, 7.65 (1H, s), 7.55 (1H, t, *J* = 7.49*)*, 4.92 (2H, s), 4.07 (2H, d, *J* = 6.49 Hz), 2.22-2.35 (1H, m), 1.44 (9H, s), 1.13 (6H, d, *J* = 6.70*)*. ^{13}C NMR ($CDCl_3$, 75 MHz): δ= 165.65, 164.02, 156.31, 147.03, 145.42, 137.32, 130.64, 127.42, 122.95, 122.29, 99.29, 80.06, 75.57, 41.91, 28.57, 28.22, 19.34. HRMS (ESI$^+$): *m/z* calcd for $C_{20}H_{27}N_2O_5$ $[M+H]^+$ 375.1920 found 375.1919.

Compound 13a. Compound **12** (186 mg, 0.499 mmol) was dissolved in anhydrous $CHCl_3$ (2 mL). 1-Chloro-*N*,*N*,2-trimethyl-1-propenylamine (0.132 mL, 0.998 mmol) was added and the reaction mixture was stirred at room temperature for 2 h. The solvent was evaporated and the residue was dried *in vacuo* to remove any remaining chloro-enamine. The residue was then re-dissolved in dry $CHCl_3$ (3 mL) under nitrogen. In another round bottom flask, the amine terminated tetrameric quinolinecarboxamide methyl ester[2] (200 mg, 0.199 mmol) was also dissolved in dry $CHCl_3$ (2 mL) under nitrogen. DIPEA (0.146 mL. 0.995 mmol) and then the acid chloride solution was added drop wise to the amine. The mixture was stirred at room temperature overnight. The reaction mixture was washed with saturated aqueous $NaHCO_3$, aqueous NH_4Cl, and brine. The organic layer was dried under Na_2SO_4, filtered and the residue was evaporated. Purification of the residue by GPC yielded **13a** (225 mg, 83%) as a light yellow solid. ^{1}H NMR ($CDCl_3$, 300 MHz) δ ppm = 11.93 (1H, s), 11.81 (2H, s), 11.57 (1H, s), 8.50-8.62 (2H, m), 8.05-8.24 (4H, m), 8.01 (2H, d, *J* = 8.13*)*, 7.85 (1H, d, *J* = 8.11Hz), 7.67 (2H, t, *J* = 7.77*)*, 7.39 (2H, d, *J* = 16.76*)*, 7.33 (2H, t, *J* = 8.17*)*, 7.20 (1H, t, *J* = 7.36*)*, 6.83 (1H, s), 6.74 (2H, s), 6.57 (1H, s), 4.32-4.46 (2H, m), 4.09-4.26 (2H, m), 3.89-4.08 (4H, m), 3.72-3.89 (4H, m), 3.26 (1H, dd, *J* = 15.08, 6.43*)*, 3.20 (3H, s), 2.24-2.58 (5H, m), 1.04-1.44 (39H, m). ^{13}C NMR ($CDCl_3$, 75 MHz): δ= 164.04, 163.77, 163.51, 163.28, 163.14, 162.47, 161.97, 161.87, 161.23, 161.16, 155.49, 150.92, 150.49, 149.74, 149.08, 145.63, 144.92, 139.27, 138.45, 138.01, 137.40, 136.51, 134.27, 133.88, 133.78, 133.56, 127.77, 127.35, 127.15, 127.02, 125.93, 122.83, 122.62, 122.08, 121.96, 121.58, 121.40, 117.23, 117.06, 116.56, 116.30, 116.14, 116.10, 115.82, 100.38, 99.70, 99.13, 98.04, 97.74, 78.87, 75.68, 75.53, 75.44, 75.33, 75.14, 52.24, 40.28, 28.43, 28.38, 28.29, 19.61, 19.55, 19.45, 19.38. HRMS (ESI$^+$): *m/z* calcd for $C_{77}H_{85}N_{10}O_{13}$ $[M+H]^+$ 1357.6298 found 1357.6301.

Compound 13b. Compound **12** (25 mg, 0.066 mmol) was dissolved in anhydrous $CHCl_3$ (1 mL). 1-Chloro-*N*,*N*,2-trimethyl-1-propenylamine (0.017 mL, 0.133 mmol) was added and the reaction mixture was stirred at room temperature for 2 h. The solvent was evaporated and the residue was dried *in vacuo* to remove any remaining chloro-enamine. The residue was then re-dissolved in dry $CHCl_3$ (3 mL) under nitrogen. In another round bottom flask, the amine terminated octameric quinolinecarboxamide methyl ester[3] (50 mg, 0.025 mmol) was also dissolved in dry $CHCl_3$ (1 mL) under nitrogen. DIPEA (0.018 mL. 0.126 mmol) and then the acid chloride solution was added drop wise to the amine. The mixture was stirred at room temperature overnight. The reaction mixture was washed with saturated aqueous $NaHCO_3$, aqueous NH_4Cl, and brine. The organic layer was dried under Na_2SO_4, filtered and the residue was evaporated. Purification of the residue by silica gel column chromatography, eluting with ethyl

acetate/cyclohexane (25:75 vol/vol), yielded **13b** as a light yellow solid (56 mg, 95 %). ^1H NMR ($CDCl_3$, 300 MHz) δ ppm = 11.43 (1H, s), 11.30 (1H, s), 11.14 (1H, s), 10.98 (1H, s), 10.90 (1H, s), 10.83 (1H,s), 10.79 (1H, s), 10.70 (1H, s), 8.17 (2H, d, *J* = 8.02*)*, 8.10 (1H, d, *J* = 7.43*)*, 8.01 (2H, t, *J* = 9.21 Hz), 7.60-7.95 (9H, m), 7.55 (1H, d, *J* = 7.22*)*, 7.28-7.42 (5, m), 7.22 (1H, s), 6.95-7.16 (4H, m), 6.93 (1H, s), 6.84 (1H, s), 6.66 (1H, s), 6.51 (2H, d, *J* = 12.58*)*, 6.37 (2H, d, *J* = 13.72*)*, 6.17 (1H, s), 6.01 (1H, s), 3.73-4.16 (15H, m), 3.55-3.69 (4H, m), 3.34 (1H, t, *J* = 5.67*)*, 2.99 (3H, s), 2.89 (1H, dd, *J* = 15.74, 6.48*)*, 2.16-2.54 (9H, m), 1.07-1.39 (54H, m), 1.01 (9H, s). ^{13}C NMR ($CDCl_3$, 75 MHz): δ= 163.75, 162.97, 162.79, 162.62, 162.40, 162.29, 161.96, 161.36, 161.31, 161.16, 160.56, 159.37, 159.25, 158.78, 155.27, 150.12, 149.77, 149.12, 149.09, 148.90, 148.70, 148.46, 145.07, 144.36, 138.71, 138.01, 137.67, 137.48, 137.41, 136.98, 136.10, 133.63, 133.43, 133.32, 133.04, 132.78, 132.56, 127.50, 127.09, 126.90, 126.81, 126.62, 126.03, 125.86, 125.73, 125.69, 125.52, 122.53, 122.31, 122.28, 122.22, 122.04, 121.93, 121.58, 121.45, 121.39, 121.07, 117.07, 117.03, 116.80, 116.58, 116.33, 116.18, 116.11, 115.93, 115.78, 115.72, 115.66, 100.06, 99.43, 99.02, 98.61, 98.56, 97.63, 97.48, 78.65, 75.43, 75.35, 75.25, 75.19, 75.09, 75.03, 74.78, 52.02, 39.92, 28.26, 28.16, 28.07, 19.68, 19.59, 19.50, 19.37, 19.27. HRMS (ESI$^+$): *m/z* calcd for $C_{133}H_{141}N_{18}O_{21}$ $[M+H]^+$ 2327.0552 found 2327.0517.

Compound 13c. Compound **12** (53 mg, 0.141 mmol) was dissolved in anhydrous $CHCl_3$ (1 mL). 1-Chloro-*N*,*N*,2-trimethyl-1-propenylamine (0.037 mL, 0.283 mmol) was added and the reaction mixture was stirred at room temperature for 2 h. The solvent was evaporated and the residue was dried *in vacuo* to remove any remaining chloro-enamine. The residue was then re-dissolved in dry $CHCl_3$ (0.5 mL) under nitrogen. In another round bottom flask, the amine terminated hexadecameric quinolinecarboxamide methyl ester[2] (225 mg, 0.057 mmol) was also dissolved in dry $CHCl_3$ (2 mL) under nitrogen. DIPEA (0.032 mL. 0.287 mmol) and then the acid chloride solution was added drop wise to the amine. The mixture was stirred at room temperature overnight. The reaction mixture was washed with saturated aqueous $NaHCO_3$, aqueous NH_4Cl, and brine. The organic layer was dried under Na_2SO_4, filtered and evaporated. Precipitation from MeOH afforded **13c** (210 mg, 86%) as a light yellow solid. ^1H NMR ($CDCl_3$, 300 MHz) δ ppm = 11.11 (1H, s), 11.05 (1H, s), 10.73 (1H, s), 10.69 (1H, s), 10.48 (1H, s), 10.28 (1H, s), 10.23 (1H, s), 10.19 (1H, s), 10.12 (1H, s), 10.09 (1H, s), 10.03 (1H, s), 10.00 (1H, s), 9.99 (2H, d = 3.47*)*, 9.95 (1H, s), 9.91 (2H, d, *J* = 2.77*)*, 7.51-7.98 (21H, m), 7.29 (1H, s), 7.23 (1H, s), 7.20 (1H, s), 7.08-7.19 (3H, m), 6.66-7.07 (25H, m), 6.39 (3H, d, *J* = 9.61*)*, 6.21 (1H, s), 6.14 (2H, d, *J* = 7.35*)*, 5.87 (2H, d, *J* = 10.74*)*, 5.67-5.80 (8H, m), 3.84-3.98 (4H, m), 3.33-3.80 (31H, m), 3.17 (1H, t, *J* = 5.84*)*, 2.82 (3H, s), 2.65 (1H, dd, *J* = 15.76, 7.00*)*, 2.05-2.37 (17H, m), 0.99-1.26 (102H, m), 0.92 (9H, s). HRMS (ESI$^+$): *m/z* calcd for $C_{245}H_{254}N_{34}O_{37}$ $[M+2H]^{2+}$ 2132.9553 found 2132.9534.

Compound 13d. Compound **12** (12 mg, 0.0315) was dissolved in anhydrous $CHCl_3$ (0.5 mL). 1-Chloro-*N*,*N*,2-trimethyl-1-propenylamine (0.010 mL, 0.080 mmol) was added and the reaction mixture was stirred at room temperature for 2 h. The solvent was evaporated and the residue was dried *in vacuo* to remove any remaining chloro-enamine. The residue was then re-dissolved in dry $CHCl_3$ (0.5 mL) under nitrogen. In another round bottom flask, the amine terminated 32meric quinolinecarboxamide methyl ester[2] (49 mg, 0.0062 mmol) was also dissolved in dry $CHCl_3$ (1 mL) under nitrogen. DIPEA (0.005 mL. 0.031 mmol) and then the acid chloride solution was added dropwise to the amine. The mixture was stirred at room temperature overnight. The reaction mixture was washed with saturated aqueous $NaHCO_3$, aqueous NH_4Cl, and brine. The organic layer was dried under Na_2SO_4, filtered and evaporated. Precipitation from MeOH afforded **13d** (40 mg, 80%) as a light yellow solid. ^1H NMR ($CDCl_3$, 300 MHz) δ ppm = 11.03 (1H, s), 10.99 (1H, s), 10.65 (1H, s), 10.63 (1H, s), 10.40 (1H, s), 9.39-10.22 (27H, m),

7.28-7.93 (38H, m), 6.39-7.22 (62H, m), 6.33 (3H, d, *J* = 6.86*)*, 6.16 (1H, s), 6.06 (2H, d, *J* = 9.94*)*, 5.77 (2H, d, *J* = 15.77*)*, 5.54-5.70 (7H, m), 5.34-5.54 (17H, m), 3.04-3.92 (68H, m), 3.03-3.91 (77H, m), 2.77 (3H, s), 2.60 (1H, dd, *J* = 14.81, 6.35*)*, 1.92-2.30 (33H, m), 0.70-1.35 (207H, m). HRMS (ESI^+): *m/z* calcd for $C_{469}H_{479}N_{66}O_{69}$ $[M+3H]^{3+}$ 2714.5390 found 2714.5368.

UV-visible and fluorescence spectroscopy. Measurements were carried out in **Q5-Q33** foldamer solutions (ca 5 μM, chloroform). UV-visible spectra were acquired at room temperature in a Varian Cary 5000 UV-visible spectrophotometer using a quartz cell of 1 cm optical path. Fluorescence emission spectra were acquired in a Horiba Jobin-Yvon Fluorolog 3 spectrophotometer at an excitation wavelength of 360 nm.

Monolayer preparation. All glassware employed for monolayer preparation was cleaned with hot piranha, (conc. H_2SO_4-H_2O_2 3:1, **warning**: piranha should be handled with caution; it reacts strongly in contact with organic compounds), thoroughly rinsed with milliQ water and dried in an oven. Preparation of monolayers by *in situ* trityl deprotection was carried out as reported with some modifications.[4] Briefly, the corresponding trityl-protected derivative was dissolved in an excess of trifluoroacetic acid (TFA) (1-4 mg in 80 μL TFA) to give a deep-yellow solution, and triethylsilane (5-10 μL) was added until the solution became faintly yellow, then the solution was incubated for 15 min. The solution was evaporated under reduced pressure and the residue was dissolved in degassed EtOH-$CHCl_3$ 1:1 (**Q5**, **Q9** and **Q17**) or EtOH-$CHCl_3$ 1:2 (**Q33**) to a concentration of 1 mM (**Q5**, **Q9** and **Q17**) or 0.7 mM (**Q33**). Gold substrates (300 nm Au on mica, Georg Albert PVD – Beschichtungen, Germany) were immersed in the solution and sealed after backfilling with Ar. The substrates were incubated 48 h at 50 ºC. Afterwards, the substrates were rinsed with $CHCl_3$ (ca 3 mL), EtOH (ca 3 mL), and dried with a steam of dry Ar.

Immobilization of foldamers on SiO_2: For characterization proposes, native SiO_2 (ca 2-3 nm thick) on Si wafers were used, while 200 nm SiO_2 on Si wafers were used for electrode fabrication. Wafers were cleaned with piranha solution (see warning above) for 1 h., then thoroughly rinsed with milliQ water, blow-dried with a steam of Ar and further dried in an oven (100 ºC) for 5-10 min. Substrates were incubated in a solution of 11-bromoundecyl trichlorosilane in freshly distilled toluene (0.1 % v/v) for 1 h., then rinsed with toluene, briefly sonicated in toluene, rinsed with EtOH and dried with a steam of Ar. Substrates were immersed in a saturated solution of NaN_3 in DMF for 2 days at 70 ºC, then rinsed and sonicated with milliQ water, rinsed with EtOH and blow dried with a steam of Ar. Foldamers were clicked by immersion of the substrates in a solution of alkyne-functionalized foldamers (0.5 mM), Tris[(1-benzyl-1*H*-1,2,3-triazol-4-yl)methyl]amine (TBTA, 0.5 mM) and ascorbic acid (3 mM) in DMF (0.5 mL) for 3 h at 50 ºC. Substrates were rinsed with DMF, DCM, EtOH and water, then dried by rinsing with EtOH and blow-drying with a steam of Ar.

Substrates for PM-IRRAS analysis were prepared by carrying out the same deprotection protocol and diluting the derivative to 0.1 mM solutions. Prior to immersion, gold square substrates of ca 2.5 cm sides (200 nm Au on glass, Ssens, Netherlands for monolayers on gold, and SiO_2/Au for monolayers in silicon dioxide) were cleaned in piranha solution (conc. H_2SO_4-H_2O_2 3:1, **warning**: piranha should be handled with caution; it reacts strongly in contact with organic compounds), thoroughly rinsed with milliQ water and immersed in EtOH for 20 min. After incubation in the foldamer solution (48 h at 50 ºC.), substrates were briefly sonicated in $CHCl_3$ (30 s), rinsed with $CHCl_3$, EtOH, and dried with a steam of dry Ar.

Ellipsometry. Ellipsometry measurements were carried out in an EP3 null-ellipsometer (Nanofilm, Germany) and analyzed with the software (EP4Model 1.0.1) provided with the instrument. The instrument was used in total internal reflection mode and both the intensity and the phase changes of the reflected light were monitored and converted into the ellipsometric angles Ψ and Δ. A wavelength range from 370 to 720 nm was scanned at a constant angle of incidence of 70º. Film thickness was determined by fitting the ellipsometric angles to a built-up model of gold-organic-air. The optical parameters of the gold layer were obtained experimentally by measuring a bare gold substrate. Monolayers prepared on top of SiO_2 were fitted with a Si-SiO_2-organic-air model, where the thickness of the SiO_2 layers was obtained experimentally from non functionalized surfaces. In all the cases, the organic layer was modeled assuming a refractive index of 1.5. Results are expressed as the average and standard deviation of three independent monolayer preparations.

Contact angle. Static contact angle were measured in a Drop Shape Analyzer - DSA 100 (Krüss, Hamburg, Germany) by deposition of a 3 µL milliQ water sessile drop on top of the surface. Profiles were captured and analyzed with the software provided with the instrument. Results are expressed as the average and standard deviation of three independent monolayer preparations.

Electrochemistry. Electrochemical measurements were carried out in an Autolab potentiostat (Metrohm, France) using 1.6 mm diameter (BASi) gold disk electrodes. Before monolayer functionalization, electrodes were polished with 300 nm alumina slurry (Metrohm), then sonicated in EtOH (5 min) and milliQ water (5 min). Electrodes were electrochemically cleaned in a H_2SO_4 0.5 M solution by applying an oxidizing potential (2 V, 5 sec) and a reductive potential (-0.35 V, 10 s), and followed by continuous scanning from -0.25 to 1.55 V for a minimum of 40 cycles at 0.1 V s^{-1} and until a stable signal was achieved. Right after the electrode was rinsed with milliQ water and EtOH, then dried with a steam of Ar and immediately immersed in the thiol solution for 12-24 h at room temperature. Electrochemical measurements were carried out using a three electrode system: the working electrode, an Ag/AgCl reference electrode and a coiled platinum wire as counter electrode. Passivation experiments were carried out in the presence of $Fe(CN)_6^{3/4-}$ 1 mM in KCl 0.1 M at a scan rate of 0.1 V s^{-1}. Double layer capacitance (C_{dl}) was calculated from non-faradaic currents in cyclic voltammograms (KCl 0.1 M) according to $C_{dl} = (I_a + I_c)/2vA$ where I_a and I_c are designating the anodic and catodic current intensity respectively, v the scan rate and A the area of the electrode.

PM-IRRAS. PM-IRRAS spectra were recorded using a ThermoNicolet Nexus 670 FTIR spectrometer at a resolution of 4 cm^{-1} by co-adding several blocks of 1500 scans (30 min acquisition time). Generally, eight blocks (4 h acquisition time) were necessary to obtain PM-IRRAS spectra of SAMs with good signal-to-noise ratios. Experiments were performed at an incidence angle of 75º by using an external homemade goniometer reflection attachment and adding a ZnSe photoelastic modulator (PEM, Hinds Instruments, type III) after the polarizer.[5,6] PM-IRRAS spectra are presented in terms of the IRRAS unit (i.e. 1-[Rp(d)/Rp(0)], where Rp(d) and Rp(0) stand for the p-polarized reflectance of the film/substrate and bare substrate systems, respectively) by using a calibration procedure.[7,6]

Au electrode deposition on SiO_2/Si: PDMS stamps (cast on flat Si wafers) were cut with a sharp razor and briefly cured in an UV/Ozone chamber (10 min), then gently deposited on top of foldamer monolayers to protect about half of the surface. Au was thermaly evaporated (50 nm) in a vacuum chamber glovebox (MBRAUN) and monitorized with a quartz microbalance. After electrode deposition

the stamp was peeled-off and the surface rinsed with EtOH, then blow dried with a steam of Ar. Samples were stored under inert atmosphere prior to measurements.

AFM imaging. Monolayer imaging in air was carried out in a Dimension FastScan AFM (Bruker) in tapping mode using FastScan-A tips (Bruker, nominal parameters: radius, 5 nm; spring constant, 18 N/m, resonant frequency, 1400 kHz)

Conducting AFM. *I-V* curves were collected in air in a Dimension Icon AFM (Bruker) in PeakForce TUNA mode using PFTUNA tips (Bruker, platinum/iridium tip, nominal parameters: radius, 25 nm; spring constant, 0.4 N/m). Substrates were grounded with silver paste. Prior to measurements, the tip spring constant was calibrated in order to calculate the applied force. Junctions were established at gentle tip pressures using the "point & shoot" mode. Nine points were distributed in ca 2500 nm^2 and measured by triplicate in each point, scanning the bias forward and backwards. Data was collected from several different areas on the sample and at different tip pressures. The same tip was employed to measure different monolayers to avoid differences arising from variable tip diameter. Comparative experiments were repeated with different tips and substrates.

Data analysis. Data analysis of *I-V* curves was carried out in R[8] with the help of R packages for data analysis/visualization.[9,10,11] Prior analysis, *I-V* curves arising for short circuit -showing high mobility at ca. 0 V and very high conductivity, as well as curves clearly showing hints of a defective tip, were discarded. Resistances were obtained from biases in the range of -0.3 to 0.3 V. Distributions were characterized by their median and their adjusted median absolute deviation, according the proposed statistical analysis reported by Reus *et al.*[12]

Computational Details. Density functional theory (DFT) calculations were performed with Gaussian09,[13] utilizing the 6-31G(d) basis set and the well balanced PBE0 exchange correlation functional[14] plus Grimme's D3 long range corrections[15] to account for intrachain van der Waals and dispersion interactions along the foldamer. Molecular structures were constructed by increasing the number n of monomeric units (n=1-8) and optimized at the PBE0-D3BJ/6-31G(d) level in the neutral, positively and negatively charged states. The reorganization energies for electron (λ_e) and holes (λ_h), were calculated with the well-known four points formula.[16] These parameters are key in charge transport processes, not only because they strongly condition the mobility values in Marcus hopping transport theory, but also because they provide a direct measurement of the electron-phonon coupling strength and hence of the likeliness of a tunneling (weak coupling) or hopping (strong electron phonon coupling) charge transport mechanism. Hole and electron transfer integrals *J* were computed at PBE0/6-31G(d) level with Orca 3.03,[17] adopting the localized monomer orbital approximation and following the method of Valeev and coworkers.[18] In practice, for holes (electrons) the transfer integral is calculated as the electronic coupling between the orthonormalized HOMOs (LUMOs) of two quinoline units at a time. Calculations were performed starting from experimental crystal structures,[2] replacing the external terbutyl groups by hydrogen atoms. The quinoline-carbonyl bonds along the helix were cleaved and the valence of pending carbon atoms completed by hydrogens. The transfer integrals for holes and electrons were then computed between all the possible quinoline pairs.

Kinetic Monte Carlo (KMC) simulations were run assuming holes to be localized on single quinoline units, utilizing the experimental 48-mer[2] geometry for determining the position in space of the units, and the Marcus equation for computing the hopping rate from unit *i* to unit *j*:

$$k_{ij} = \frac{2\pi}{\hbar} J_{ij}^2 \frac{1}{\sqrt{4\pi\lambda_h k_B T}} exp\left[\frac{-\left(\lambda_h + \varepsilon_j - \varepsilon_i - eE \cdot \vec{r}_{ji}\right)^2}{4\lambda_h k_B T} \right]$$

where J_{ij} is the transfer integral, ε_j and ε_i are the energies of the HOMO levels, e is the electron charge, $\vec{r}_{ji}$ is the inter-unit distance vector and E is the applied electric field, in this case parallel to the helix axis. The projection of $\vec{r}_{ji}$ along the helix axis amounts to about 1.4 Å times the number of quinoline units separating *j* from *i*. In the KMC simulations, the electric field was varied between 0.005 to 1 V/Å, λ_h was set to 250 meV and *T* to 300 K. At time zero, the hole was placed at the first unit of the helix, then its position was evolved by i) computing the rates of hopping to the neighbors with Marcus' equation, ii) generating a random transfer time $t_{ij} = -k_{ij}^{-1} \ln x$ for each of them, where *x* is a uniformly distributed real random number in the]0:1] interval, and iii) hopping to the neighbor corresponding to the shortest transfer time.[19] The time is advanced accordingly, and the procedure is iterated until the charge reaches the final unit of the helix. Two different series of simulations were performed to investigate the effect of fluctuations in transfer integrals and site energies. In the first, J_{ij}were kept fixed to the values reported in Table S2 for the n=48 helix, and ε_j and ε_i were set to zero. In the second series, a random Gaussian disorder was added to those quantities, adopting the values obtained from the calculation of the transfer integrals for the n=48 helix (σ_J=17 meV, σ_ε=127 meV), and the values of J_{ij}, ε_j, ε_i were re-generated at each step of the KMC algorithm. Presented results were averaged over 100000 independent simulations, with average times calculated as the exponential of the mean of time logarithms, as suggested by Bobbert and coworkers.[20]

Table S1: Electronic properties of foldamers composed by an increasing number of repeating units n. All energies are expressed in eV. λ_{max} is the position of the most intense peak in the 300-450 nm region of the calculated absorption spectrum. $\Delta E_{S0\rightarrow S1}$ is the vertical optical band gap.

n	HOMO	LUMO	IP	EA	λ_h	λ_e	$\Delta E_{S0\rightarrow S1}$	λ_{max}
1	-5.91	-1.16	7.28	-0.20	0.43	0.37	4.06	305
2	-5.82	-1.69	6.87	0.70	0.26	0.41	3.46	337
3	-5.71	-1.82	6.67	0.92	0.39	0.34	3.25	343
4	-5.62	-1.83	6.50	0.99	0.25	0.36	3.13	342
5	-5.59	-1.79	6.42	1.01	0.20	0.34	3.13	346
6	-5.54	-1.78	6.33	1.05	0.23	0.32	3.11	346
7	-5.49	-1.76	-	-	-	-	3.08	346
8	-5.47	-1.74	-	-	-	-	3.07	-

Table S2: Hole and electron transfer integrals between quinoline repeating units along the foldamer helix, calculated at the PBE0/6-31G(d) level considering orbitals localized on single units. Calculations were performed using experimental structures of helices with 9 and 48 units. See Figure 4 for the definition of the neighbors.

units	J_h (meV)	J_e (meV)
i,*i*+1 (n=9)	82 ± 21	208 ± 250
i,*i*+2	18 ± 11	25 ± 13
i,*i*+3	41 ± 23	20 ± 18
i,*i*+1 (n=48)	92 ± 14	220 ± 59
i,*i*+2	27 ± 19	32 ± 15
i,*i*+3	48 ± 17	20 ± 9

Theoretical absorption spectra were calculated at TDDFT/PBE0/6-31G(d) level at the PBE0-D3BJ/6-31G(d) optimized geometry, using 8 states per quinoline unit, and enlarging the absorption peaks using Gaussian functions with a full width at half maximum of 0.3 eV. The calculated spectra are shown in Figure S1, while the absorption spectrum of the foldamer with four units**,** alongside the calculated oscillator strengths, is displayed in Figure S2. The detailed analysis of the theoretical data reveals that the two main absorption bands originate from the superposition of electronic transitions between the ground state and several excited states. Among these electronic transitions, those associated to the largest oscillator strengths were further characterized by analyzing the variation of the total electron density upon the electron excitation. Density maps reported in Figure S3 evidence that, for all states, intramolecular charge transfer occur over several monomeric units.

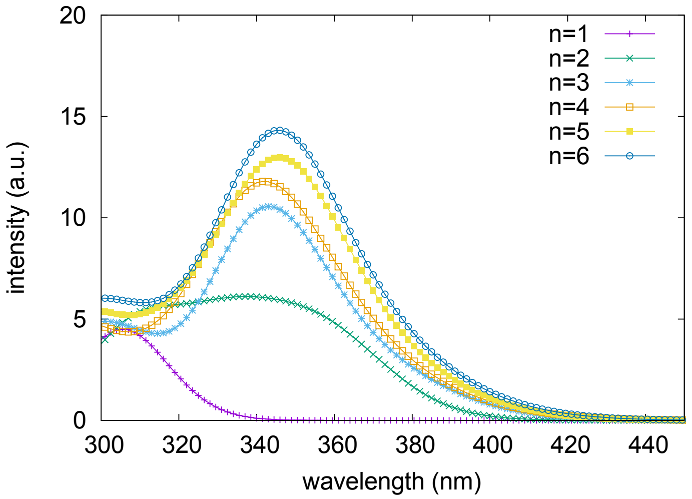

Figure S1: Calculated absorption spectra for foldamers composed by one to six quinoline units at the PBE0/6-31G(d)-D3BJ optimized geometry.

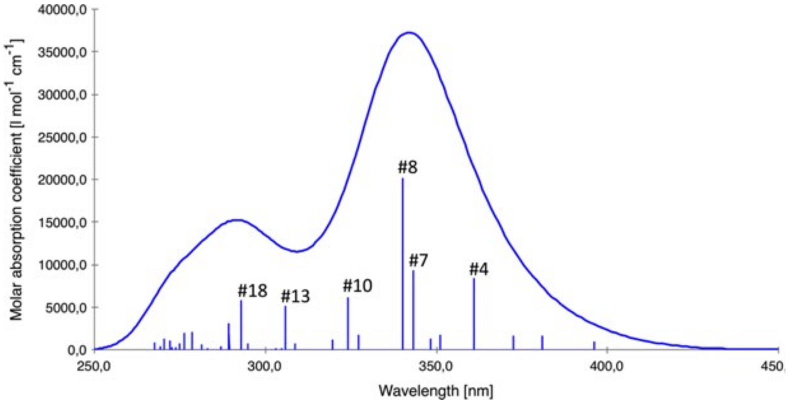

Figure S2: UV/visible spectrum of the foldamer with four units, as calculated at the TDDFT/PBE0/6-31G(d) level. Impulses are relative oscillator strengths.

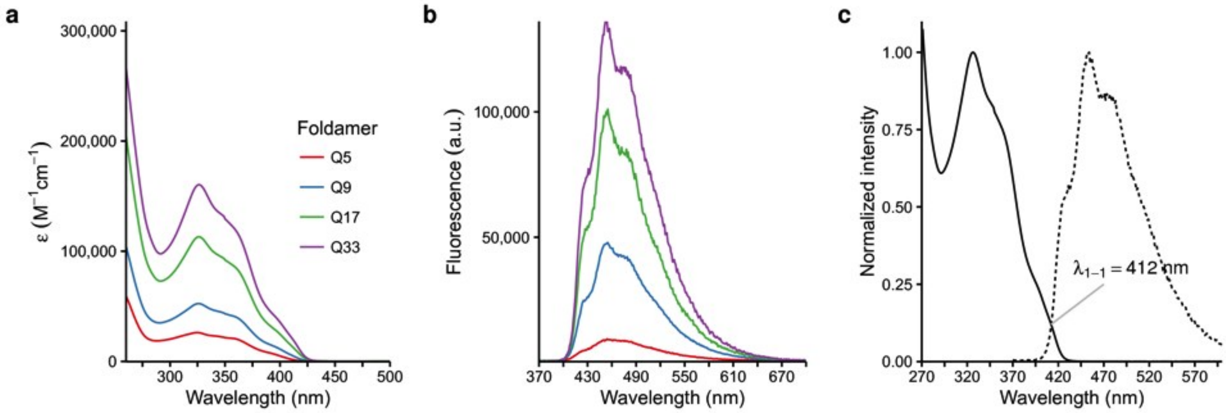

Figure S3. a) UV-visible spectra and b) fluorescence spectra (λ_{ex} 360 nm) of solutions of foldamers **Q5**-**Q33** in $CHCl_3$ (5 µM); c) Normalized absorption (plain lines) and emission (dashed lines) of a solution of foldamer **Q33** in $CHCl_3$ (5 µM).

Table S3: Electronic properties of foldamers **Q5**-**Q33** determined by UV-visible spectroscopy and fluorescence. λ_{1-1} and ΔE_{1-1} were determined from the crossing point of UV-visible and fluorescence normalized spectra (Figure S4c).

Foldamer	$\lambda_{max,abs}$ (nm)	$\lambda_{offset,abs}$ (nm)	λ_{1-1} (nm)	ΔE_{1-1} (eV)
**Q5**	324	418	408	3.04
**Q9**	326	422	410	3.02
**Q17**	326	424	412	3.01
**Q33**	326	426	412	3.01

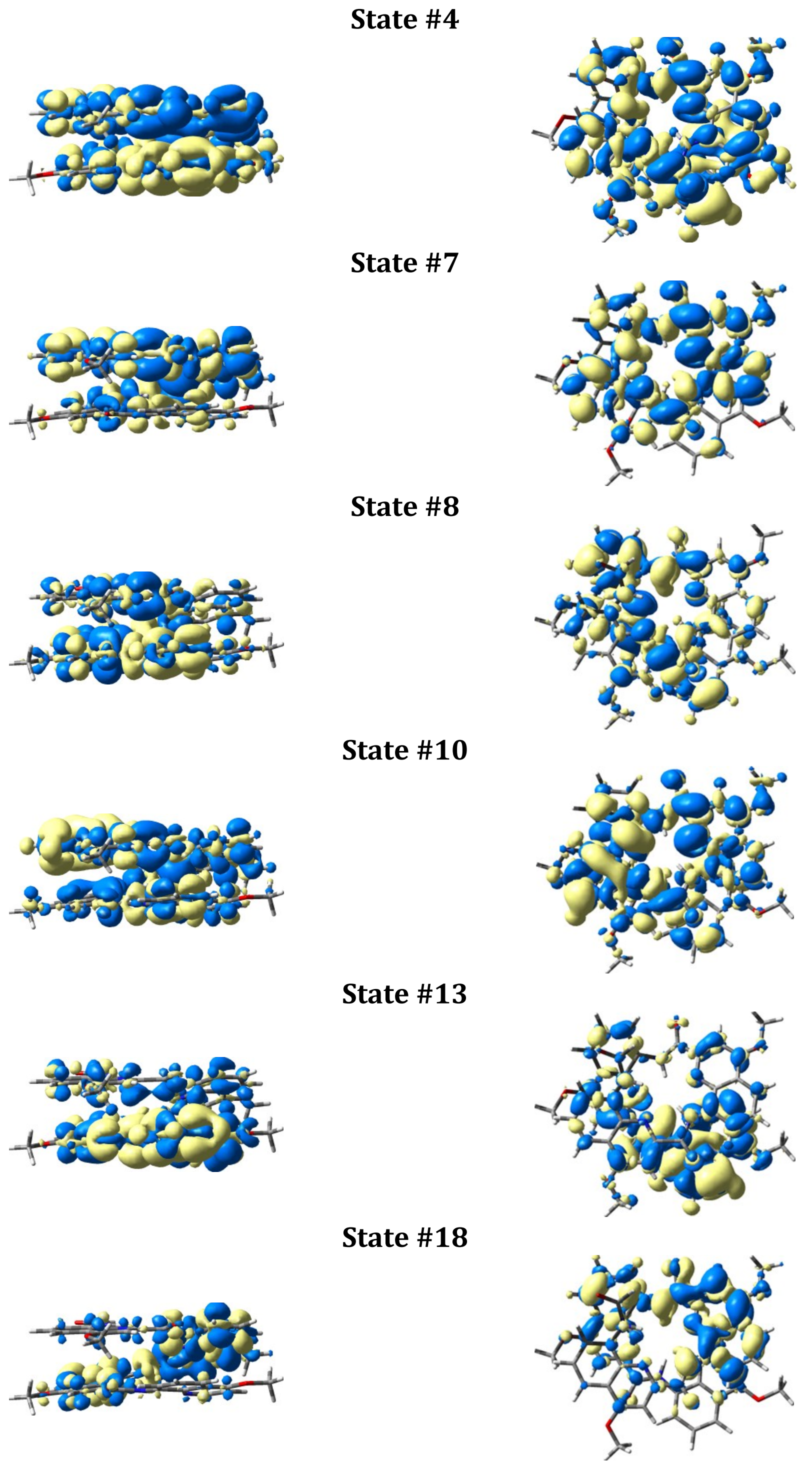

Figure S4: Electron density difference maps between the ground state and the main excited states for the foldamer with four units, as calculated at the TDDFT/PBE0/6-31G(d) level and plotted using isovalues of 0.0004 a.u.. Blue and yellows lobes are associated to positive and negative differences, respectively. The state labels correspond to those given in Figure SB.

Characterization of the monolayers

Monolayers on gold of Q5-Q33 and $Q9_{m=9}$

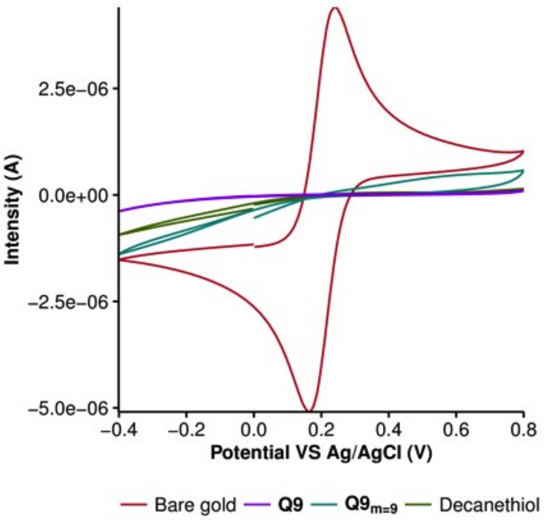

Figure S5. Cyclic voltammogram on gold electrodes in the presence of 1 mM $K_3Fe(CN)_6^{3-}$ in 0.1 M KCl at 0.1 V/s. Measurements were carried out in an unmodified electrode (bare gold) or modified with monolayers of **Q9**, **$Q9_{m=9}$** or decanethiol. A dramatic reduction of the faradaic signal of $Fe(CN)_6^{3-}$ indicates kinetically limited electron transfer.[21] Because electron transfer processes are very sensitive to the presence of surface defects, this indicates that the self-assembly is good enough to minimize the presence of pinholes on the surface.

Table S4. Characterization of monolayers on gold self-assembled from compounds **Q9** and **$Q9_{m=9}$**, incubated at room temperature overnight.[a] Determined by ellipsometry; [b]Determined by cyclic voltammetry in the presence of 0.1 M KCl. Results represent the average of a duplicated experiment ± standard deviation. Notice that water contact angle and film thickness for **Q9** are higher when incubating 48 h at 50 ºC (see Figure 2). This suggests that the packing is improved at higher temperatures and longer incubation times.

Compound	Film thickness (nm)[a]	Contact angle (deg)	C_m (µF cm^{-2})[b]
**Q9**	0.9 ± 0.2	61.0 ± 0.6	3.9 ± 2.3
**$Q9_{m=9}$**	1.6 ± 0.1	63.8 ± 0.7	2.3 ± 2.0

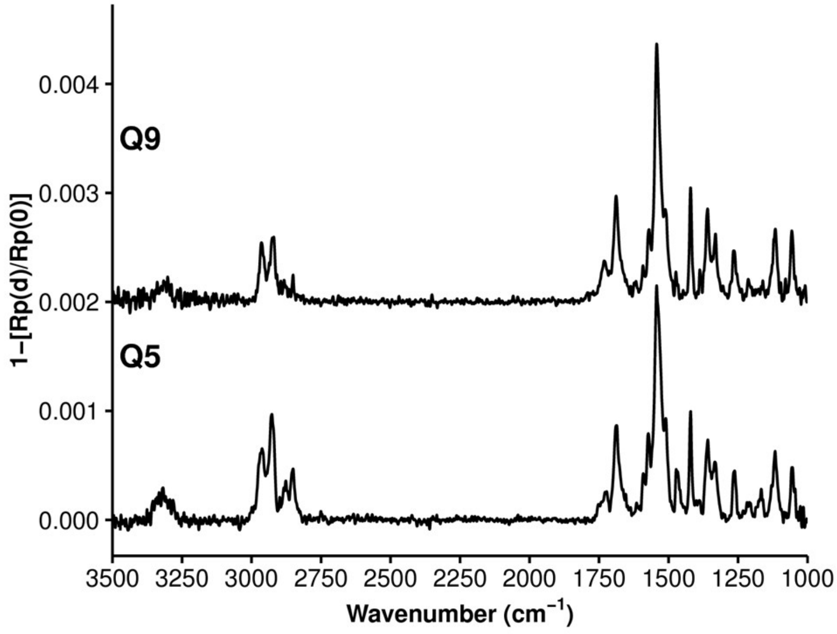

Figure S6. PM-IRRAS spectra of monolayers of **Q5** and **Q9** on gold prepared at 0.1 mM concentrations.

Table S5. Energy, assignation and origin of major bands on Figure S6.

Wavenumber (cm^{-1})	Assignment	Origin
3315	ν N-H amide	Amide A
2965	ν_a CH_3	Isobutyl
2927	ν_a CH_2	Isobutyl
2875	ν_s CH_3	Isobutyl
2854	ν_s CH_2	Isobutyl
1730	ν CO	COOMe
1688	ν CO	Amide I
1616, 1590, 1571	ν C=C	Quinolone
1543	δ N-H	Amide II
1509	ν C=C	Quinoline
1468	δ CH_2	Isobutyl
1420	δ CH_3	Isobutyl
1386, 1359, 1332	ν C-C + δ CH	Ring quinoline
1264	ν_a C-O-φ	
1166	δ ip CH	Ring quinoline
1117	ν C-C + δ CC	Ring quinoline
1055	ν_a C-O-φ	

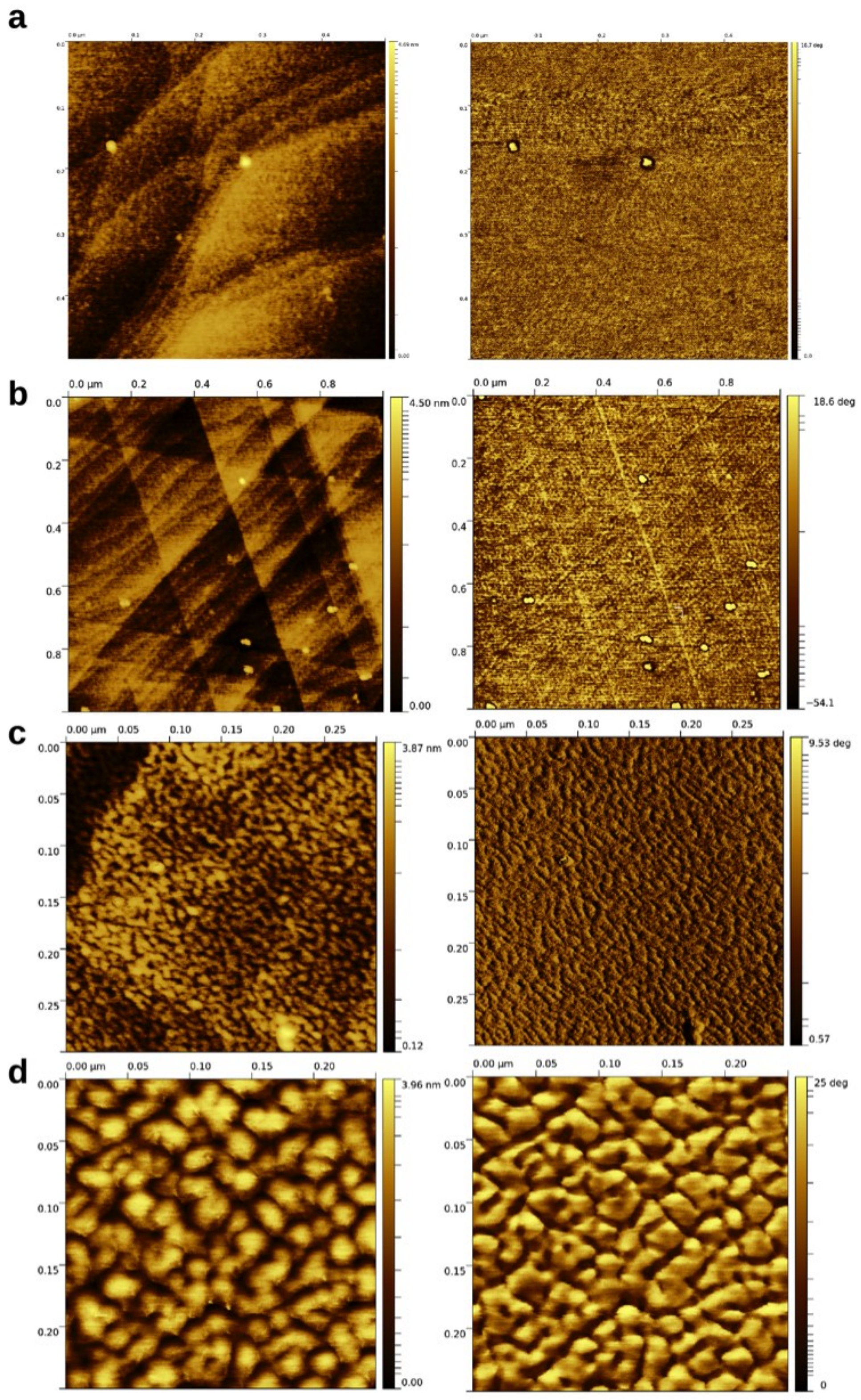

Figure S7. AFM images in height (left) and phase (right) mode of monolayers prepared with a) **Q5**; b) **Q9**; c) **Q17**; d) **Q33**.

Monolayers on SiO_2 of QA5-QA17

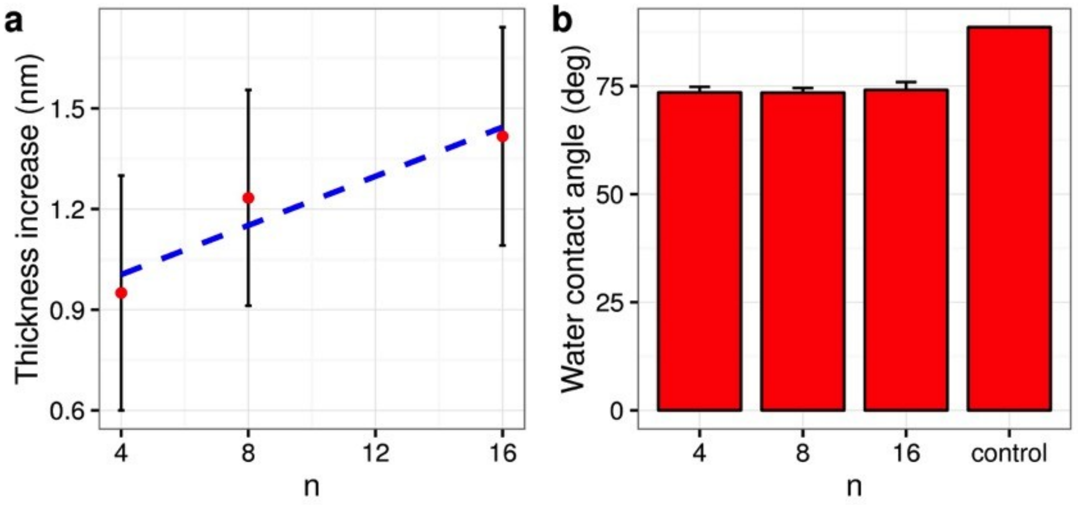

Figure S8 Monolayers of AQ5-AQ17 on SiO_2: a) Thickness increase (taken as total thickness of the organic layer minus thickness of the underlying azide monolayers measured in controls experiments) determined by ellipsometry. The blue line indicates linear fitting; b) Water contact angle measurements, where *control* measurements were carried out in samples treated with the same catalytic solutions without foldamer. Values represent the average of three independent monolayer preparations ± standard deviation. *n* denotes number of quinoline units -1 (see Figure 1 in main text)

Supplementary analysis of the resistance distributions.

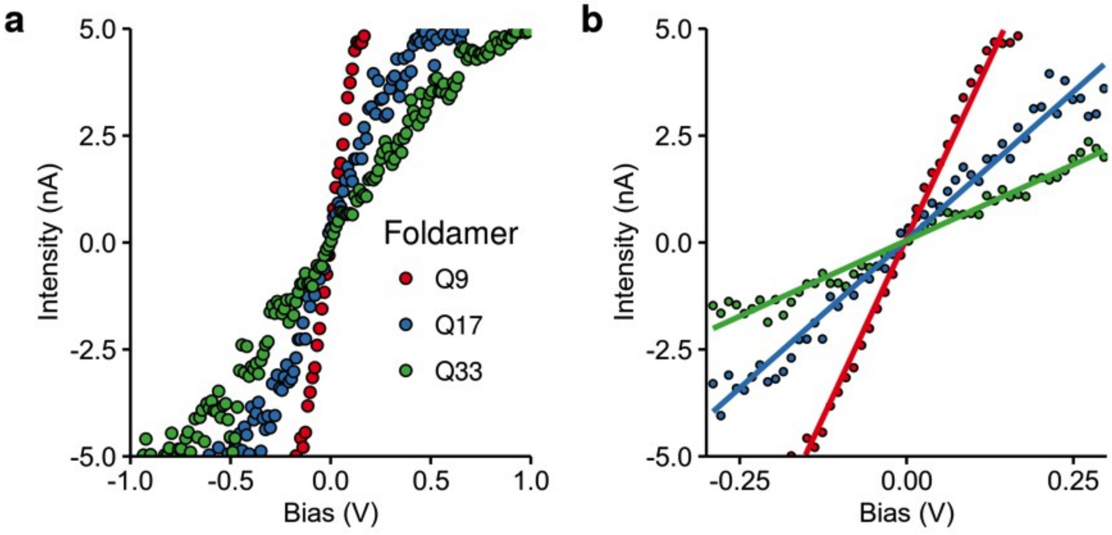

Figure S9. a) Representative *I-V* curves obtained from MOM junctions of **Q9**, **Q17** and **Q33** assembled on gold; b) Magnification at lower biases of the same curves. Lines indicate a linear fit on the ± 0.3 range bias.

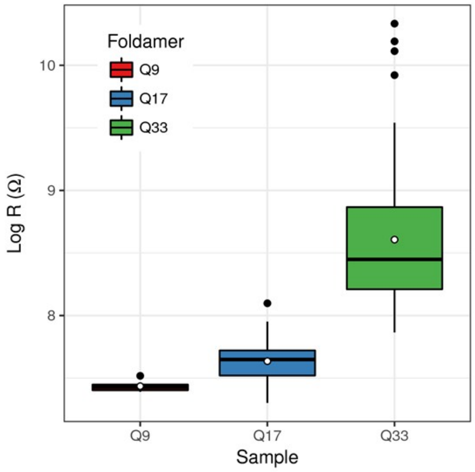

Figure S10. Statistical parameters for the full distributions represented as boxplots of log resistance (ohms) measurements at 1 nN on junctions prepared with monolayers of **Q9-Q33** on gold. The average resistance is represented with points in white. Black dots represent experimental points which are at a distance higher than 1.5 times the interquartile range from the hinge.

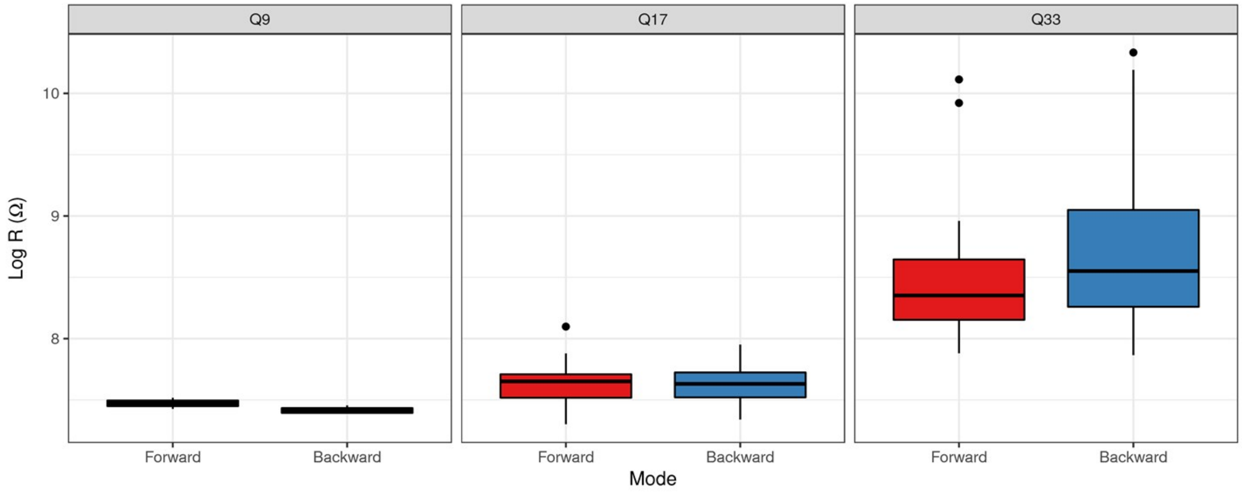

Figure S11. Box and whiskers plots for log resistance (ohms) measured scanning the applied bias forward and backwards on junctions of **Q9**-**Q33** at 1 nN. Black dots represent experimental points which are at a distance higher than 1.5 times the interquartile range from the hinge.

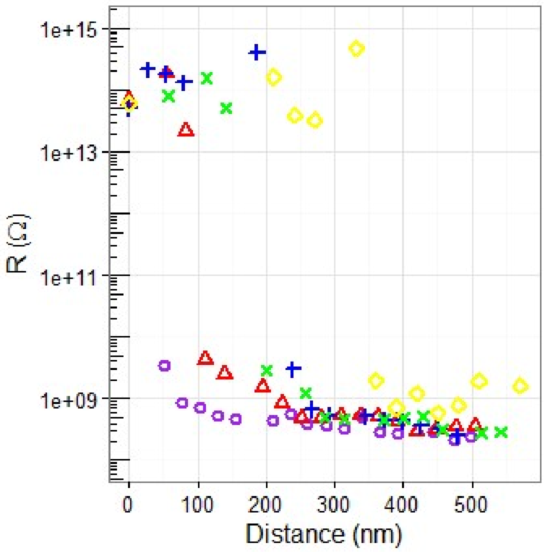

Figure S12. Conductivity profiles obtained in azideundecane monolayers on SiO_2. Different symbols denote different experiments.

NMR spectra

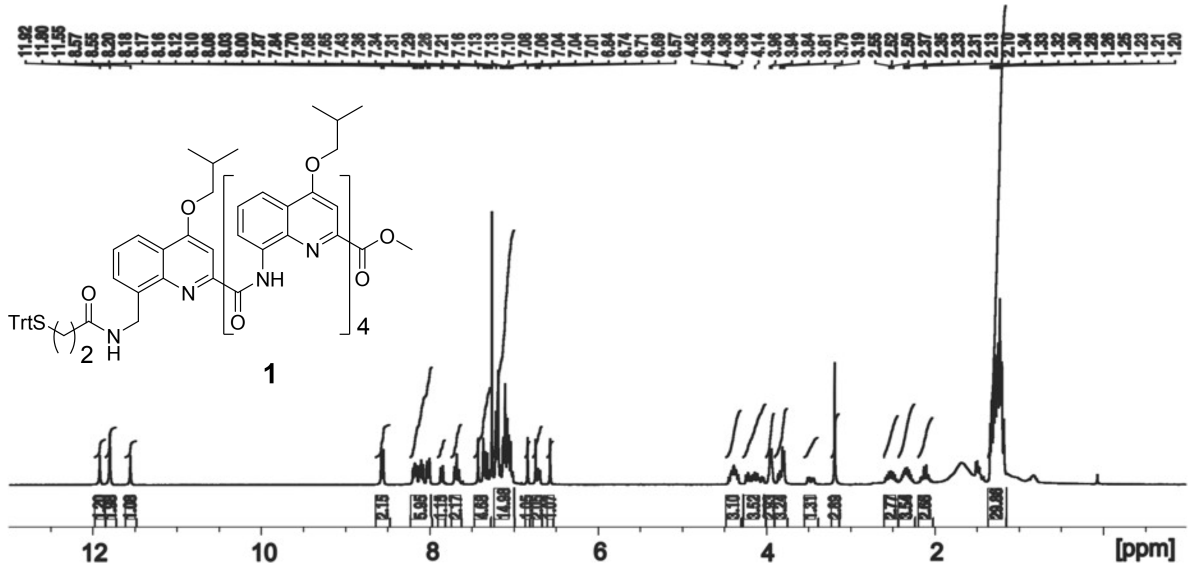

Figure S13. ^{1}H NMR spectrum of compound **1** in $CDCl_3$ at RT measured on a 300 MH_Z Bruker NMR

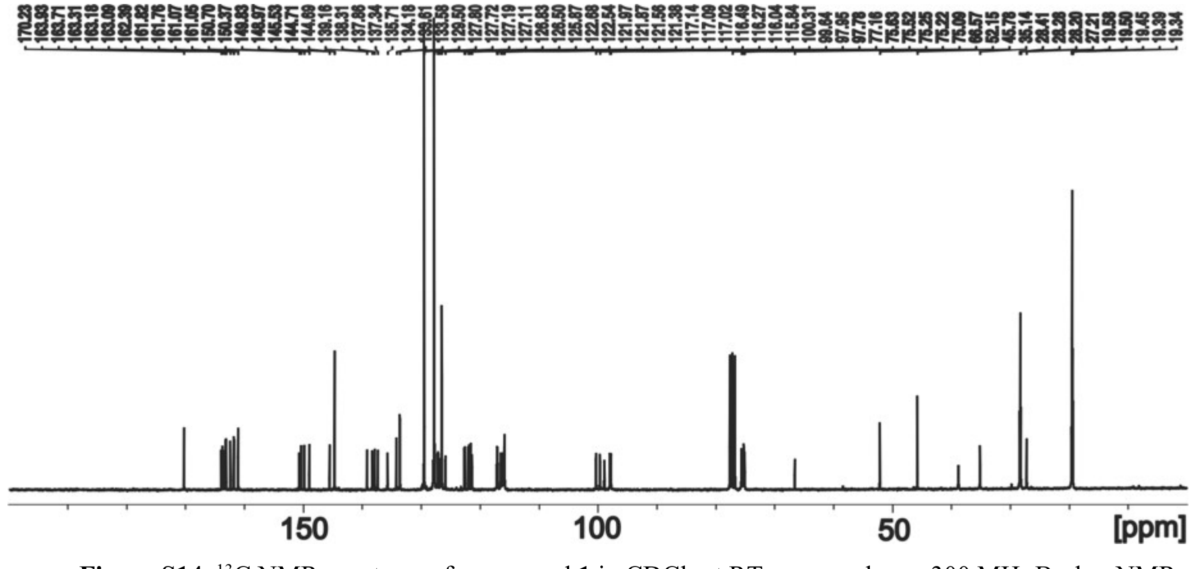

Figure S14. ^{13}C NMR spectrum of compound **1** in $CDCl_3$ at RT measured on a 300 MH_Z Bruker NMR

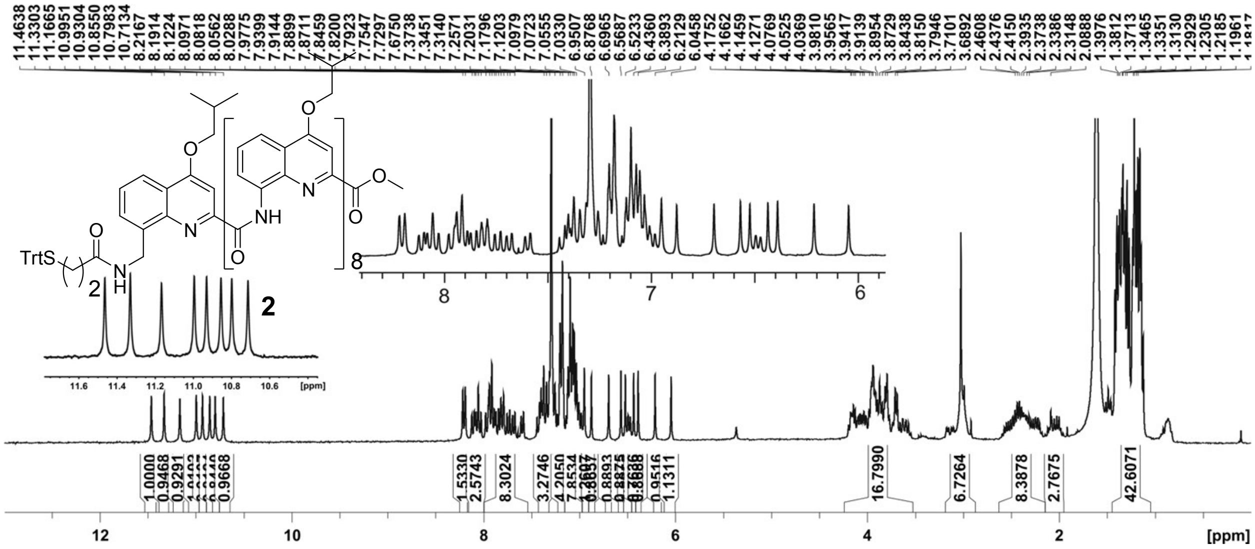

Figure S15. ^{1}H NMR spectrum of compound **2** in $CDCl_3$ at RT measured on a 300 MH_Z Bruker NMR

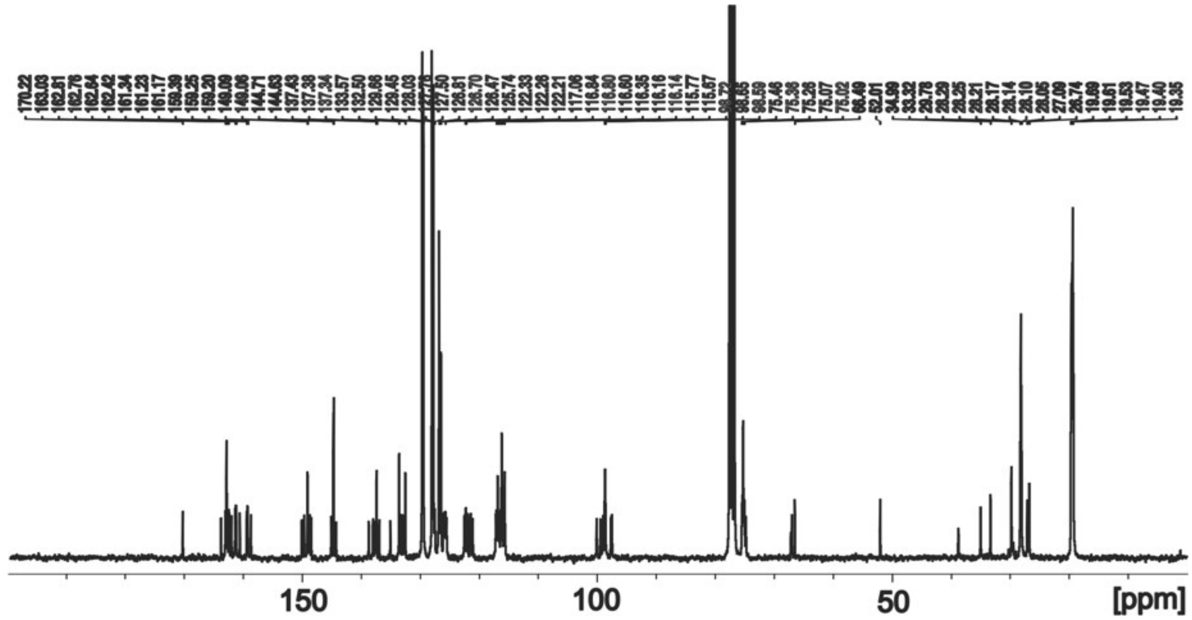

Figure S16. ^{13}C NMR spectrum of compound **2** in $CDCl_3$ at RT measured on a 300 MH_Z Bruker NMR

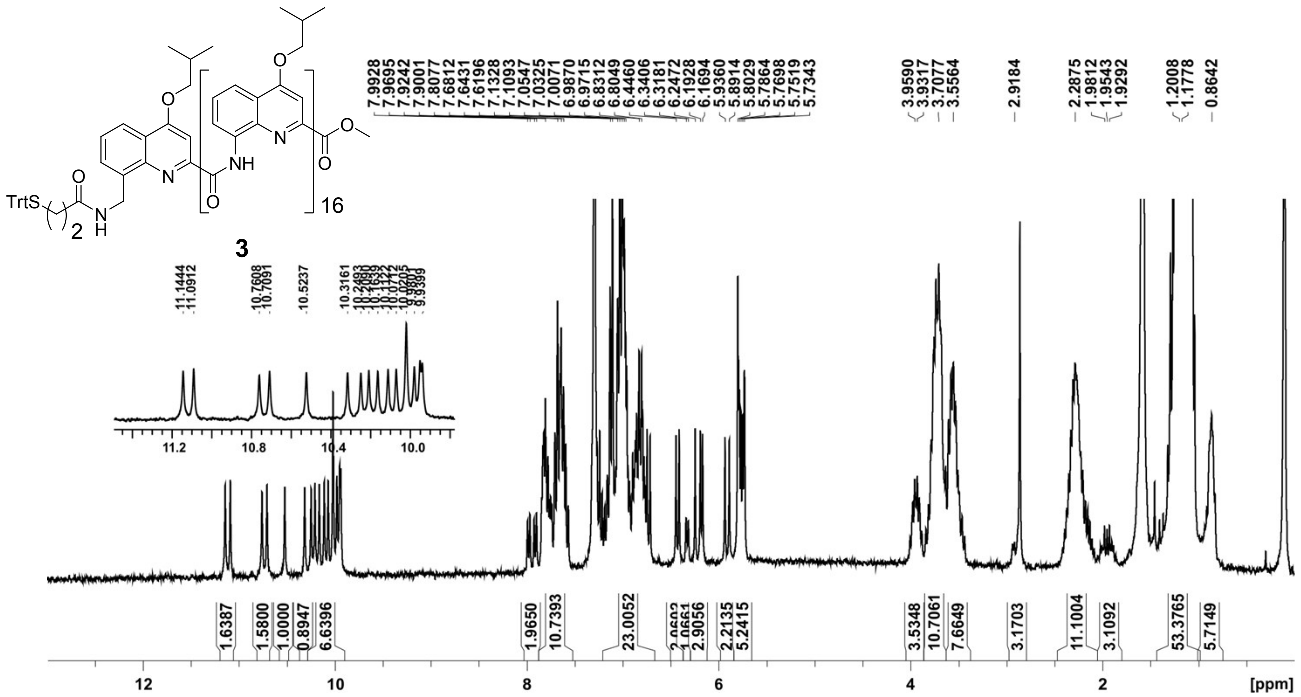

Figure S19. ^{1}H NMR spectrum of compound **3** in $CDCl_3$ at RT measured on a 300 MH_Z Bruker NMR

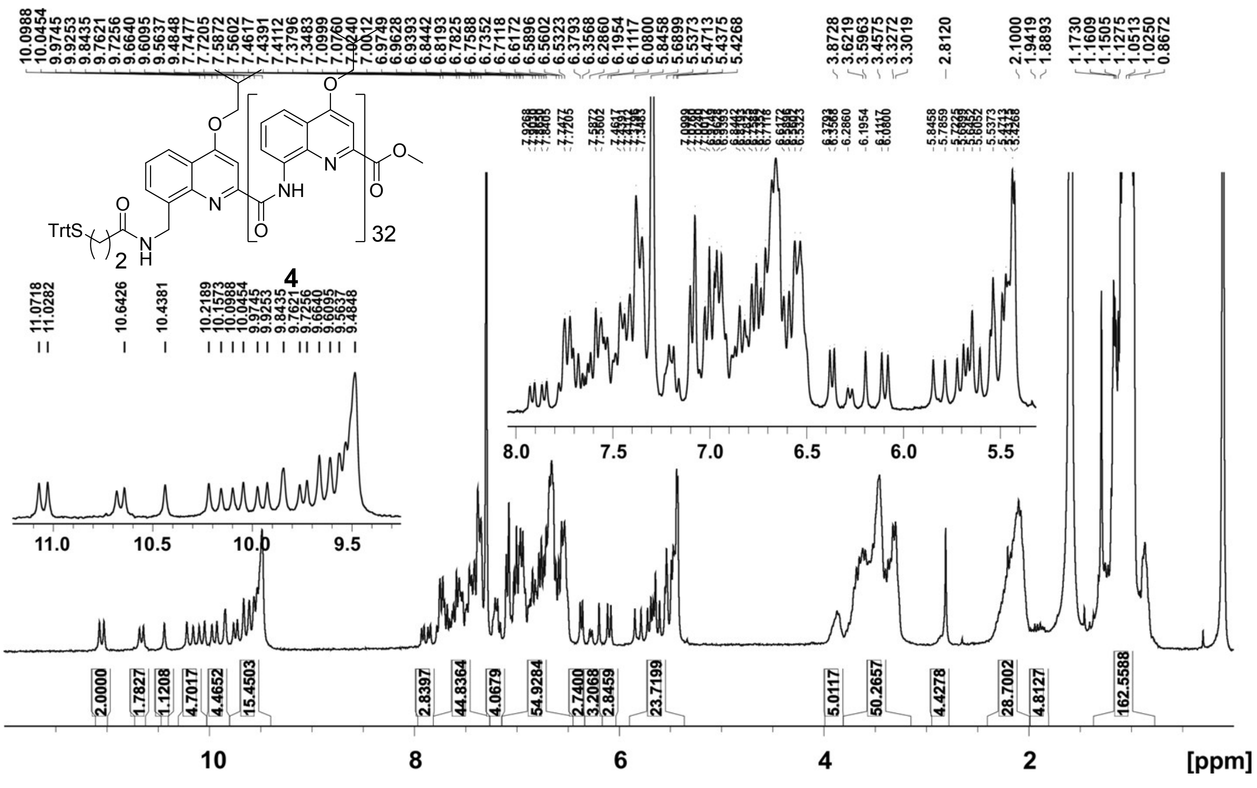

Figure S18. ^{1}H NMR spectrum of compound **4** in $CDCl_3$ at RT measured on a 300 MH_Z Bruker NMR

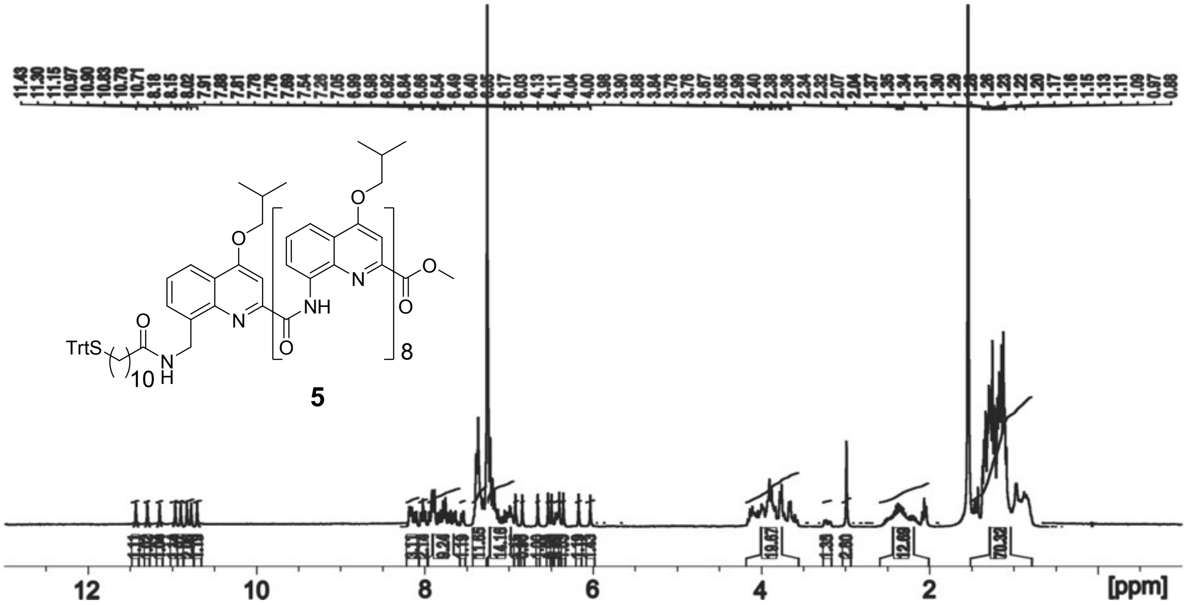

Figure S19. ^{1}H NMR spectrum of compound **5** in $CDCl_3$ at RT measured on a 300 MH_Z Bruker NMR

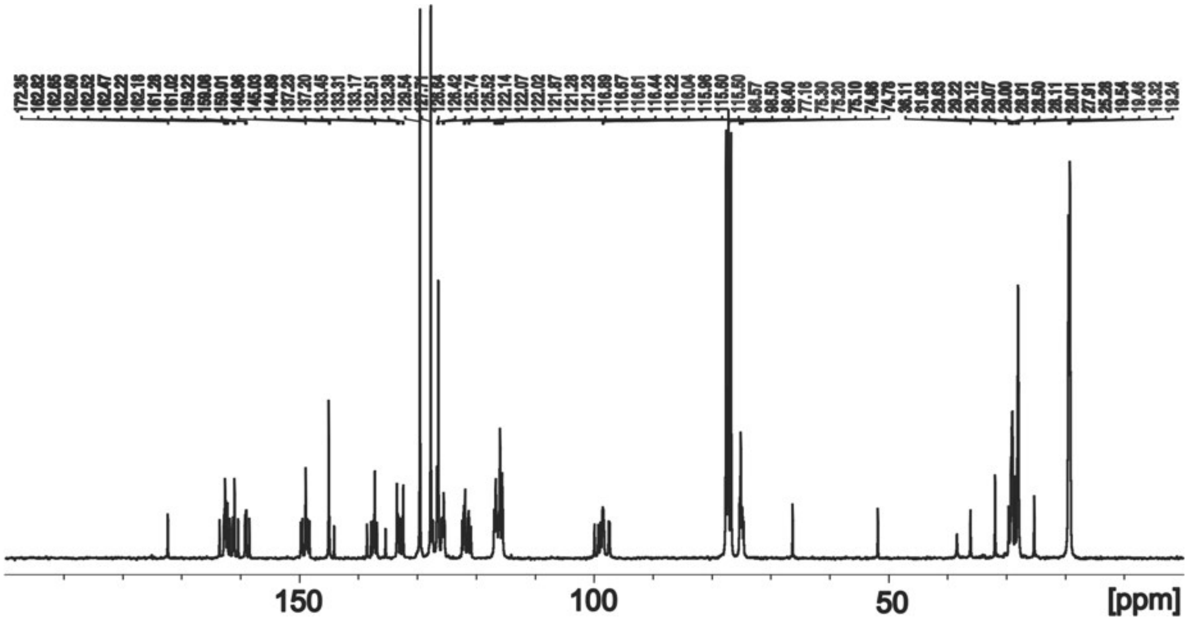

Figure S20. ^{13}C NMR spectrum of compound **5** in $CDCl_3$ at RT measured on a 300 MH_Z Bruker NMR

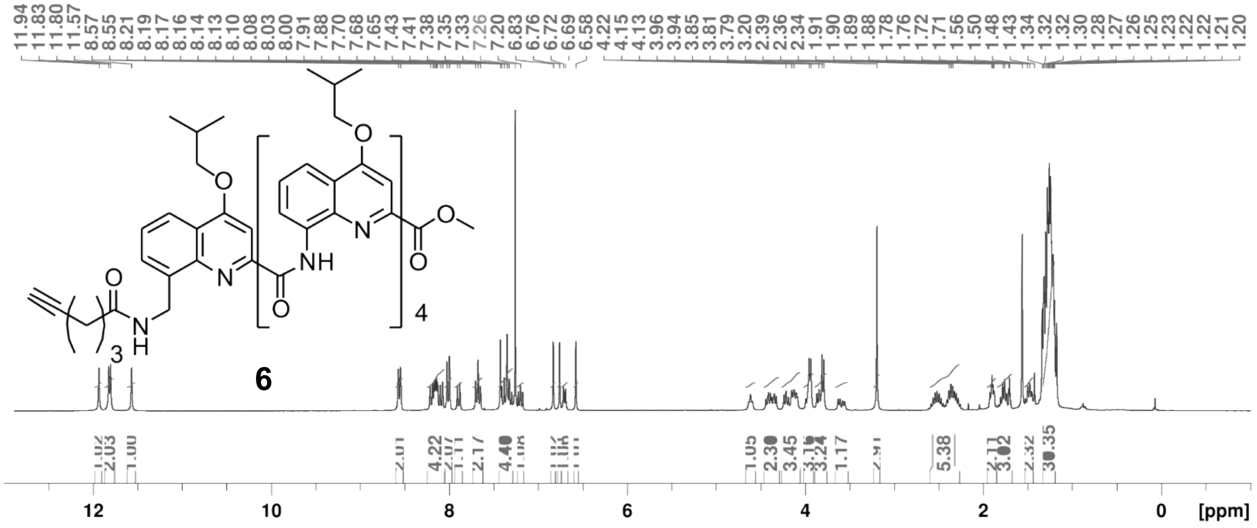

Figure S21. ^{1}H NMR spectrum of compound **6** in $CDCl_3$ at RT measured on a 300 MH_Z Bruker NMR

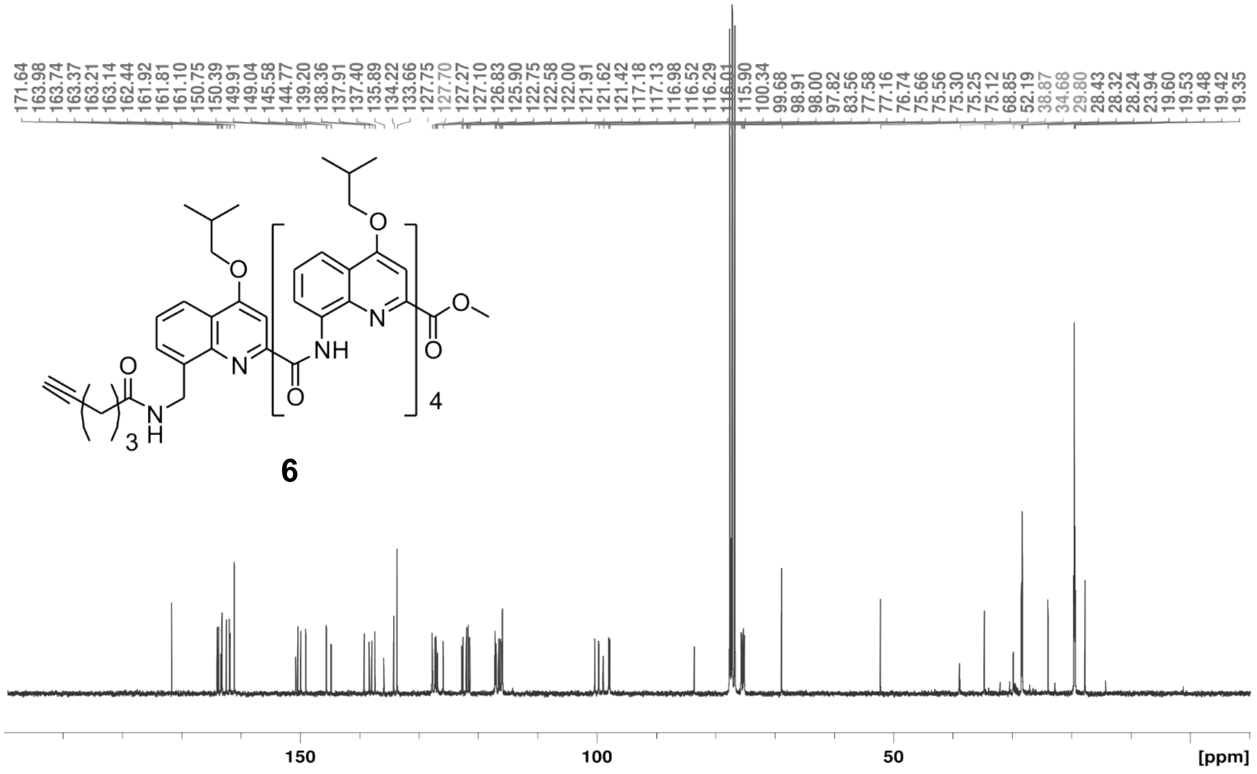

Figure S22. ^{13}C NMR spectrum of compound **6** in $CDCl_3$ at RT measured on a 300 MH_Z Bruker NMR

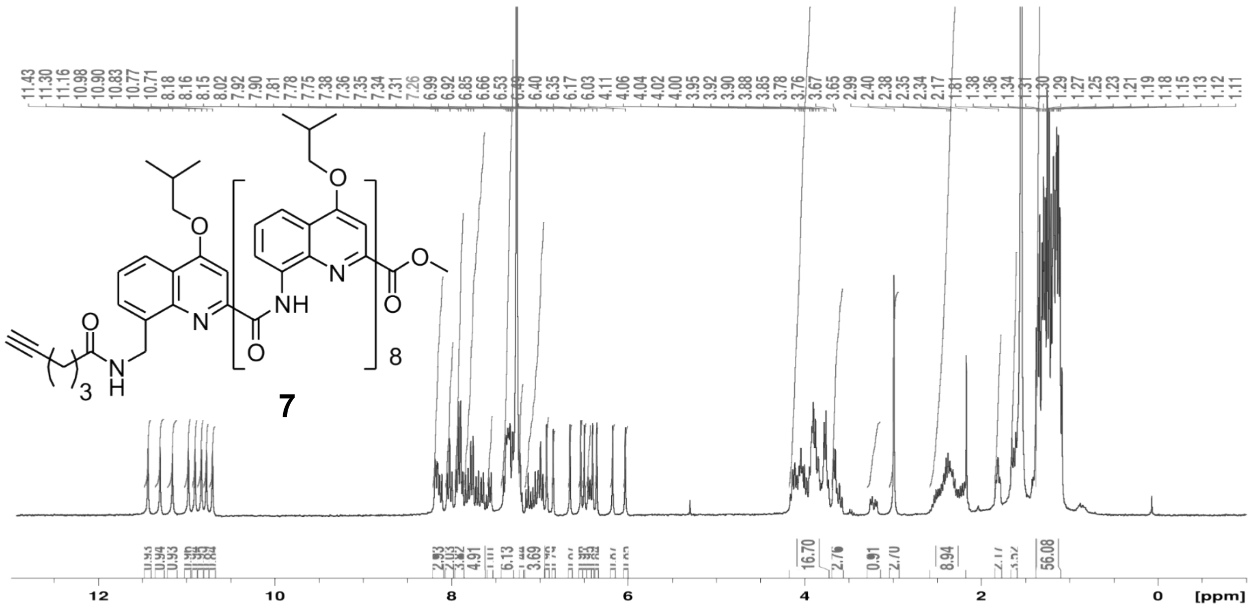

Figure S23. ^{1}H NMR spectrum of compound **7** in $CDCl_3$ at RT measured on a 300 MH_Z Bruker NMR

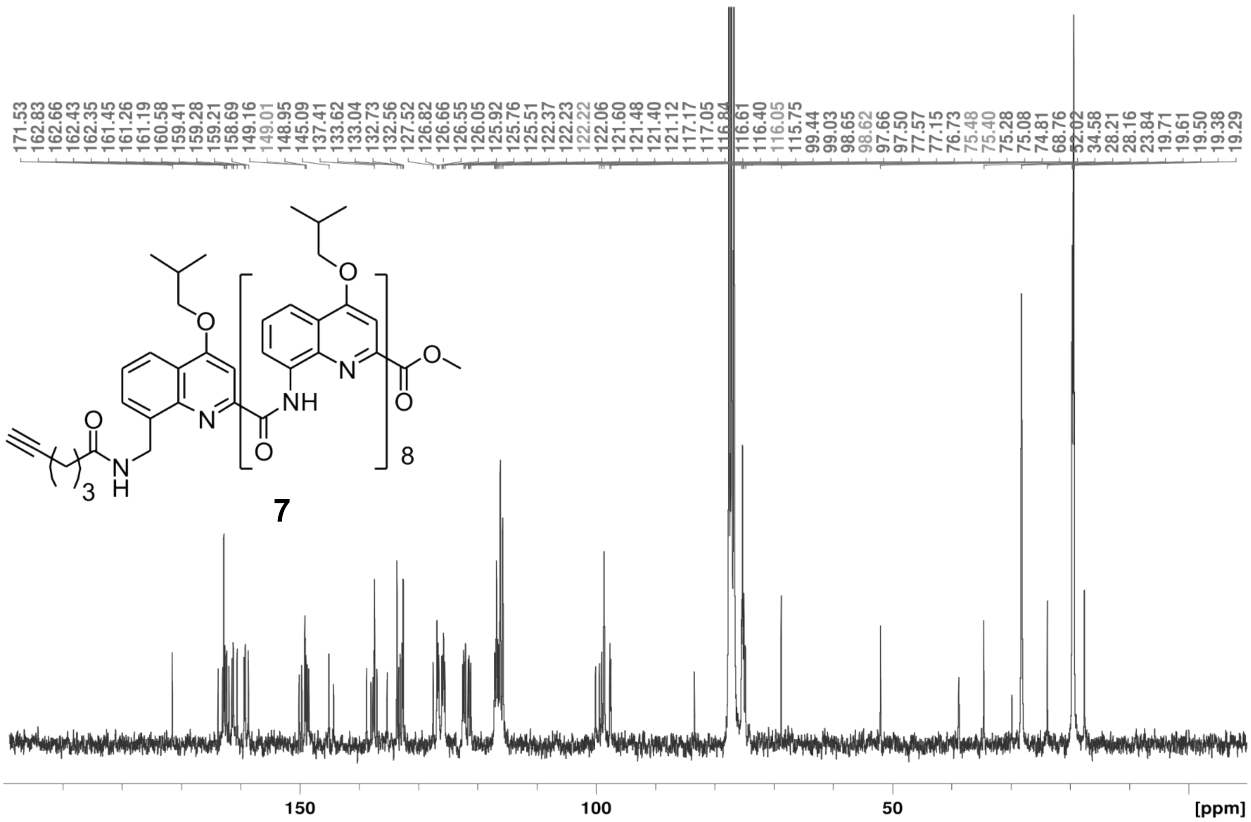

Figure S24. ^{13}C NMR spectrum of compound **7** in $CDCl_3$ at RT measured on a 300 MH_Z Bruker NMR

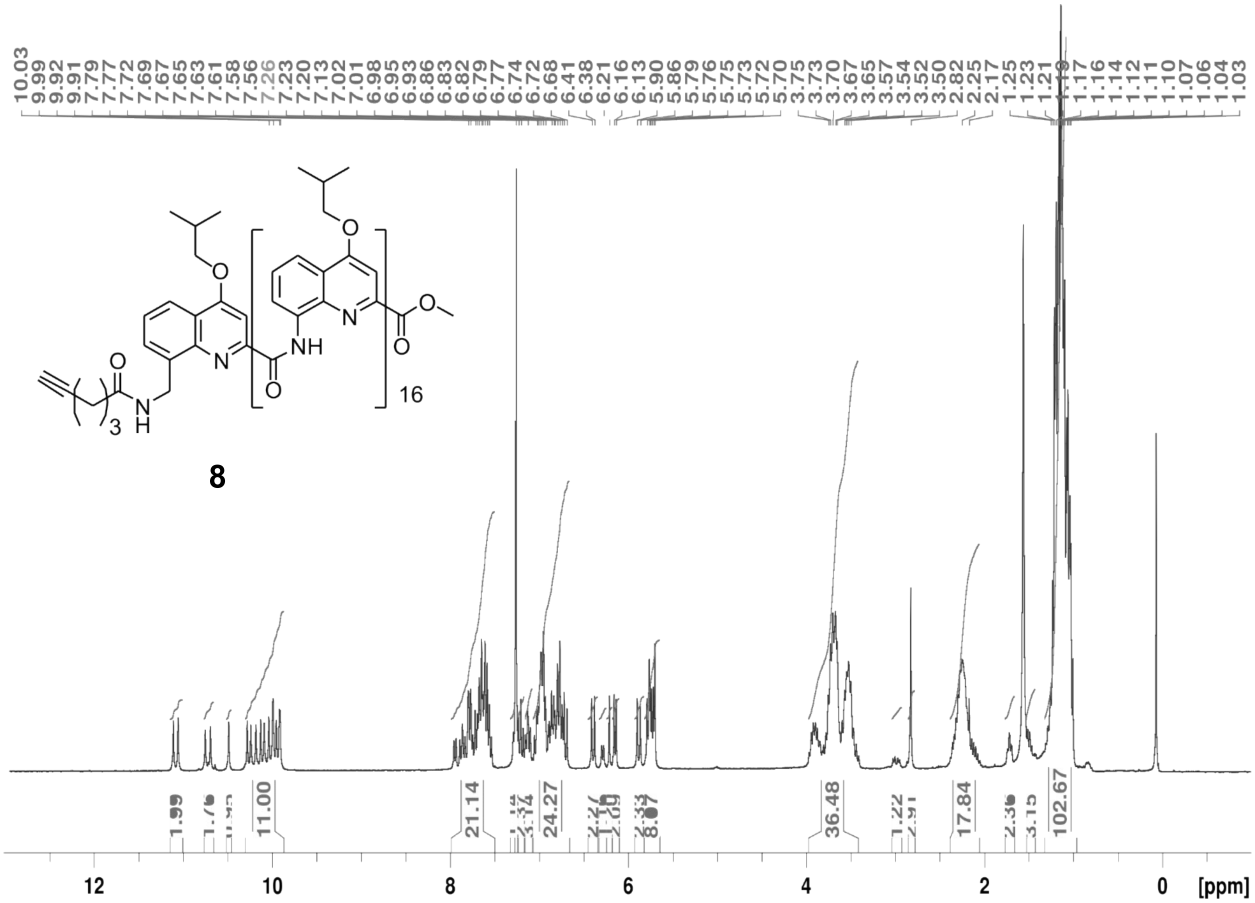

Figure S25. ^{1}H NMR spectrum of compound **8** in $CDCl_3$ at RT measured on a 300 MH_Z Bruker NMR

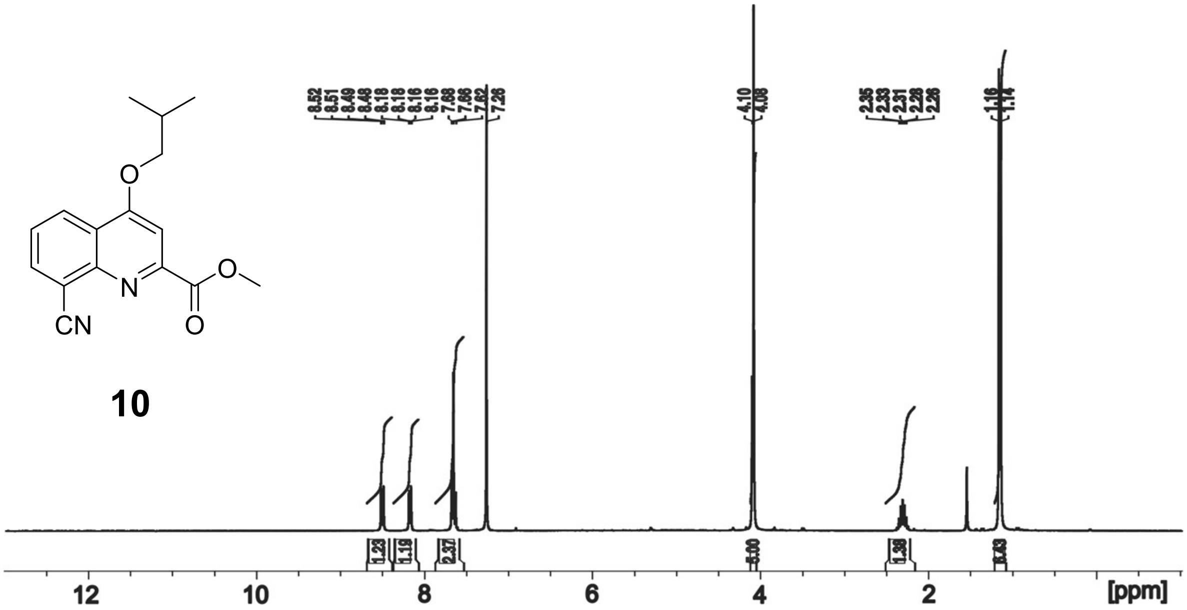

Figure S26. ^{1}H NMR spectrum of compound **10** in $CDCl_3$ at RT measured on a 300 MH_Z Bruker NMR

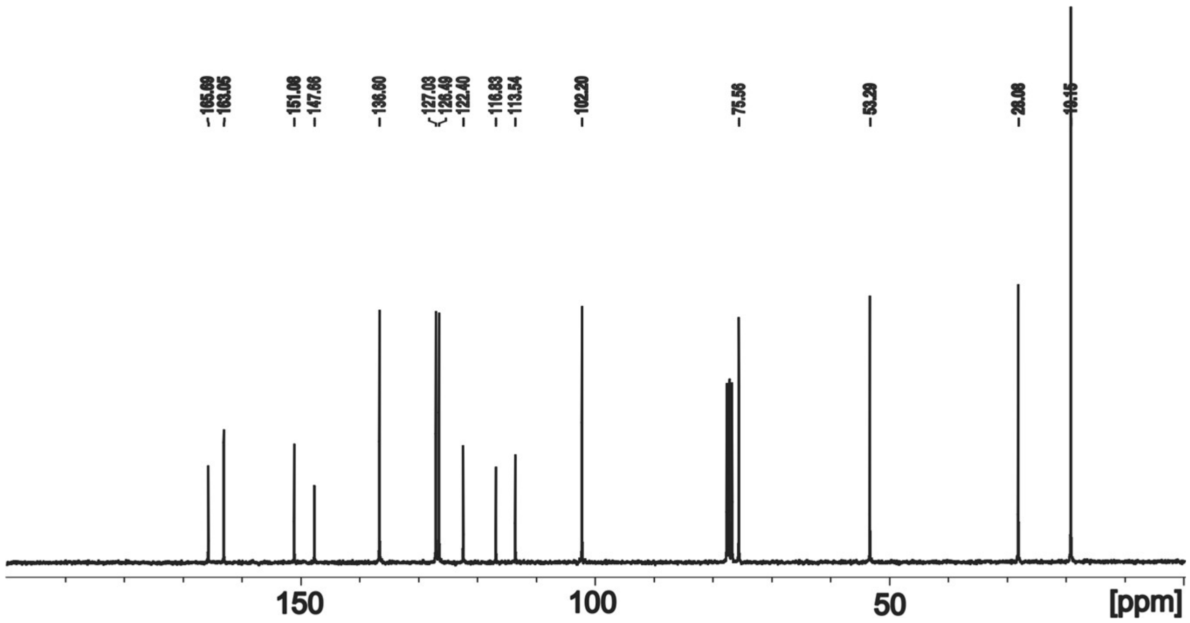

Figure S27. ^{13}C NMR spectrum of compound **10** in $CDCl_3$ at RT measured on a 300 MH_Z Bruker NMR

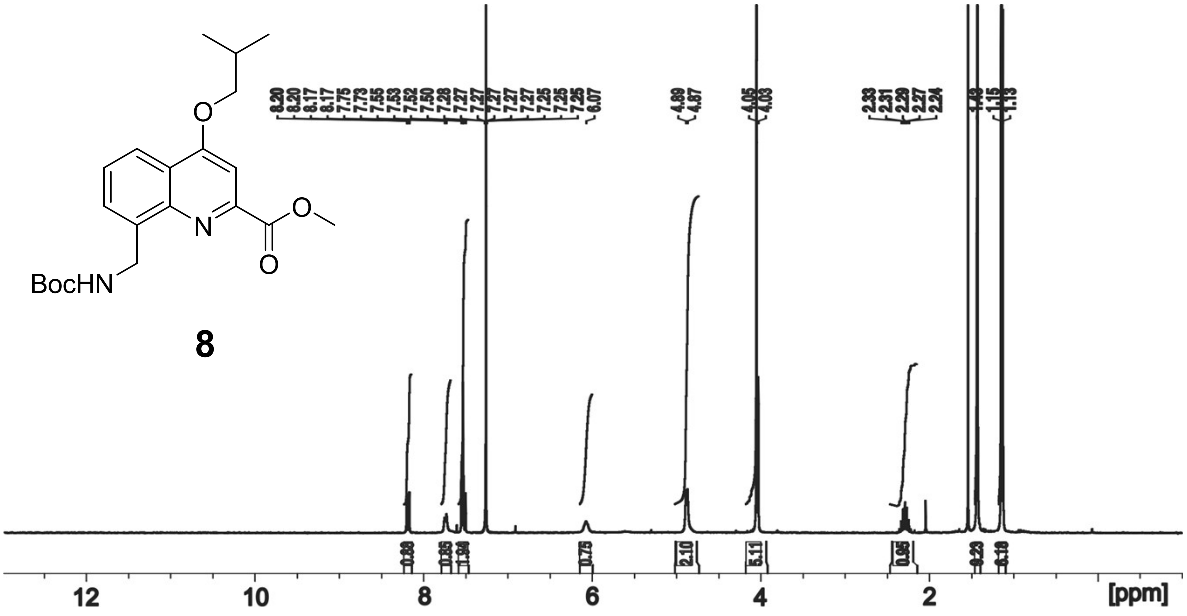

Figure S28. ^{1}H NMR spectrum of compound **11** in $CDCl_3$ at RT measured on a 300 MH_Z Bruker NMR

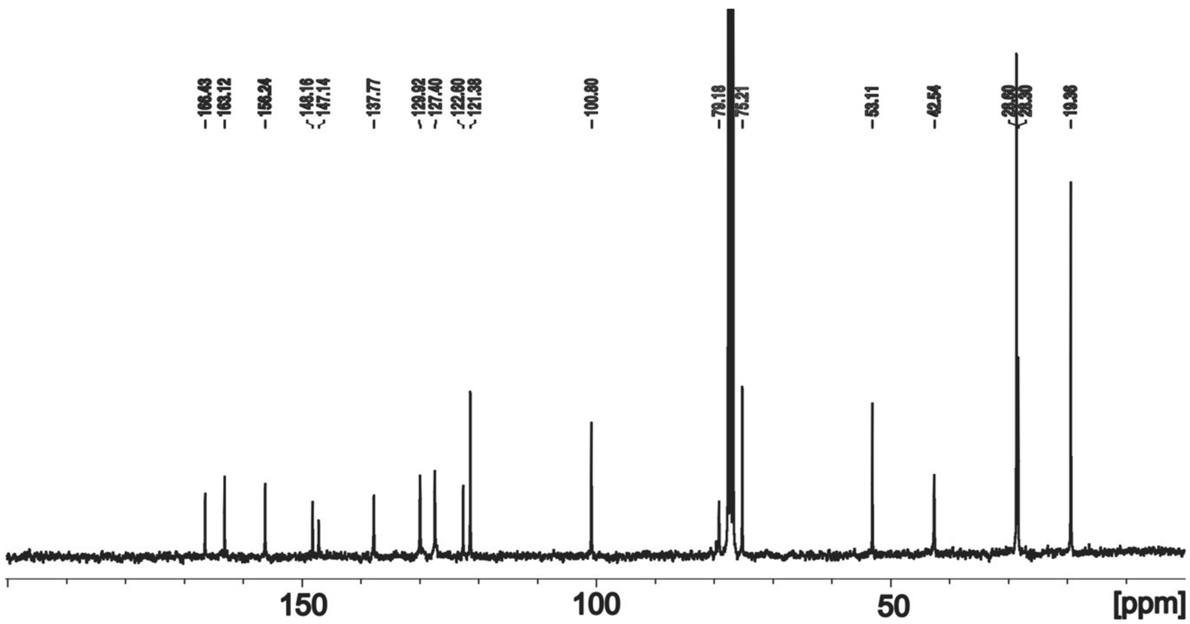

Figure S29. ^{13}C NMR spectrum of compound **11** in $CDCl_3$ at RT measured on a 300 MH_Z Bruker NMR

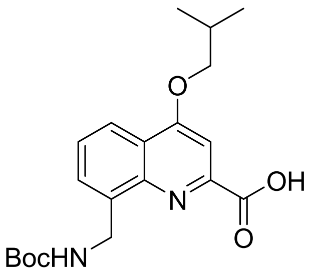

9

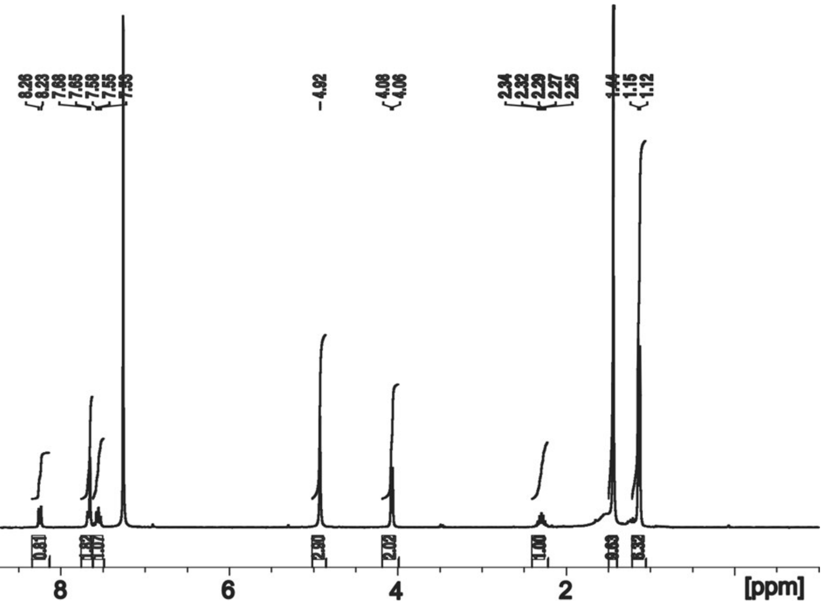

Figure S30. ^{1}H NMR spectrum of compound **12** in $CDCl_3$ at RT measured on a 300 MH_Z Bruker NMR

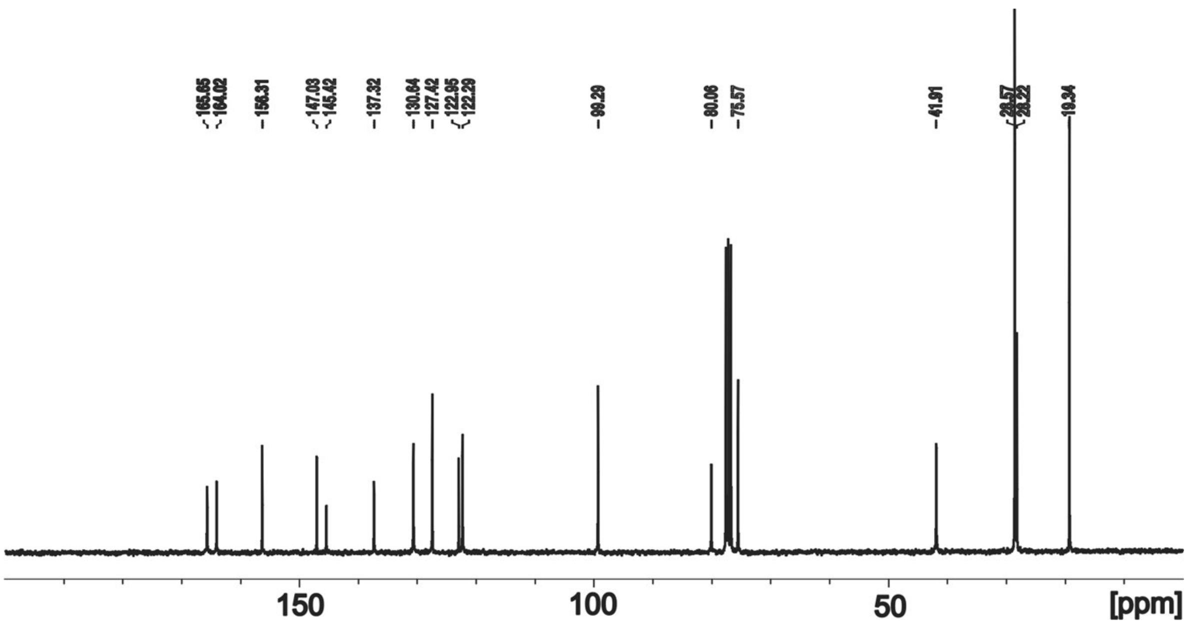

Figure S31. ^{13}C NMR spectrum of compound **12** in $CDCl_3$ at RT measured on a 300 MH_Z Bruker NMR

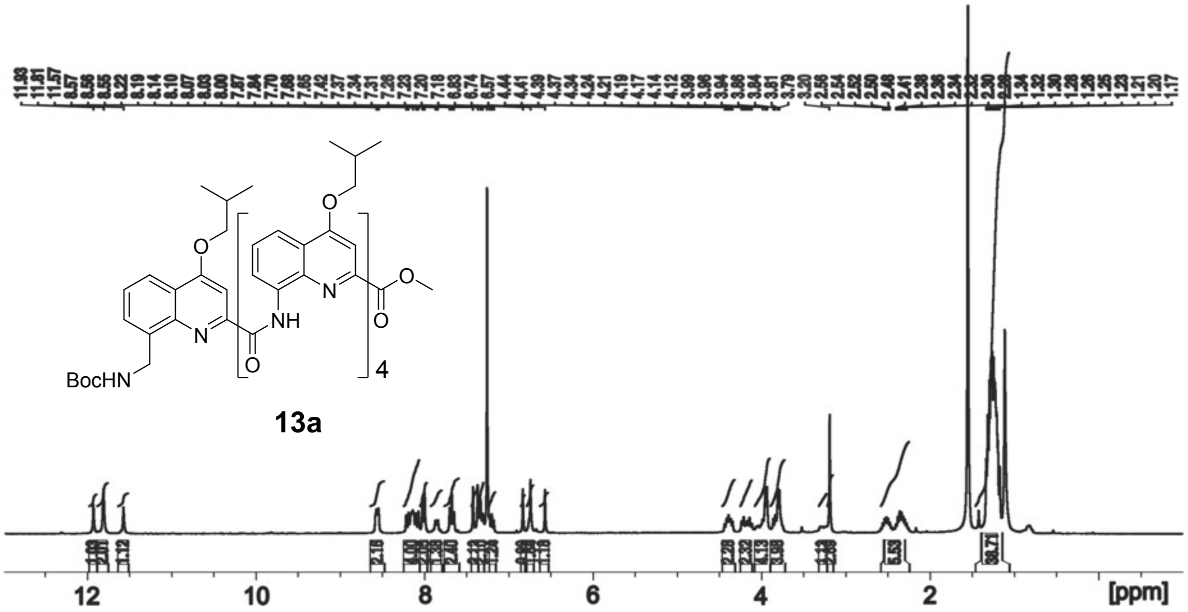

Figure S32. ^{1}H NMR spectrum of compound **13a** in $CDCl_3$ at RT measured on a 300 MH_Z Bruker NMR

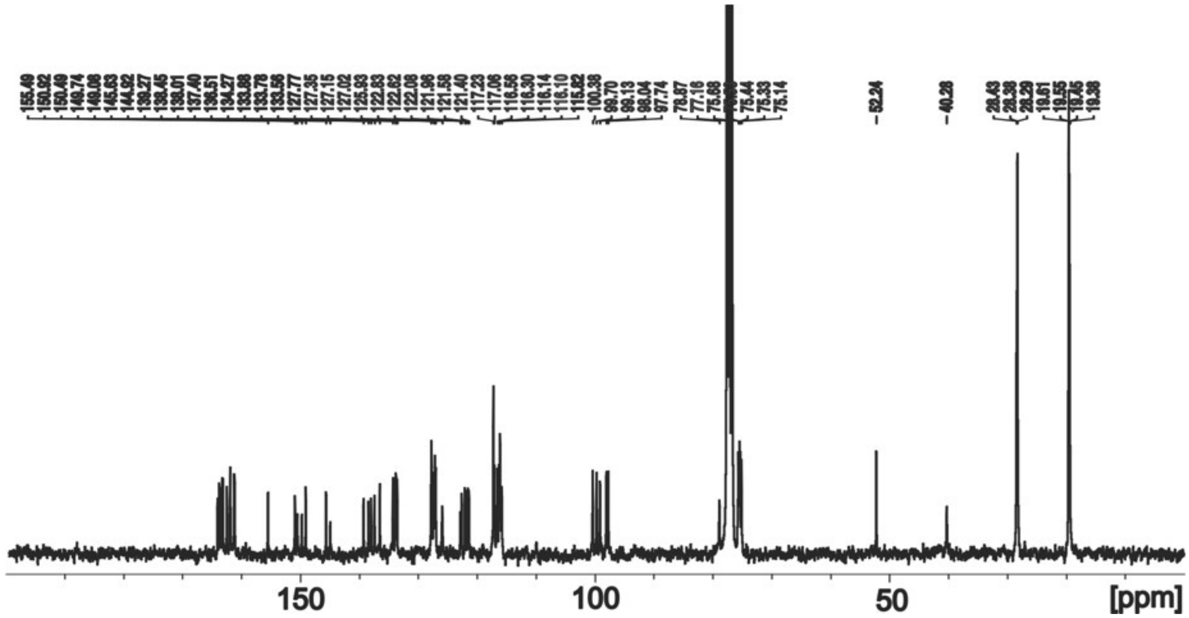

Figure S33. ^{13}C NMR spectrum of compound **13a** in $CDCl_3$ at RT measured on a 300 MH_Z Bruker NMR

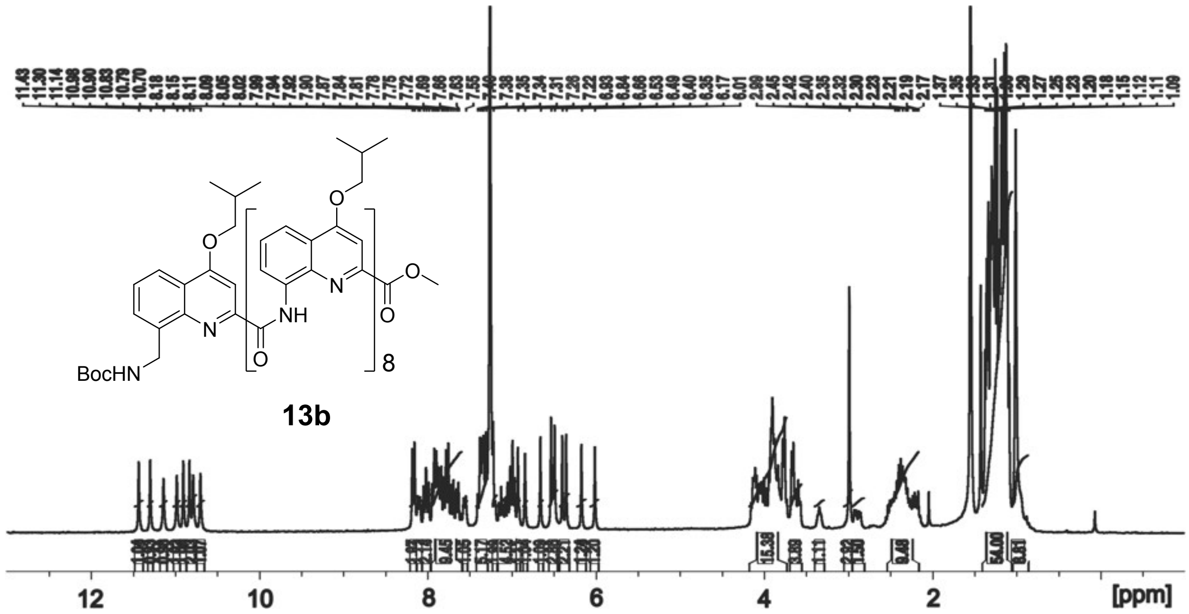

Figure S34. ^{1}H NMR spectrum of compound **13b** in $CDCl_3$ at RT measured on a 300 MH_Z Bruker NMR

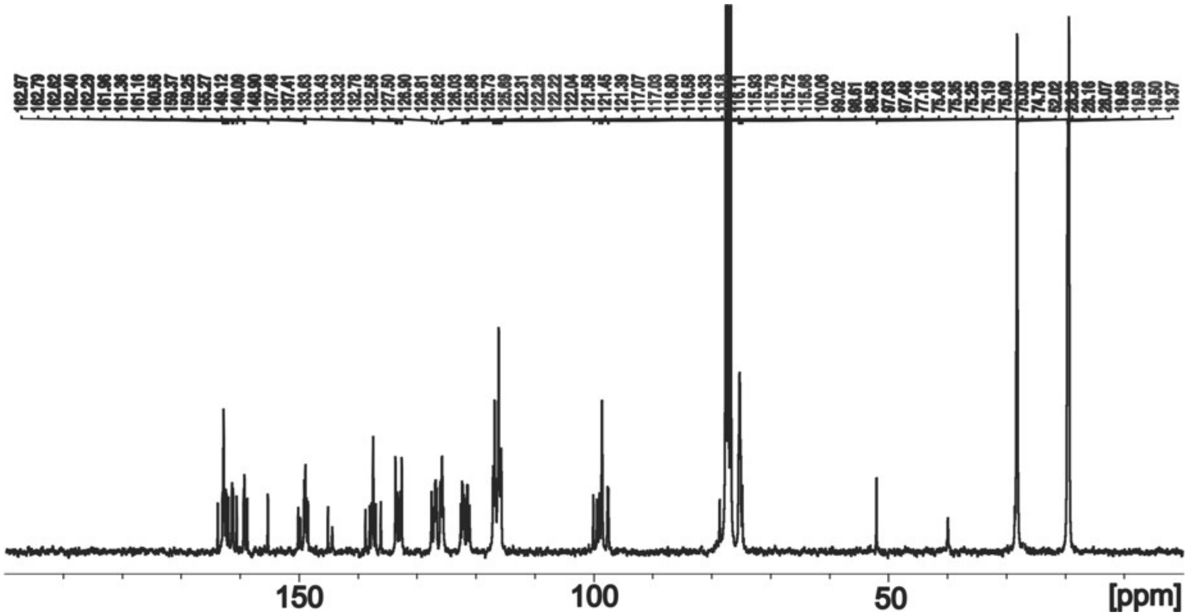

Figure S35. ^{13}C NMR spectrum of compound **13b** in $CDCl_3$ at RT measured on a 300 MH_Z Bruker NMR

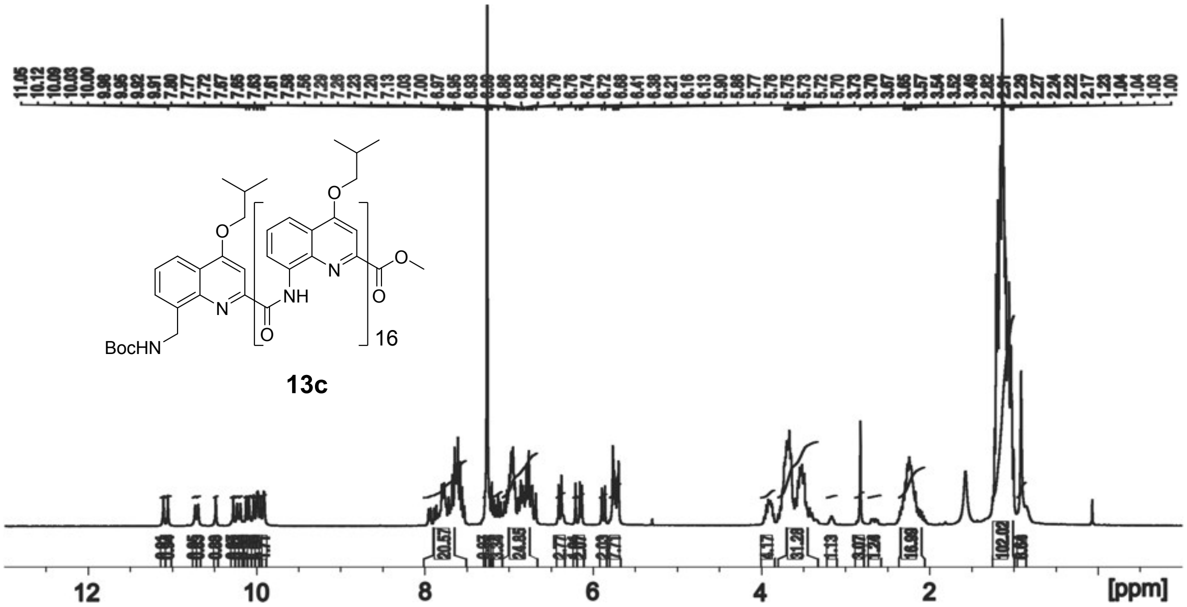

Figure S36. ^{1}H NMR spectrum of compound **13c** in $CDCl_3$ at RT measured on a 300 MH_Z Bruker NMR

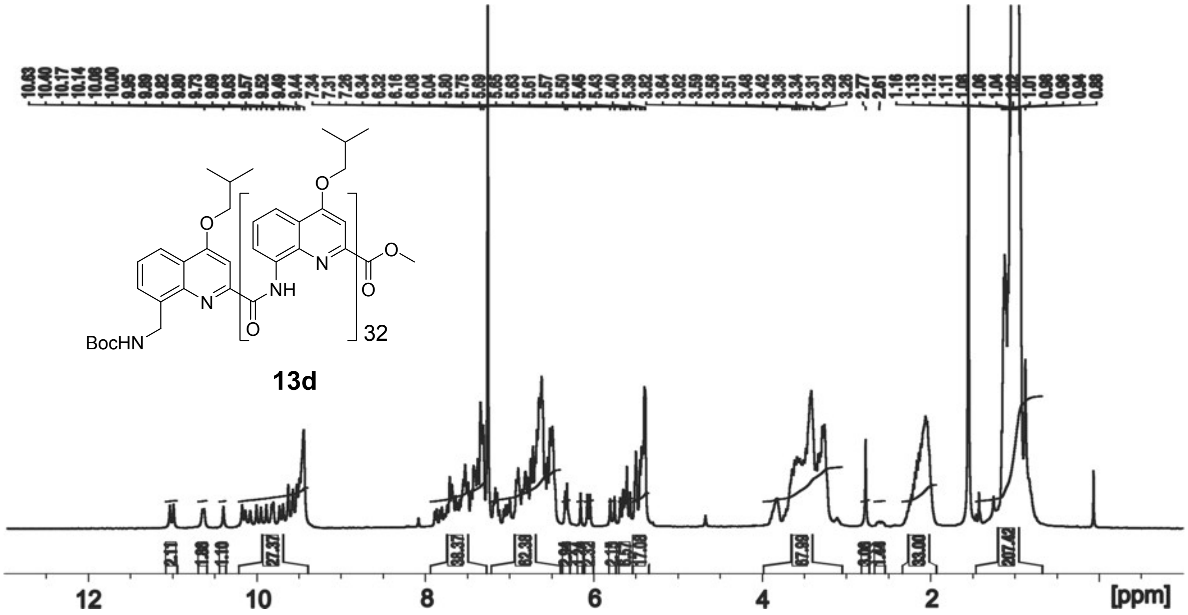

Figure S37. ^{1}H NMR spectrum of compound **13d** in $CDCl_3$ at RT measured on a 300 MH_Z Bruker NMR

I-V curves

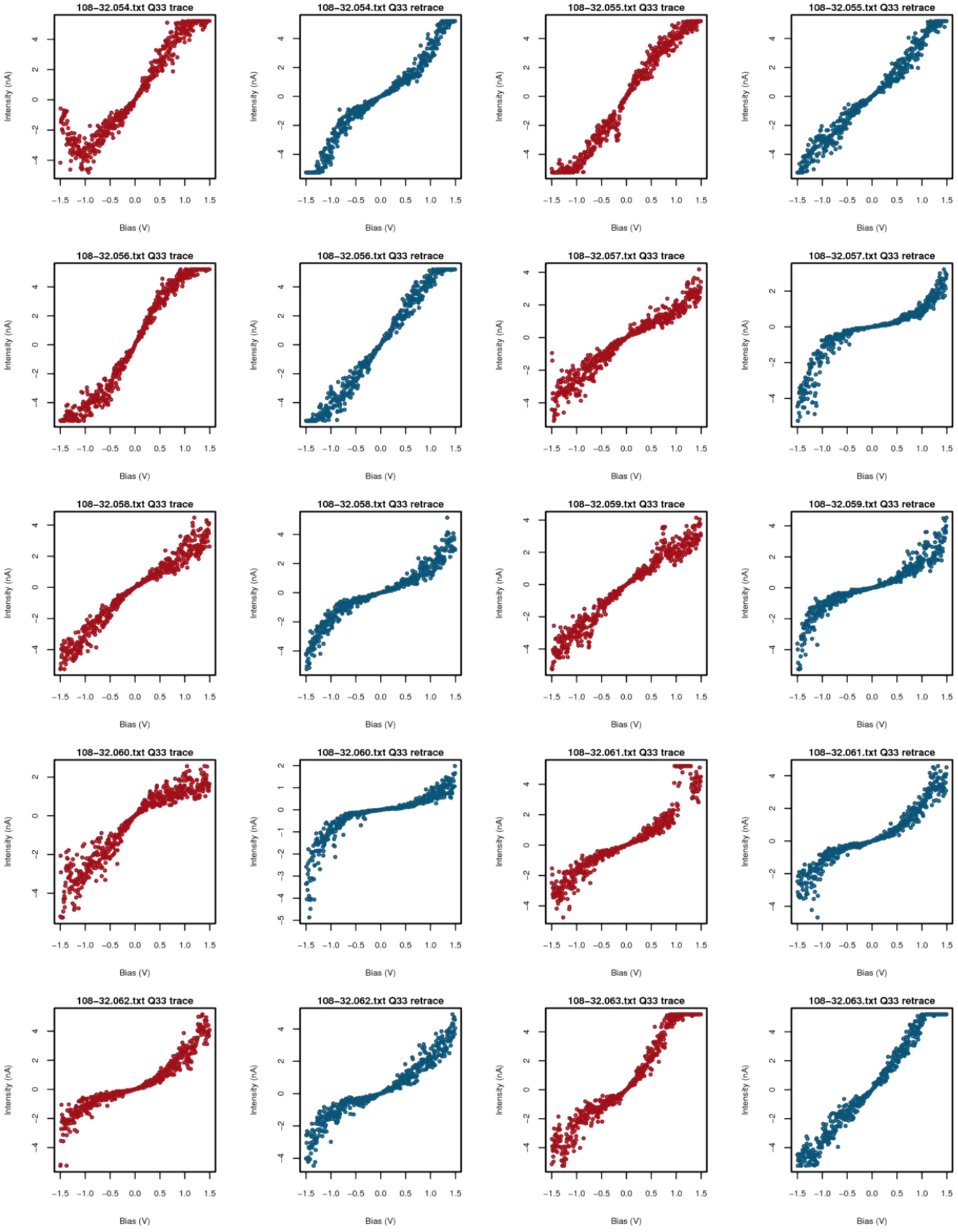

Figure S38. *I-V* curves acquired for monolayers of foldamer **Q9**, **Q17** and **Q33** at ca 1 nN. Red and blue points represent data acquired by reversing the bias forward and backwards.

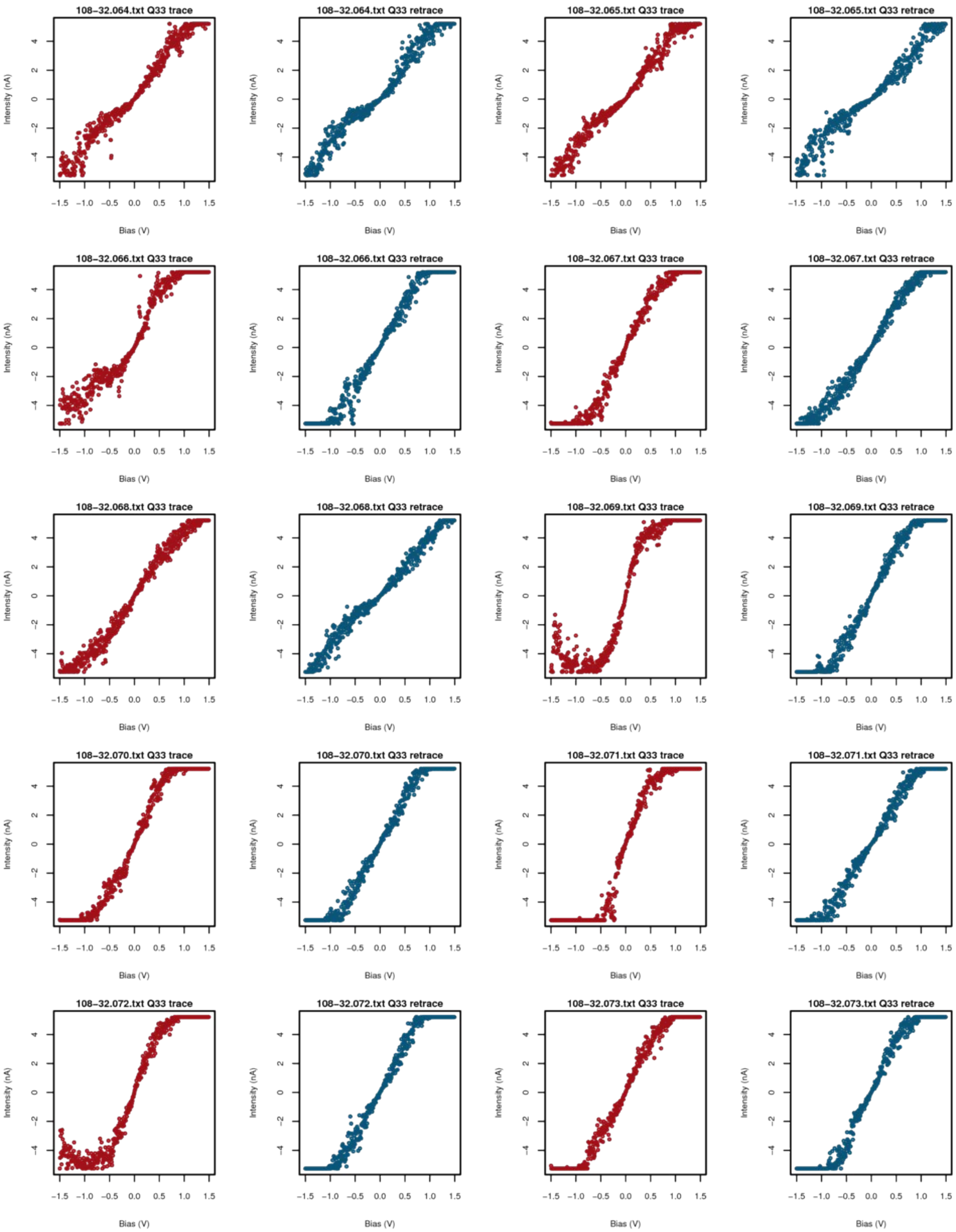

Continuation of Figure S38 (2/11)

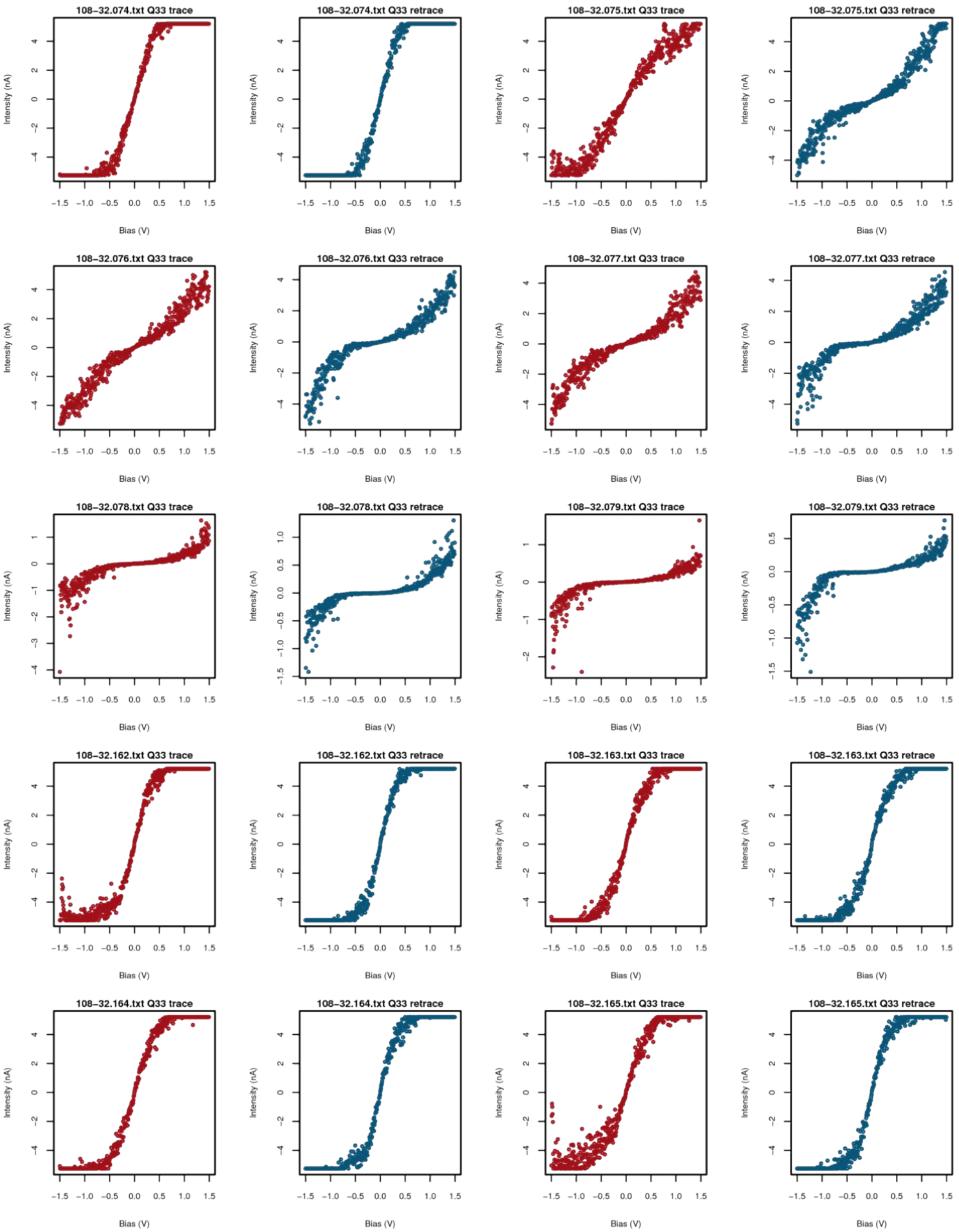

Continuation of Figure S38 (3/11)

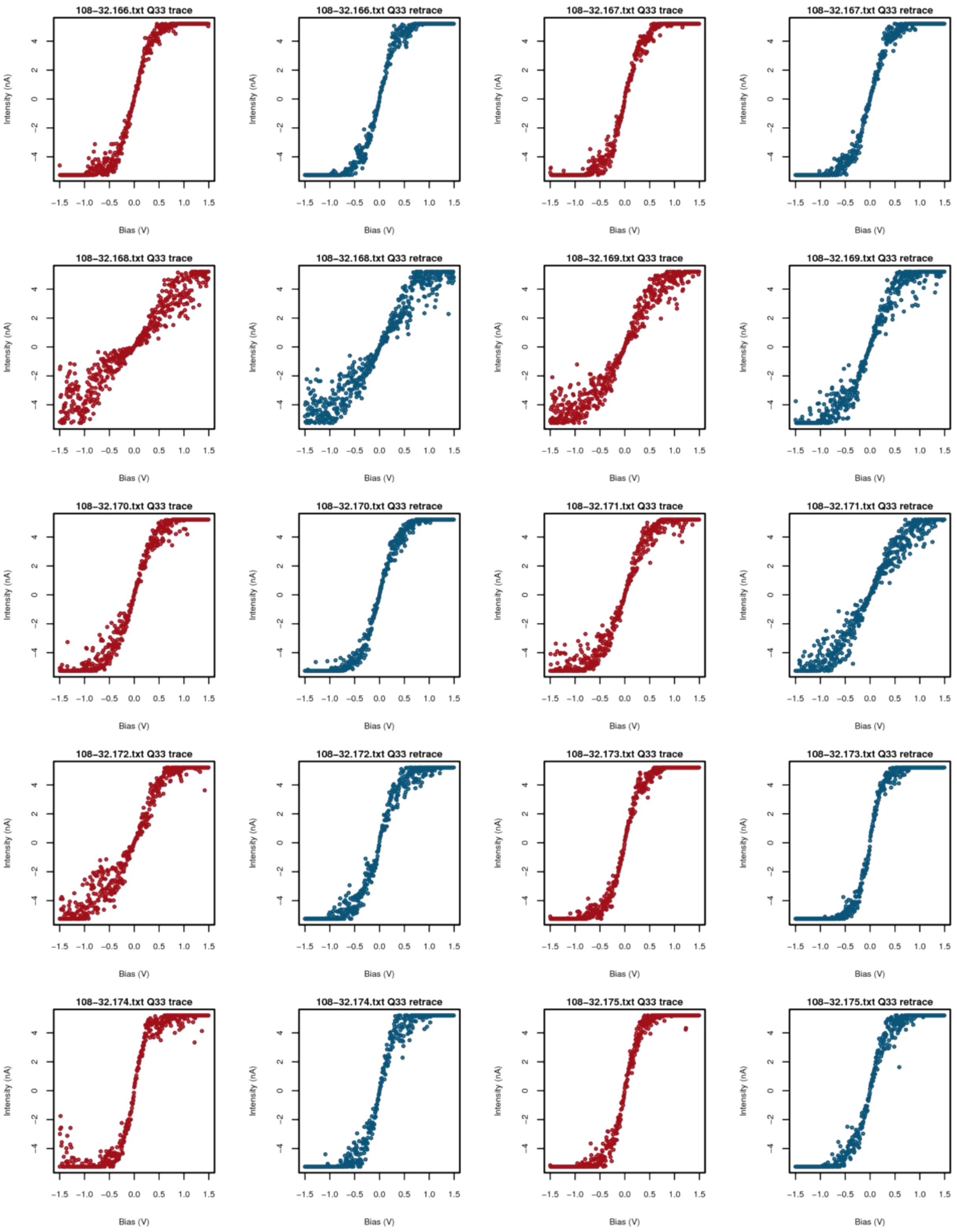

Continuation of Figure S38 (4/11)

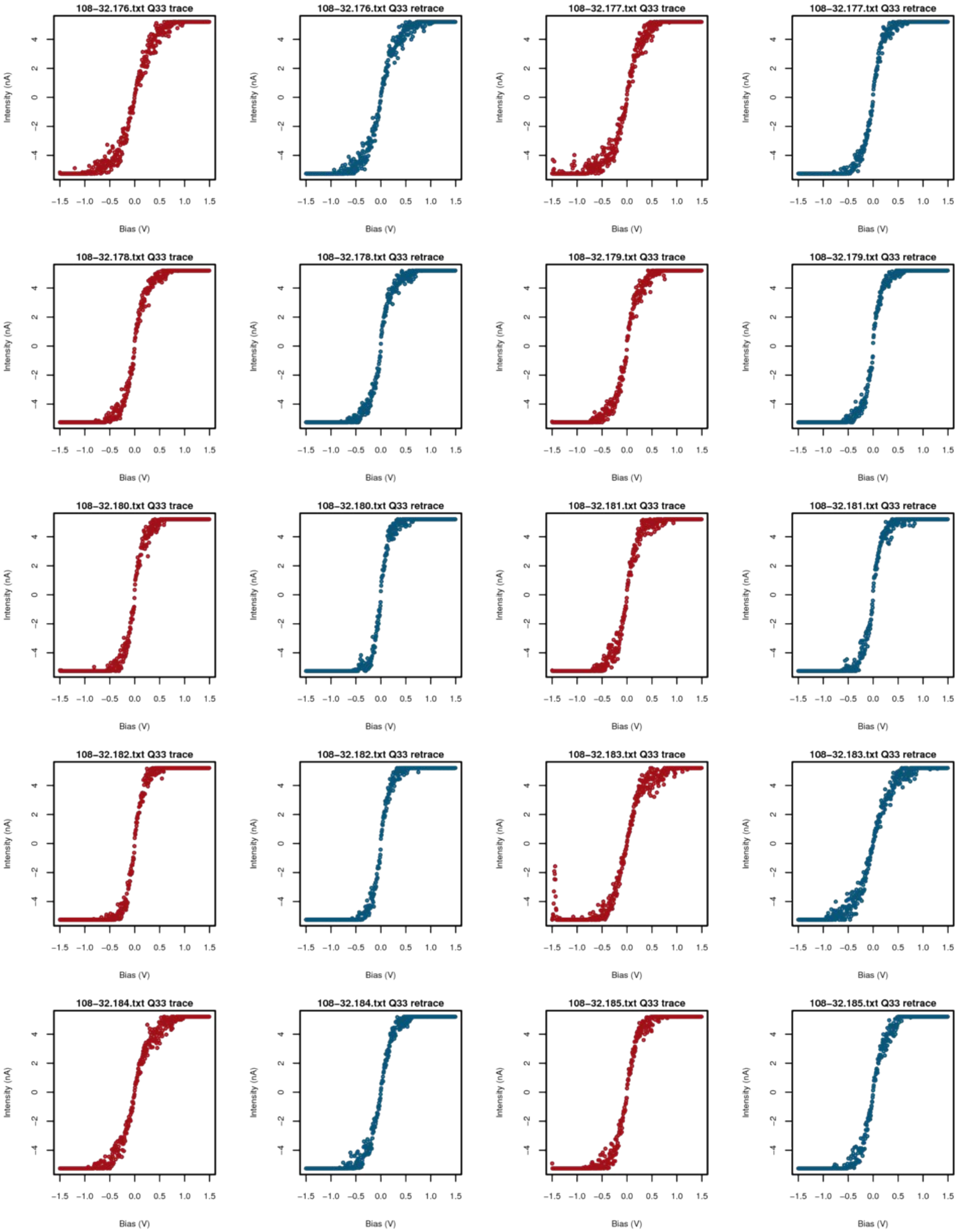

Continuation of Figure S38 (5/11)

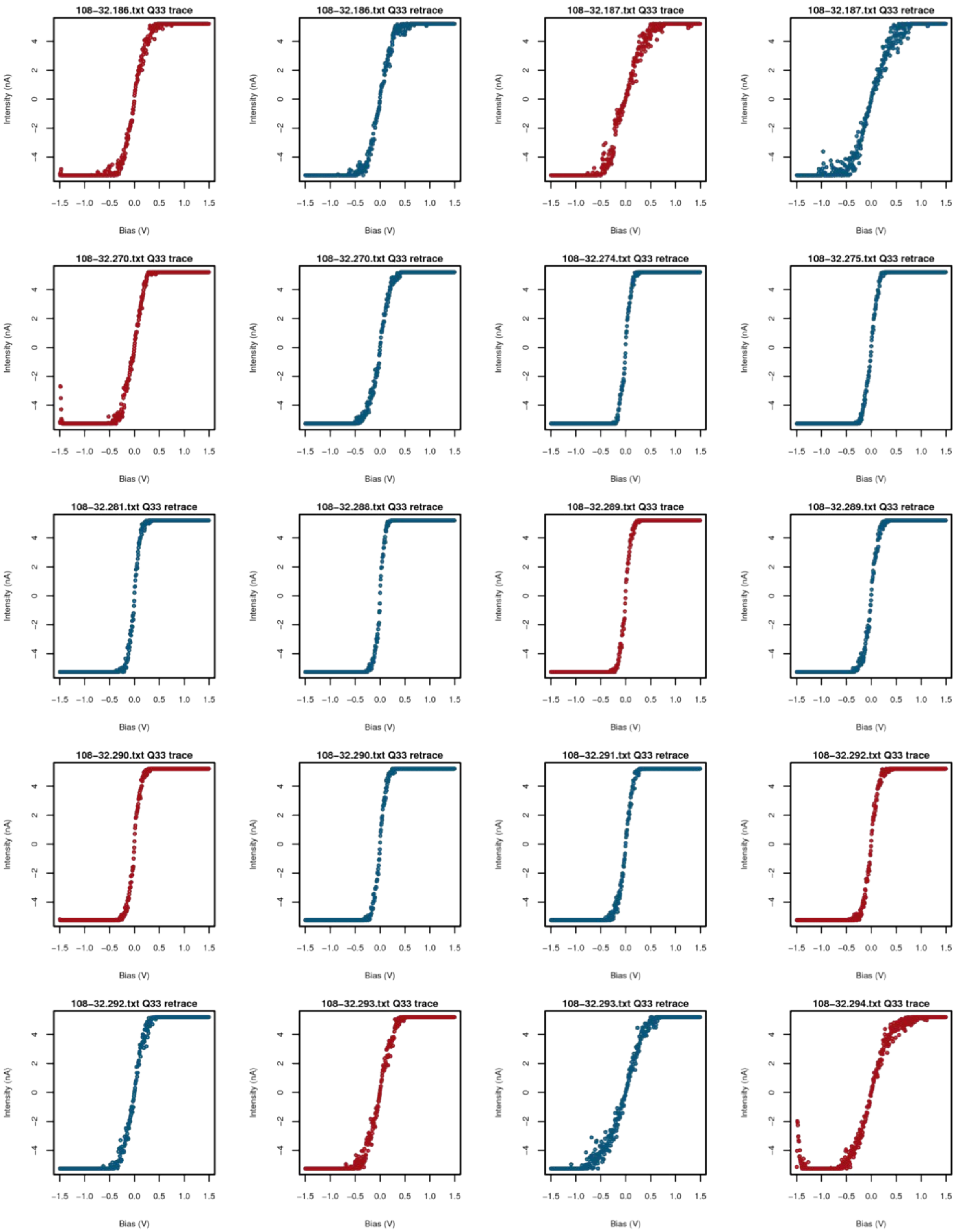

Continuation of Figure S38 (6/11)

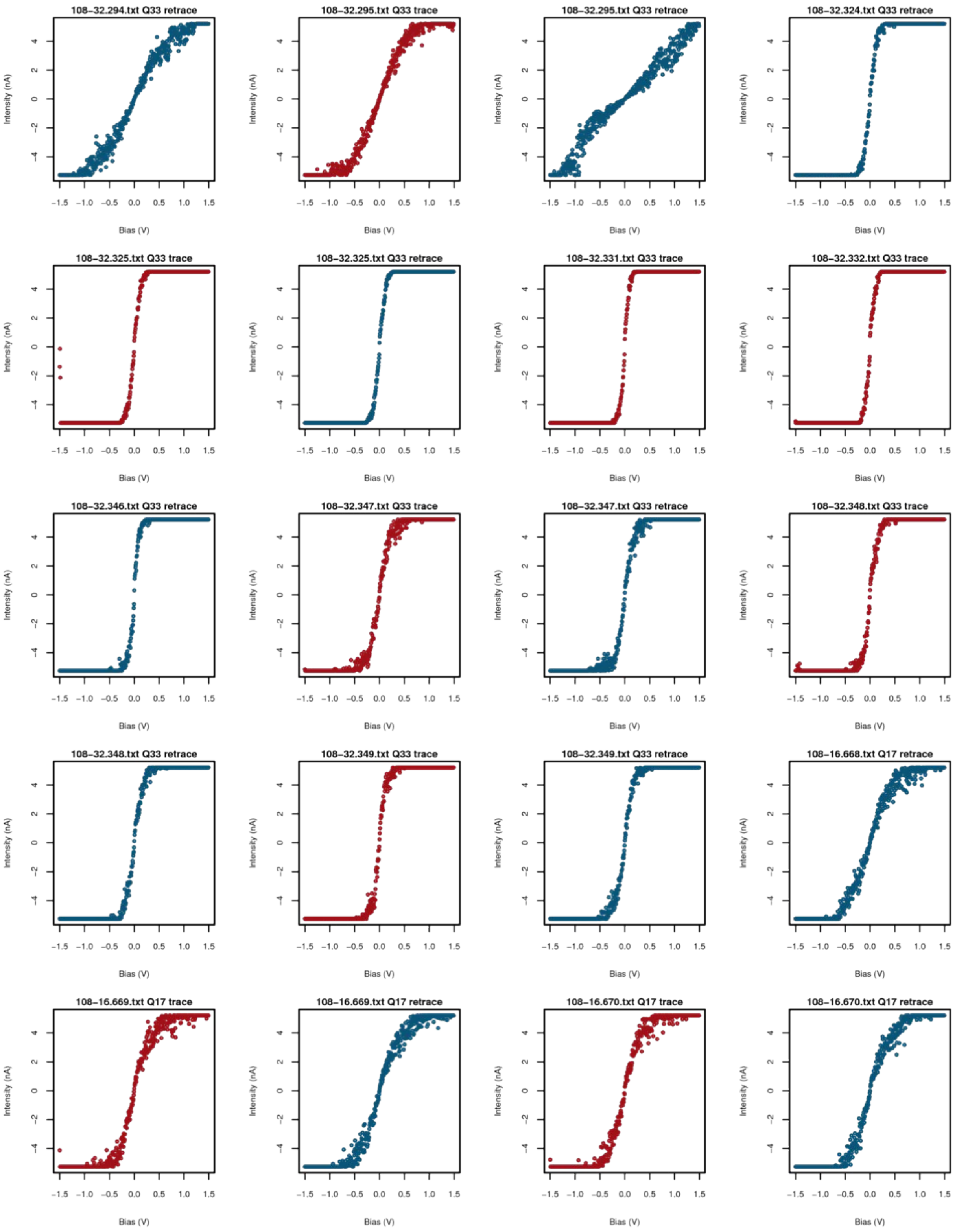

Continuation of Figure S38 (7/11)

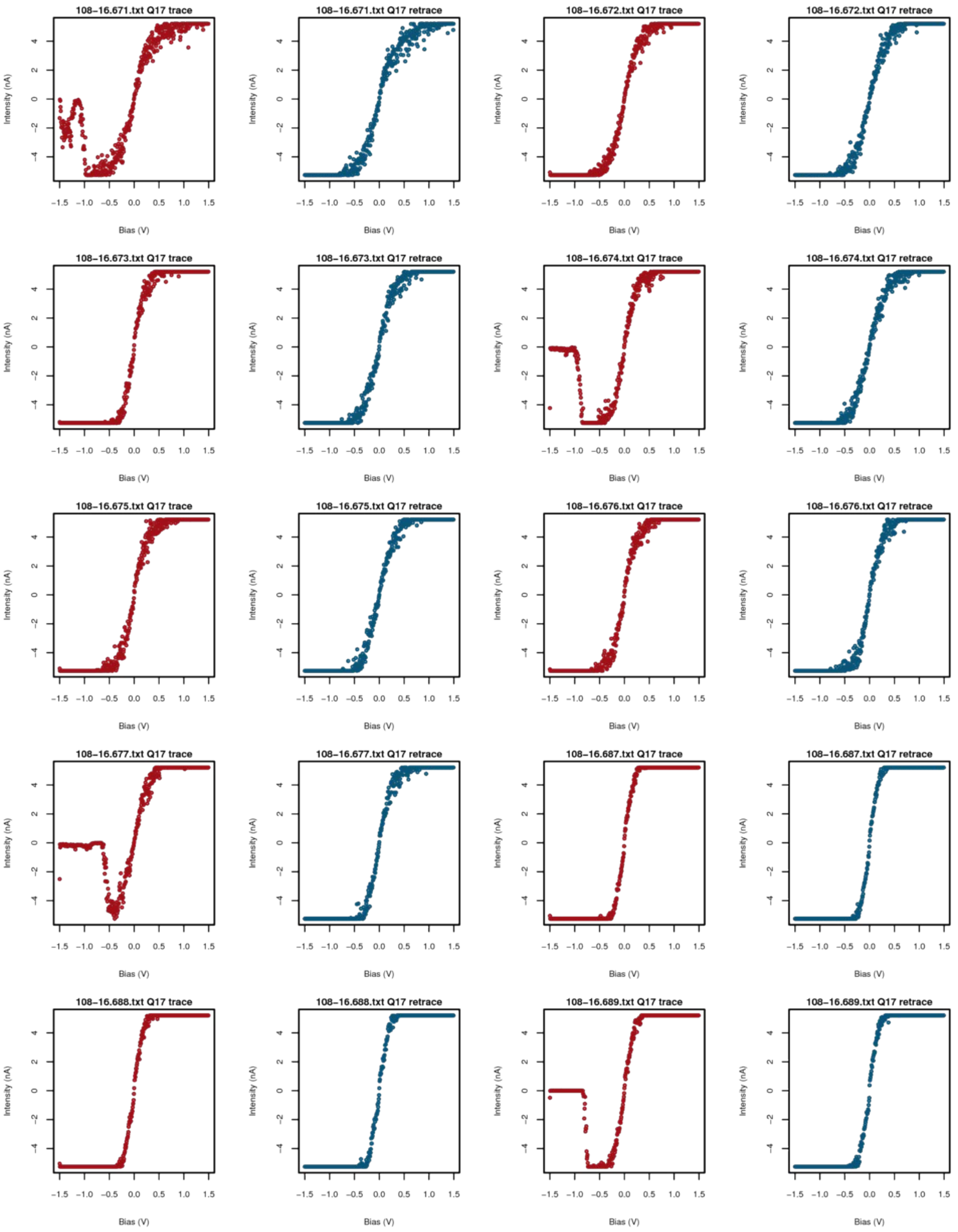

Continuation of Figure S38 (8/11)

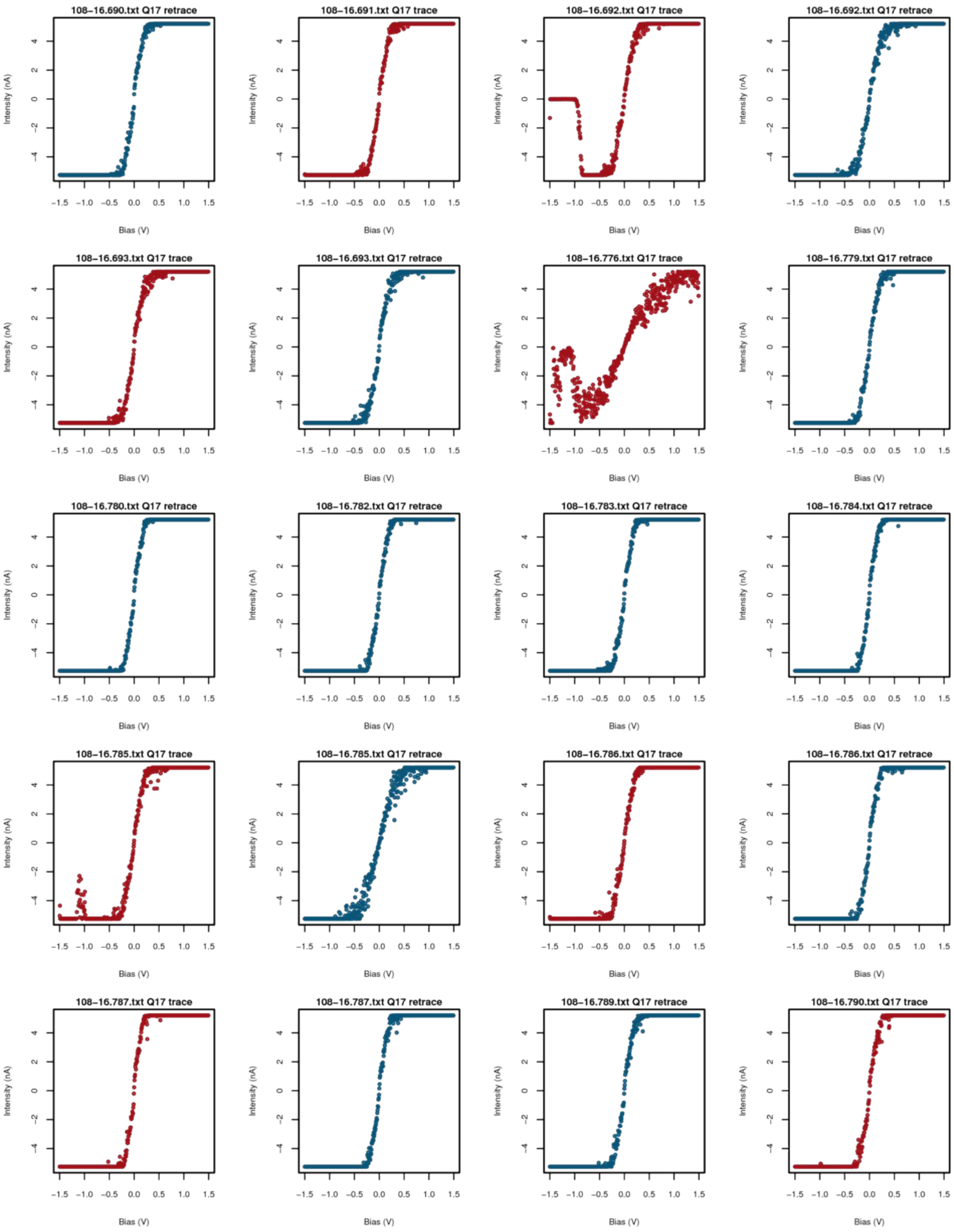

Continuation of Figure S38 (9/11)

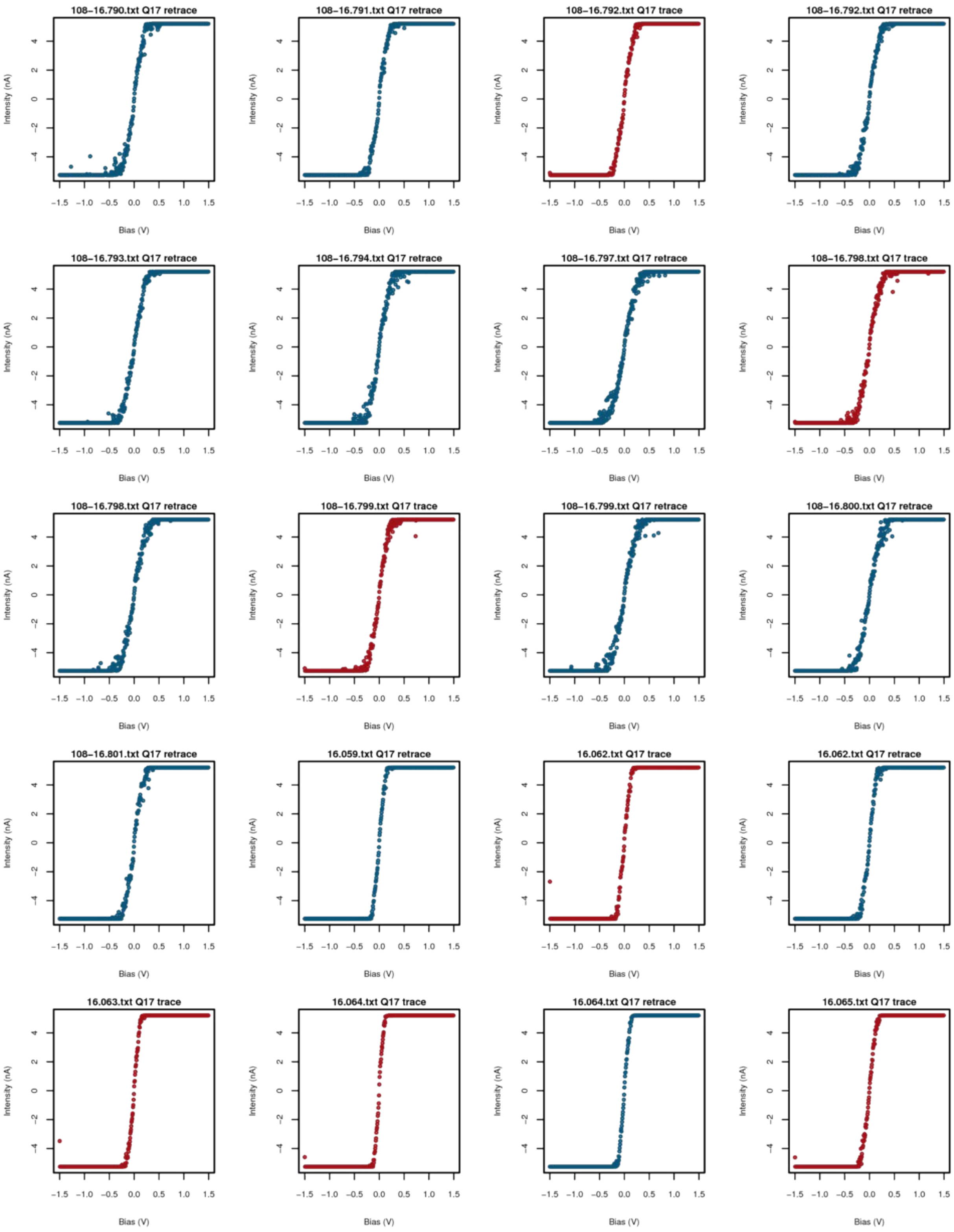

Continuation of Figure S38 (10/11)

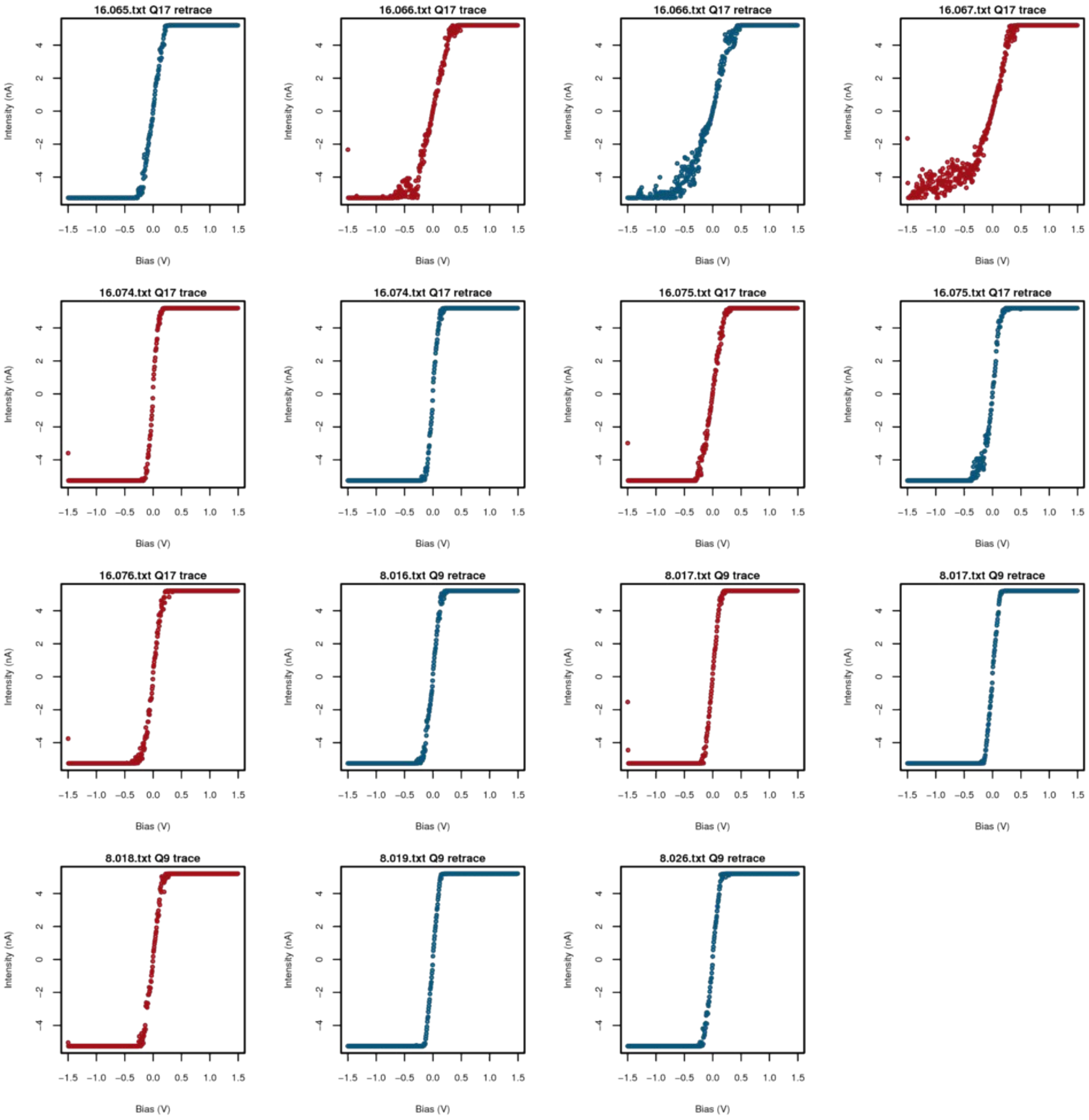

Continuation of Figure S38 (11/11)

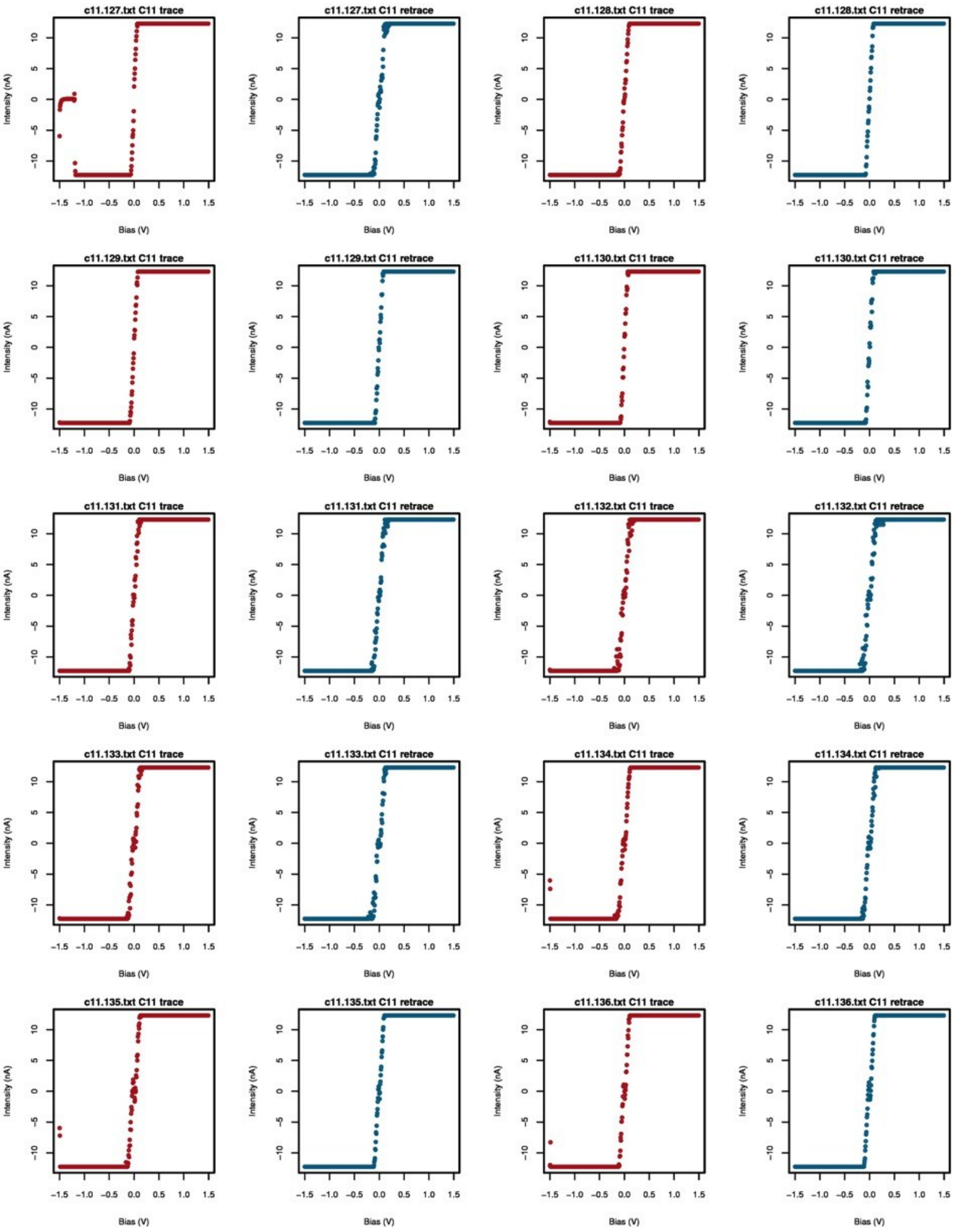

Figure S39. *I-V* curves acquired for azide monolayers on SiO_2. Red and blue points represent data acquired by reversing the bias forward and backwards.

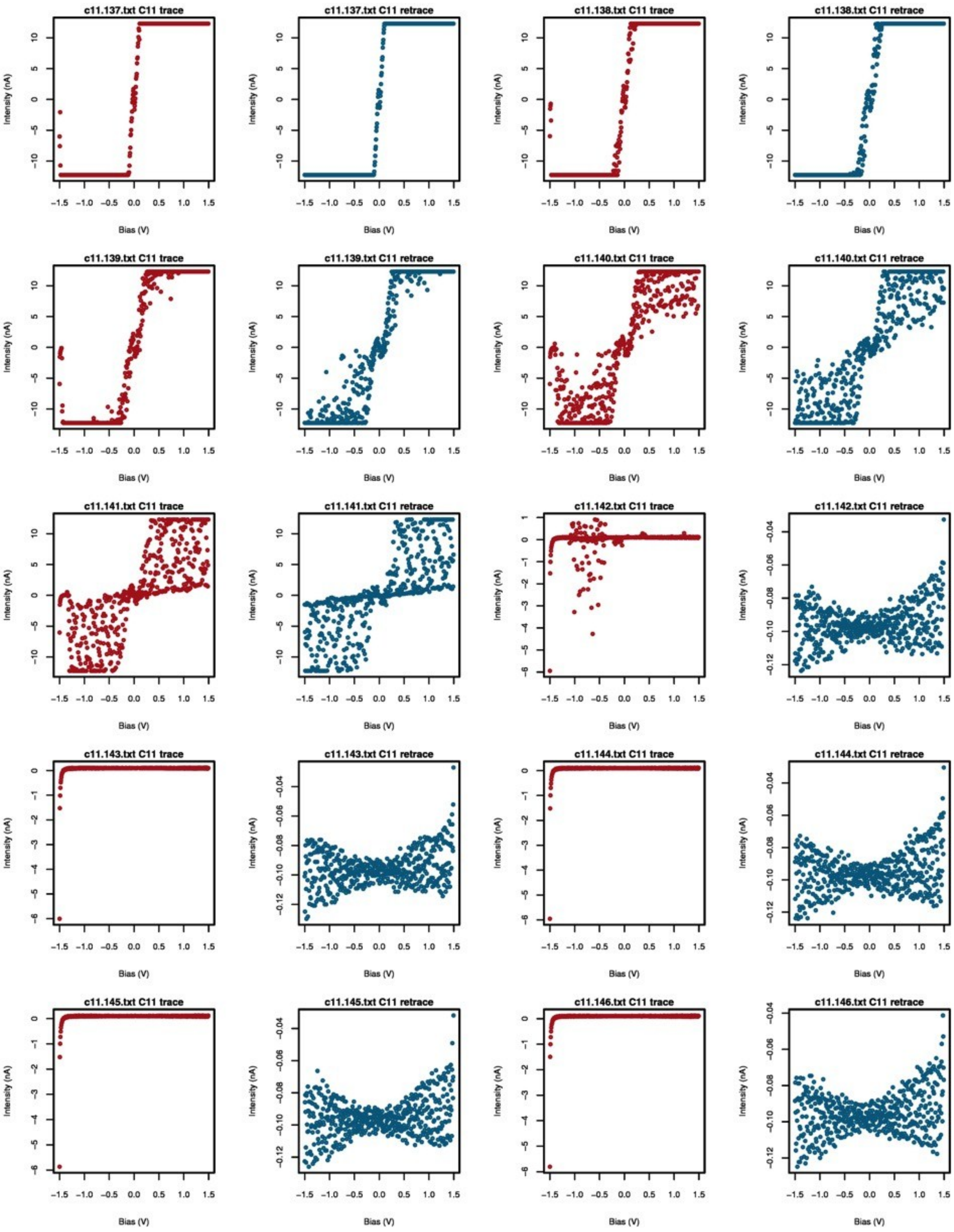

Continuation of Figure **S39**(2/2)

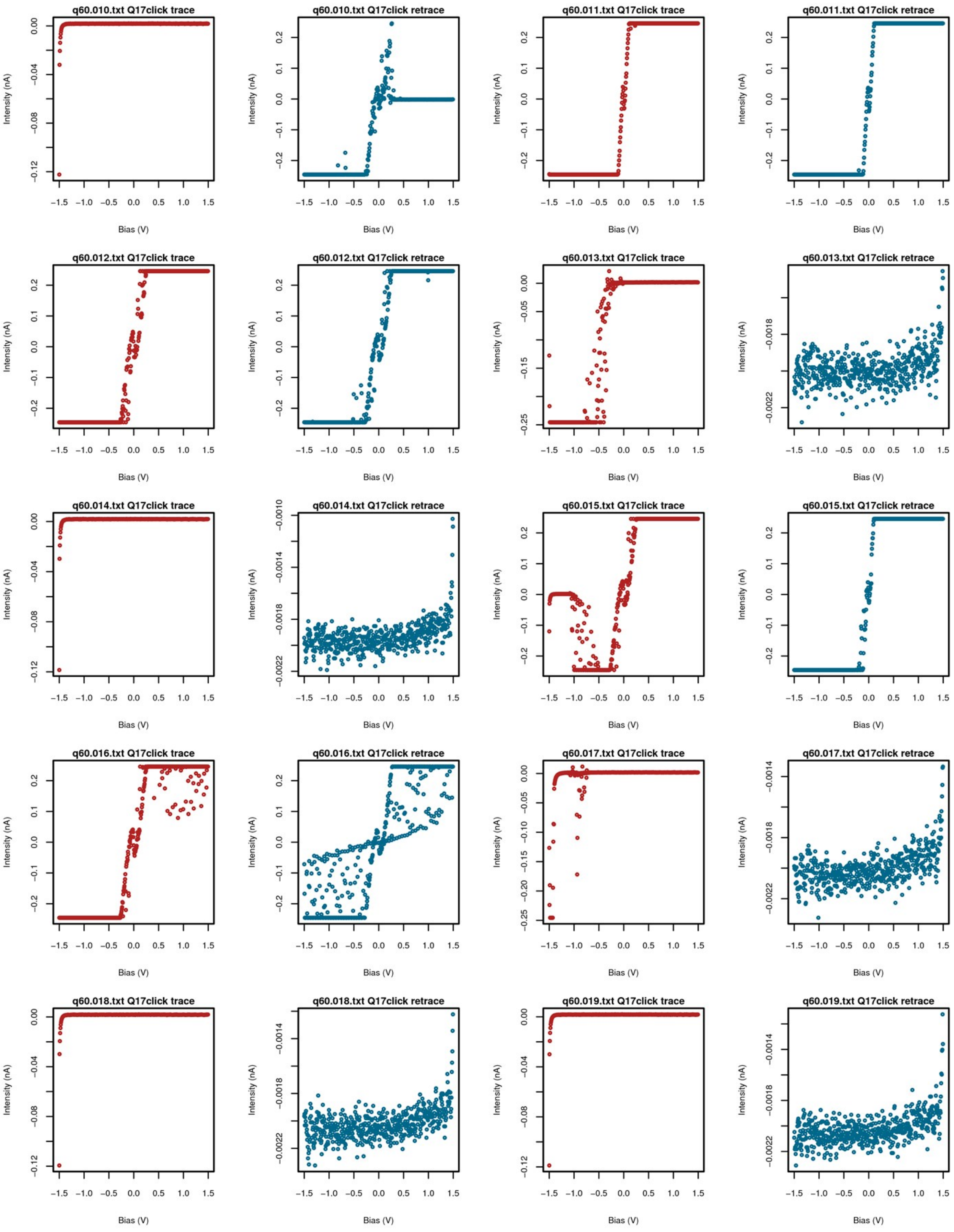

Figure S40. *I-V* curves acquired for monolayers of clicked AQ17 on SiO_2. Red and blue points represent data acquired by reversing the bias forward and backwards.

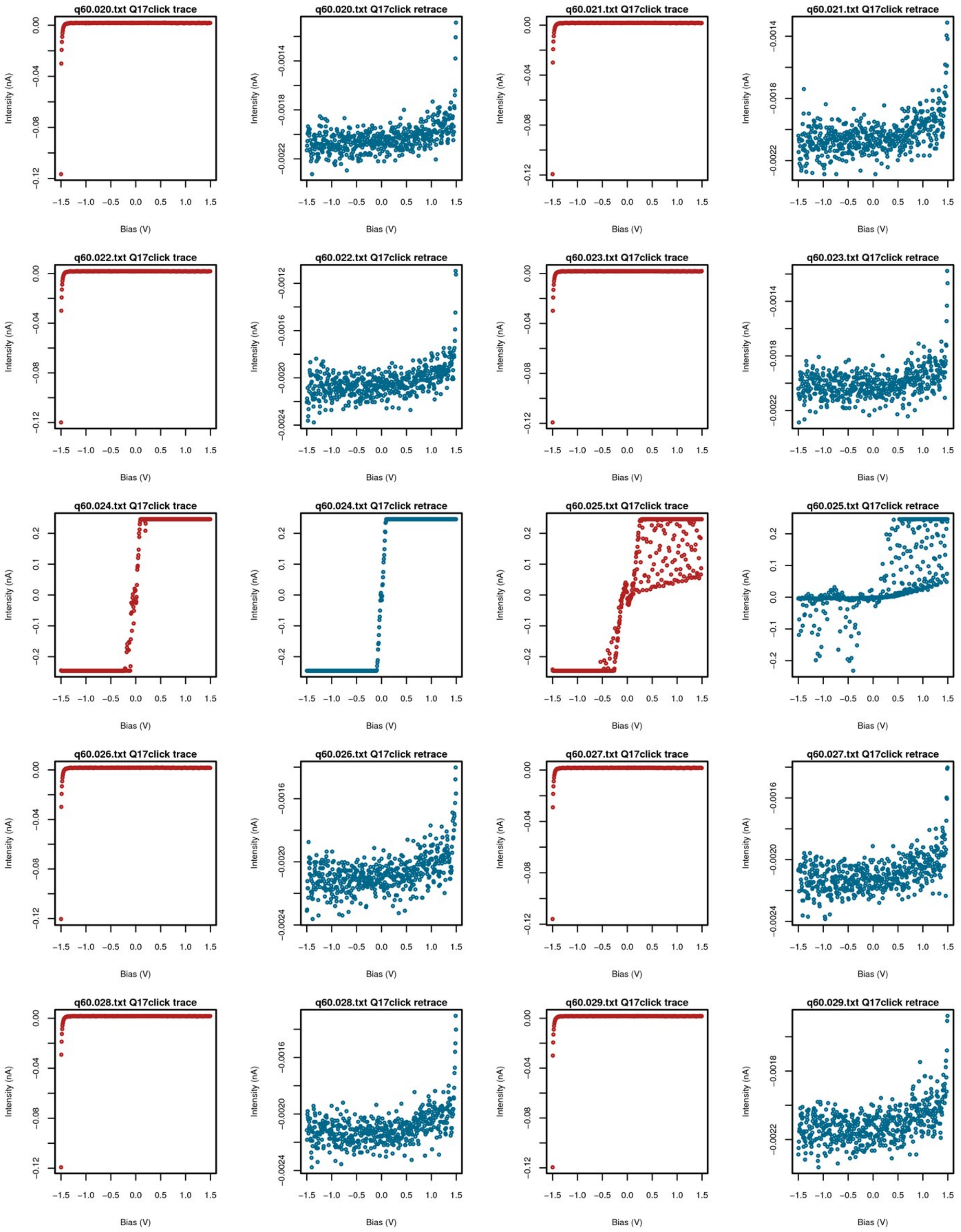

Continuation of figure S40(2/10)

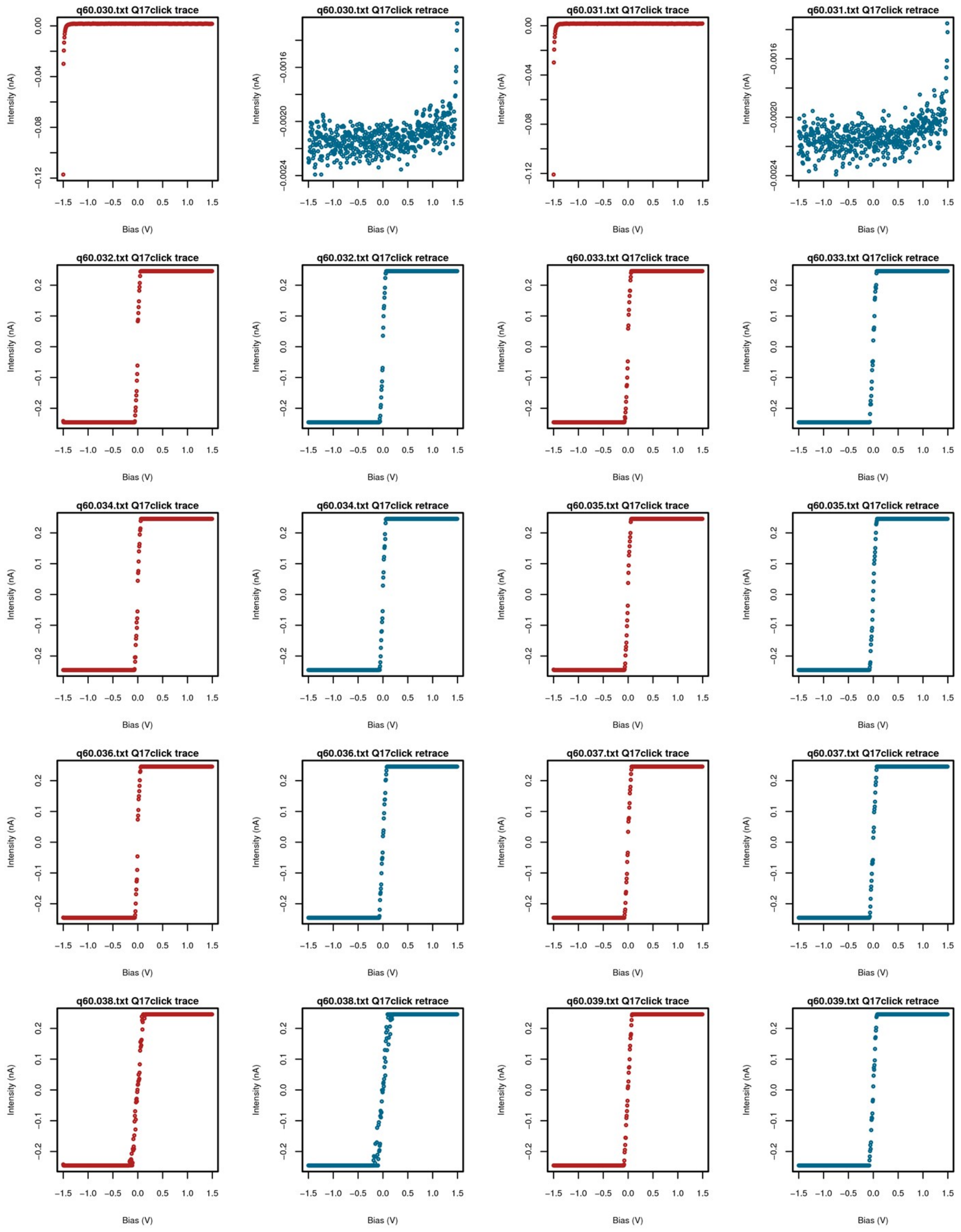

Continuation of figure S40(3/10)

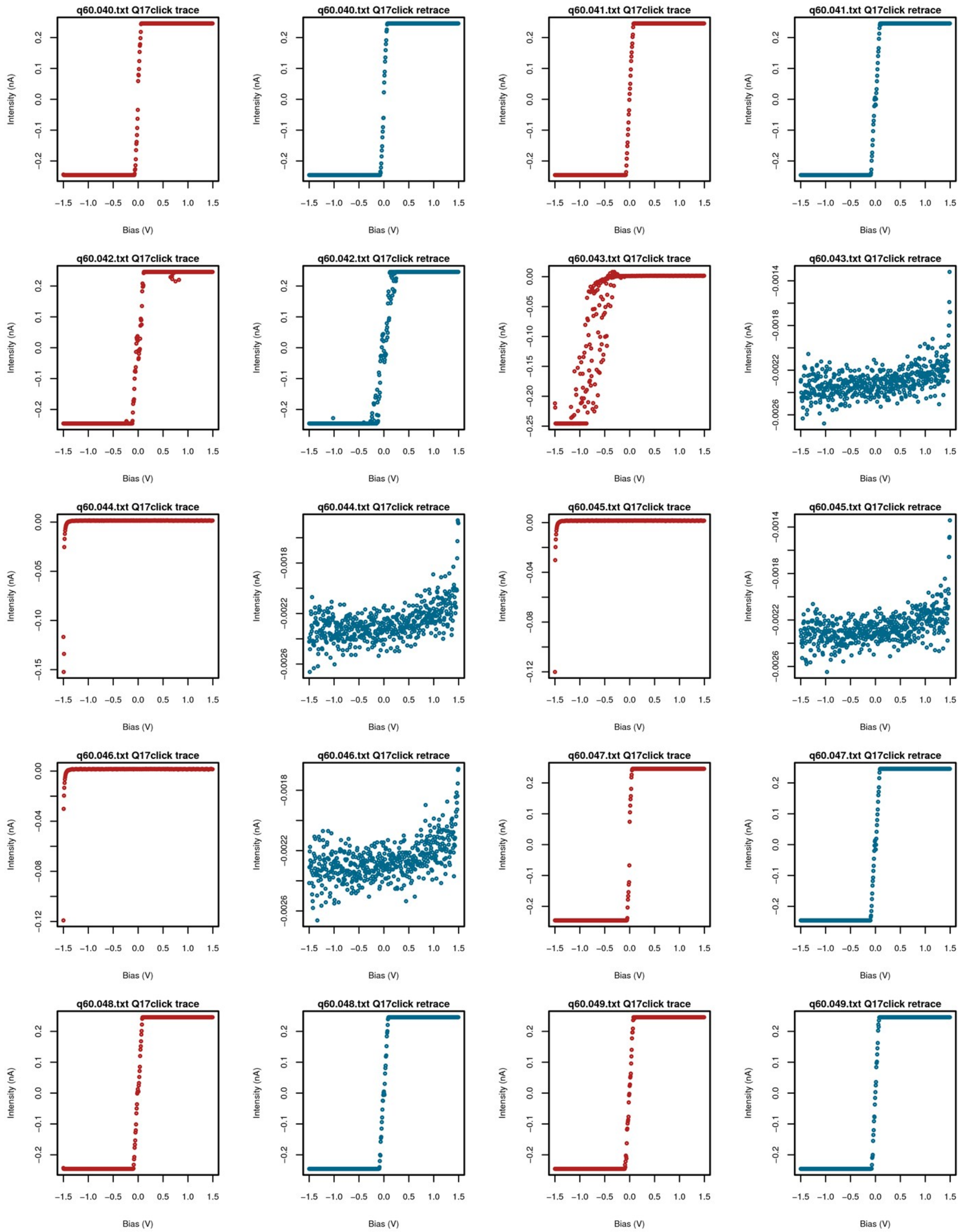

Continuation of figure S40(4/10)

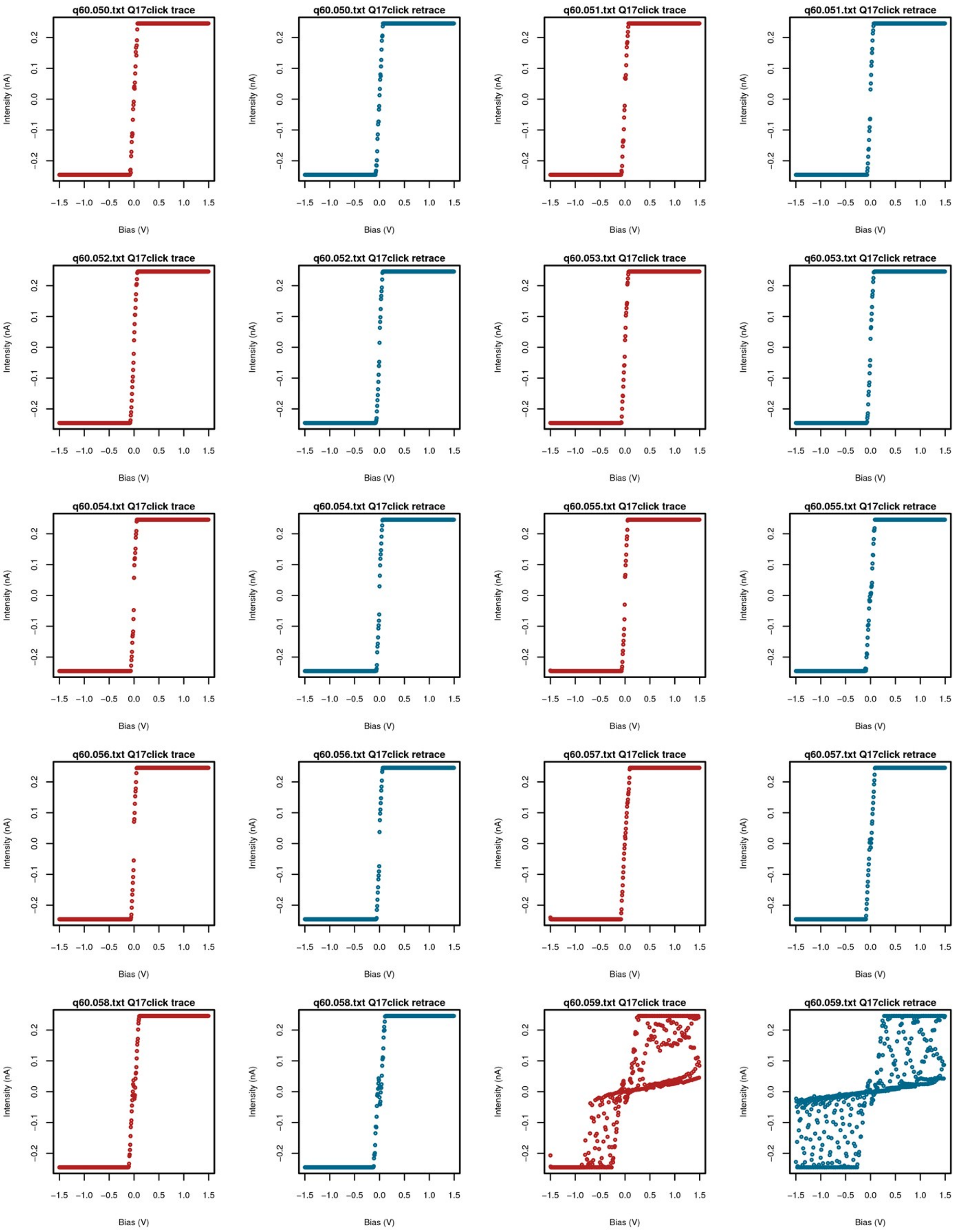

Continuation of figure S40(5/10)

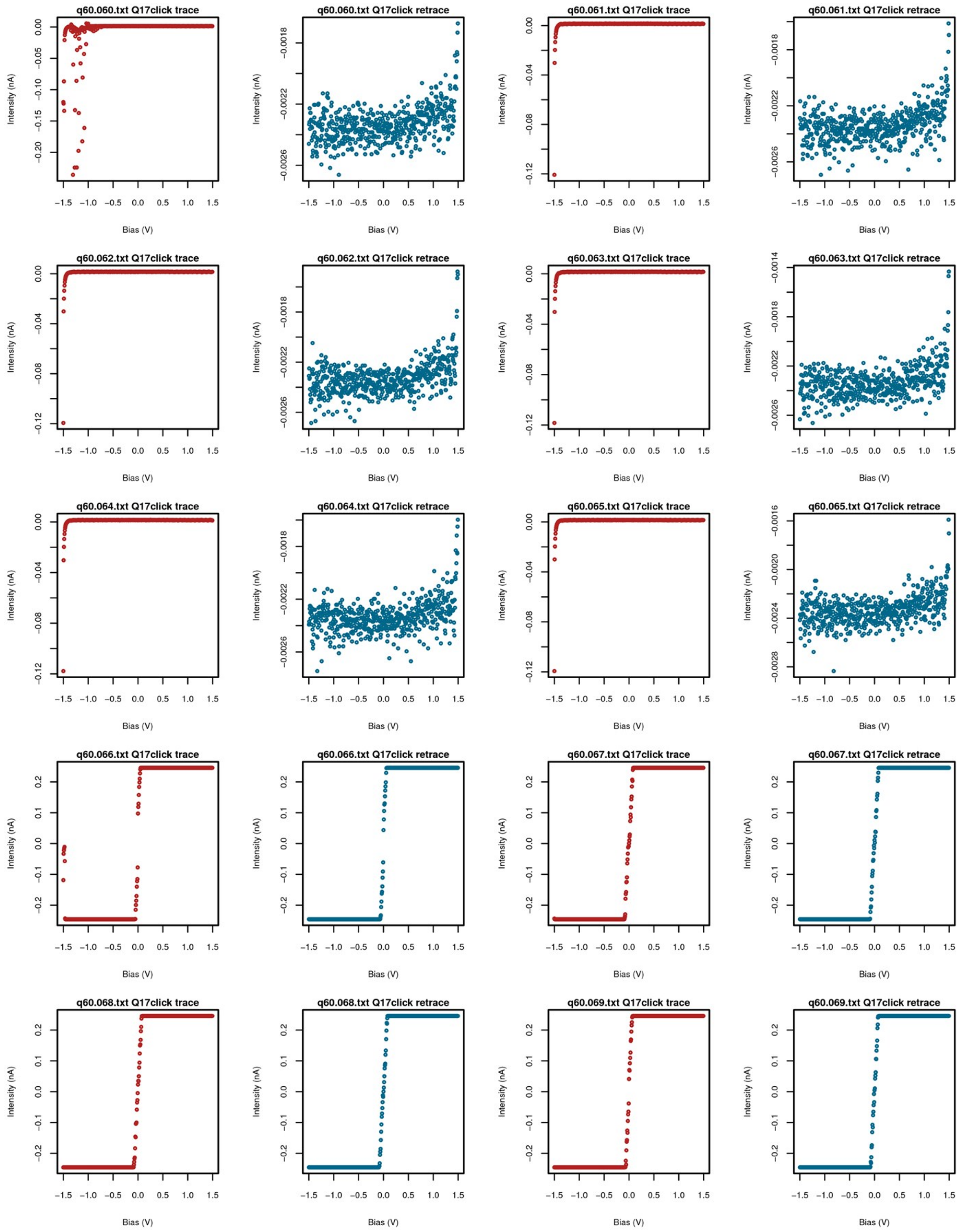

Continuation of figure S40(6/10)

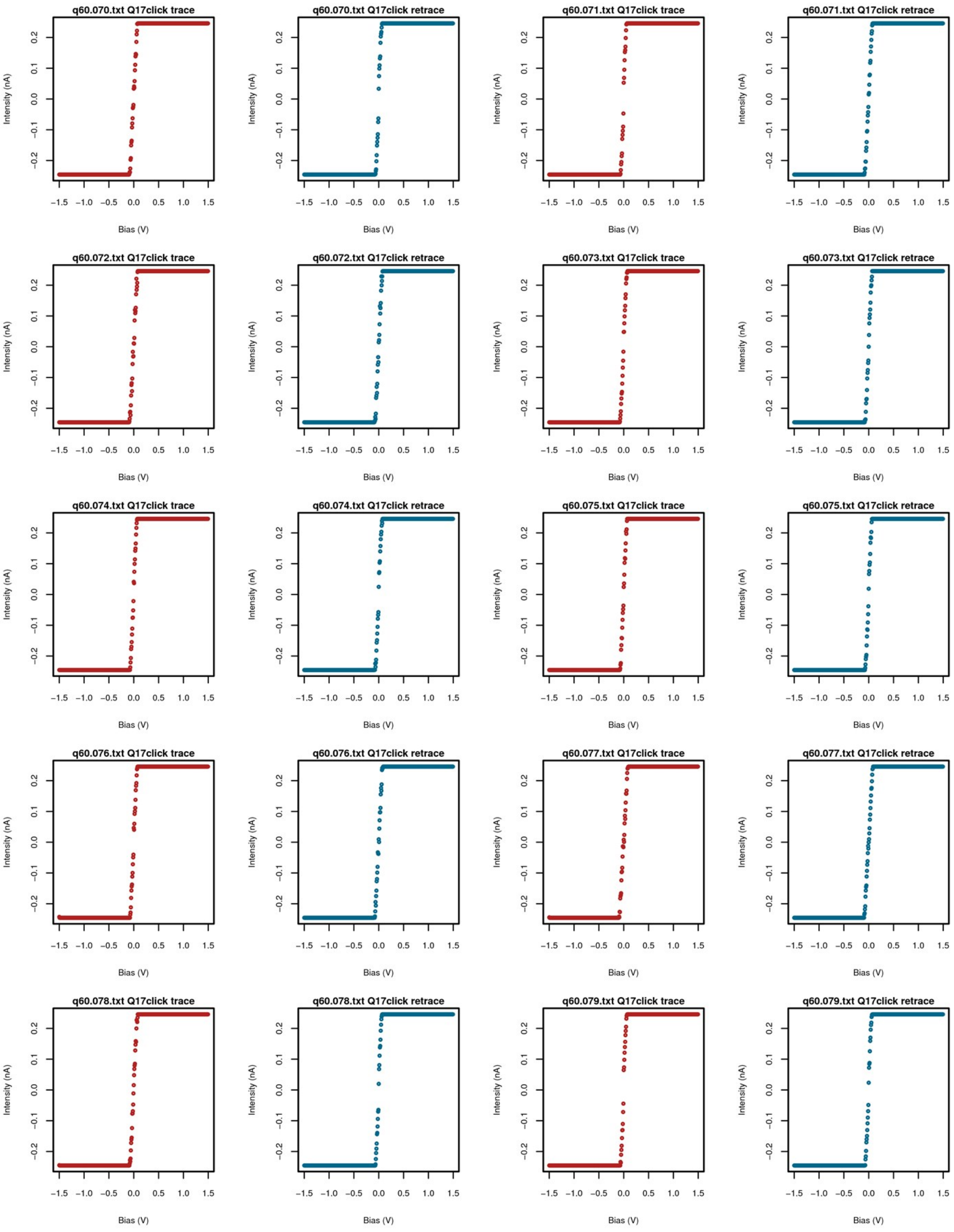

Continuation of figure S40(7/10)

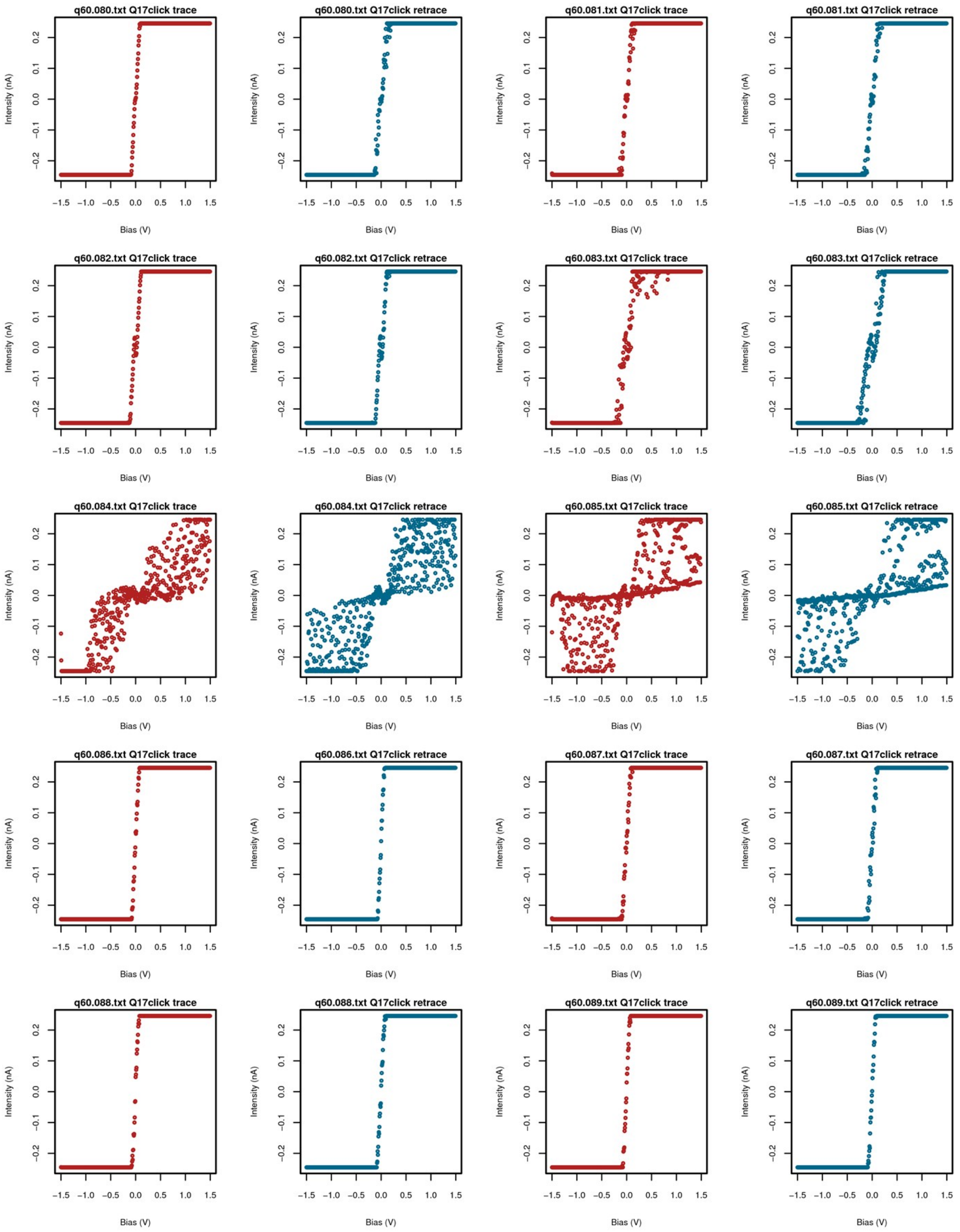

Continuation of figure S40(8/10)

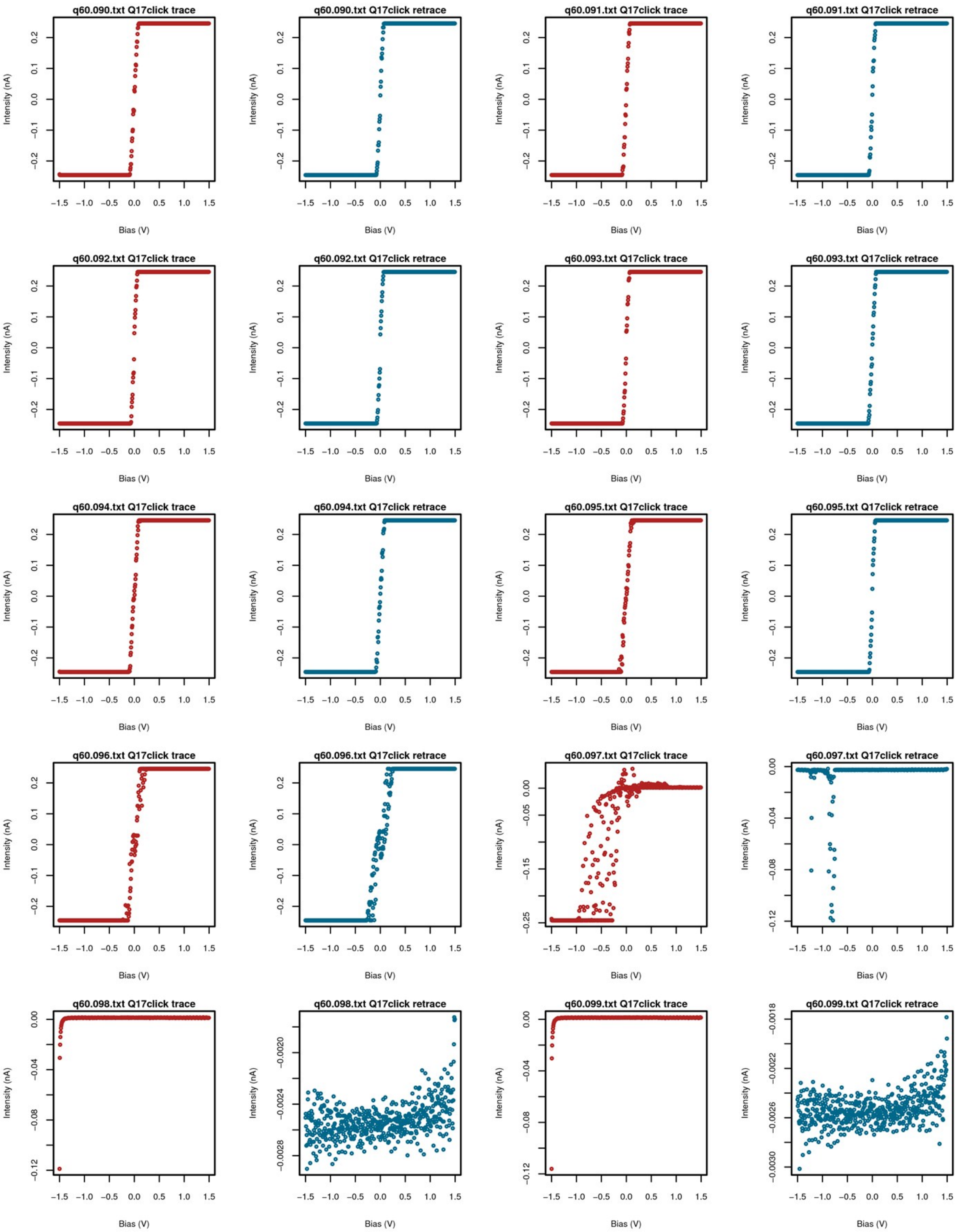

Continuation of figure S40(9/10)

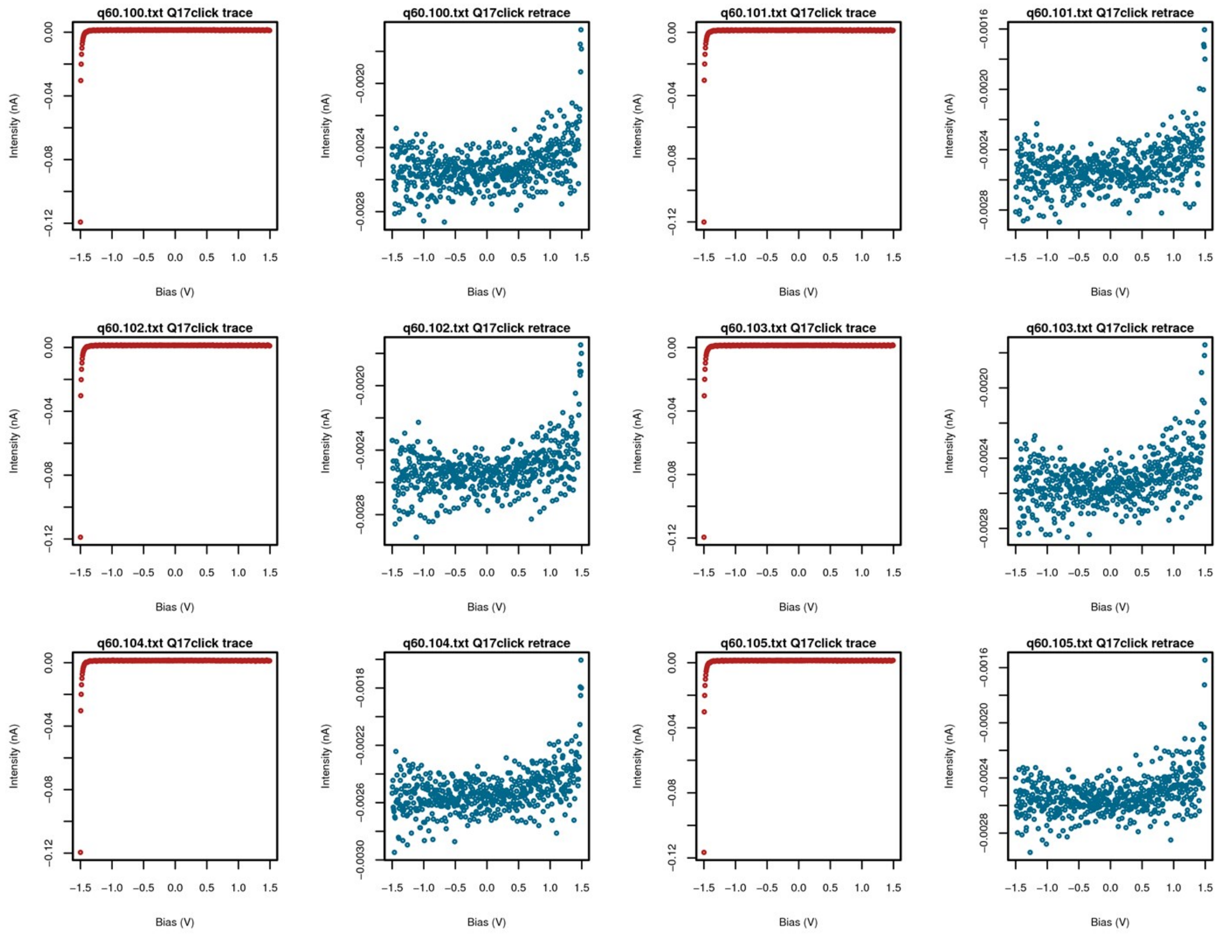

Continuation of figure S40(10/10)

References

[1]N. D. Heindel, T. A. Brodof, J. E. Kogelschatz, *J. Heterocycl. Chem.* **1966,** *3,* 222-223.
[2] X. Li, T. Qi, K. Srinivas, S. Massip, V. Maurizot, I. Huc, *Org. Lett.* **2016,** *18,* 1044-1047.
[3] T. Qi, T. Deschrijver, I. Huc, *Nat. Protocols* **2013,** *8,* 693-708.
[4] C. E. Inman, S. M. Reed, J. E. Hutchison, *Langmuir* **2004,** *20,* 9144-9150.
[5] T. Buffeteau, B. Desbat, J. M. Turlet, *Appl. Spectrosc.* **1991,** *45,* 380-389.
[6] M. A. Ramin, G. Le Bourdon, N. Daugey, B. Bennetau, L. Vellutini, T. Buffeteau, *Langmuir* **2011,** *27,* 6076-6084.
[7] T. Buffeteau, B. Desbat, D. Blaudez, J. M. Turlet, *Appl. Spectrosc.* **2000,** *54,* 1646-1650.
[8] R Core Team, R: A Language and Environment for Statistical Computing, **2015.**
[9] H. Wickham, *ggplot2: Elegant Graphics for Data Analysis,* Springer New York, **2009.**
[10] S. Højsgaard, U. Halekoh, doBy: Groupwise Statistics, LSmeans, Linear Contrasts, Utilities, **2016.**
[11] C. Wilke, cowplot: Streamlined Plot Theme and Plot Annotations for 'ggplot2', **2016.**
[12] W. F. Reus, C. A. Nijhuis, J. R. Barber, M. M. Thuo, S. Tricard, G. M. Whitesides, *J. Phys. Chem. C* **2012,** *116,* 6714-6733.
[13] M. Frisch, G. Trucks, H. Schlegel, G. Scuseria, M. Robb, J. Cheeseman, G. Scalmani, V. Barone, B. Mennucci, G. Petersson, H. Nakatsuji, M. Caricato, X. Li, H. Hratchian, A. Izmaylov, J. Bloino, G. Zheng, J. Sonnenberg, M. Hada, M. Ehara, K. Toyota, R. Fukuda, J. Hasegawa, M. Ishida, T. Nakajima, Y. Honda, O. Kitao, H. Nakai, T. Vreven, J. Montgomery Jr., J. Peralta, F. Ogliaro, M. Bearpark, J. Heyd, E. Brothers, K. Kudin, V. Staroverov, R. Kobayashi, J. Normand, K. Raghavachari, A. Rendell, J. Burant, S. Iyengar, J. Tomasi, M. Cossi, N. Rega, J. Millam, M. Klene, J. Knox, J. Cross, V. Bakken, C. Adamo, J. Jaramillo, R. Gomperts, R. Stratmann, O. Yazyev, A. Austin, R. Cammi, C. Pomelli, J. Ochterski, R. Martin, K. Morokuma, V. Zakrzewski, G. Voth, P. Salvador, J. Dannenberg, S. Dapprich, A. Daniels, Ö. Farkas, J. Foresman, J. Ortiz, J. Cioslowski, D. Fox, Gaussian09, Revision E.01, Gaussian Inc. Wallingford CT **2009**.
[14] C. Adamo, V. Barone, *J. Chem. Phys.* **1999,** *110,* 6158.
[15] S. Grimme, S. Ehrlich, L. Goerigk, *J. Comput. Chem.* **2011,** *32,* 1456-1465.
[16] J.-L. Brédas, D. Beljonne, V. Coropceanu, J. Cornil, *Chem. Rev.* **2004,** *104,* 4971-5004.
[17] F. Neese, *WIREs Comput. Mol. Sci. 2012* **2012,** *2,* 73-78.
[18] E. F. Valeev, V. Coropceanu, D. A. da Silva Filho, S. Salman, J.-L. Brédas, *J. Am. Chem. Soc.* **2006,** *128,* 9882-9886.
[19] D. T. Gillespie, *Annu. Rev. Phys. Chem.* **2007,** *58,* 35-55.
[20] A. Massé, R. Coehoorn, P. A. Bobbert, *Phys. Rev. Lett.* **2014,** *113,* 116604.
[21] M. D. Porter, T. B. Bright, D. L. Allara, C. E. D. Chidsey, *J. Am. Chem. Soc.* **1987,** *109,* 3559-3568.
